# Agro-Food Waste for Isolation of Non-Conventional Yeasts and Flavor Compounds Production

**DOI:** 10.3390/foods15081445

**Published:** 2026-04-21

**Authors:** Floriana Boscaino, Elena Ionata, Loredana Marcolongo, Davide Camerlengo, Alida Sorrentino

**Affiliations:** 1 Institute of Food Sciences, National Research Council (ISA-CNR), Via Roma 64, 83100 Avellino, Italy; floriana.boscaino@isa.cnr.it; 2 Research Institute on Terrestrial Ecosystems, National Research Council (IRET-CNR), 80131 Naples, Italy; elena.ionata@cnr.it (E.I.); loredana.marcolongo@cnr.it (L.M.); 3 Oasis Srl, 83030 Torre Le Nocelle, Italy; d.camerlengo@oasis-srl.it

**Keywords:** food waste, non-conventional yeast, fermentation, volatile organic compounds

## Abstract

The transition towards a circular bioeconomy is essential to mitigate the environmental pressures caused by the increasing global demand for food and energy. Agro-food waste (AFW) is a plentiful, inexpensive feedstock, exploitable in biorefineries to produce valuable molecules. The aim of this study was to isolate native non-conventional yeasts (NCY) from various AFW and to evaluate their potential for the ‘natural’ synthesis of aroma compounds via fermentation. Ten strains were isolated and identified as belonging to *Saccharomyces cerevisiae*, *Pichia kluyveri*, *Pichia californica* and *Wickerhamomyces anomalus* species. The fermentative performance and production of aroma volatile compounds were tested using different household wastes as substrates. Figs containing substrate, which is the richest in fermentable sugars, allowed for the fastest microbial adaptation and highest yields of volatile compounds. HS-SPME-GC/MS analysis revealed that the most prominent compounds were isoamyl alcohol, ethyl acetate and isoamyl acetate with the highest production levels showed by *W. anomalus* YDSCYP4 and *P. kluyveri* YDSCYP5. Enzymatic profiling revealed significant arylamidase and esterase activities in the selected strains, related to their role in the hydrolysis of aroma precursors. These findings demonstrate the efficiency of these autochthonous yeasts for the sustainable production of aroma compounds, supporting the development of eco-friendly biotechnological processes.

## 1. Introduction

The transition towards a bio-based economy is among the most urgent priorities to avoid unsustainable life prospects for future generations [1]. The need for increasing quantities of water, petroleum-derived energy and food, to feed up to 9.1 billion people by 2050 prompts a rethought approach to the exploitation of the terrestrial resources and biomass processing. In order to feed a projected population of 9.1 billion by 2050, we must increase our reserves of water, oil-derived energy and food. This requires to reconsider our approach to using the Earth’s resources and processing biomass. The new circular bioeconomy model, based on the valorization of agro-food waste (AFW), abundant, cheap and renewable materials not in competition with human food, allows the reduction in the heavy environmental pressure of the traditional economy that causes ecosystems pollution, greenhouse gas emission, water and soil re-sources depletion [2]. Biorefineries use environmentally friendly technologies to convert AFW into bioenergy, bioactive molecules, chemicals, biomaterials and food. Biorefineries employ eco-friendly technologies to convert AFW into bioenergy, bioactive molecules, chemicals, biomaterials, and food [3,4].

Following the prevision of FAO (Food and Agriculture organization), [1], the increase in agricultural production between 2012 and 2050 that will be of 35–50% will also produce raising amounts of AFW. One third of waste is currently 1.3 billion tons per year, half of which comes from fruit and vegetables. This creates environmental pressure, economic losses for producers and ethical issues [5]. Losses in food supply occur due to technological limits, e.g., after harvest and during processing/distribution in developing countries. In the developed world, significant quantities of products are discarded at the final production stage due to high market standards [6].

Food waste according to the European project FUSION (Food Use for Social Innovation by Optimising Waste Prevention Strategies) is defined as “any food, and inedible parts of food, removed from the food supply chain to be recovered or disposed of”. AFW, rich in proteins, lipids, carbohydrates and more, serves as ideal feedstock for biorefining via green biotechnologies like enzymatic/microbiological transformations, with fermentation playing a pivotal role [3,7]. Fermentation is an ancient technology [8], dating back to about several thousand years ago, utilized to produce foods and beverages such as bread, wine, beer, and milk, vegetable and meat derived foods [9,10]. Recently fermentative technologies have been widely utilized to valorize AFW in industry exploiting microbial biodiversity, from bacteria to fungi (especially yeasts), with the recovery and biosynthesis of valuable bioactive compounds [11].

Yeasts, [9] unicellular chemoorganotrophs, ferment sugars anaerobically (producing CO_2_ and ethanol) or under oxygen via the Crabtree effect, dominating sugar-rich niches like ripening fruits despite ethanol toxicity. In those environments, yeasts trigger fermentative processes of exuded sugary juices production. In these environments, yeasts trigger the fermentation of sugary juices and, beyond the primary metabolites (CO_2_ and ethanol), a wide array of secondary metabolites (aldehydes, ketones, esters, organic acids, terpenes and other compounds). Other yeast species, instead, promote food spoilage and facilitate the action of microorganisms such as filamentous fungi and aerobic and anaerobic bacteria [4,12] that carry out biomass decomposition [5,6,13]. *Saccharomyces* “sensu stricto” species (e.g., *S. cerevisiae*, *S. bayanus*, *S. paradoxus*, *S. pastorianus*) excel in ethanol production and are the most widely utilized for foods and beverages production, due to their vigorous fermentative capacity, elevated growth rate and ethanol tolerance [14]. Non-conventional yeasts (NCY), e.g., *Debaryomyces*, *Kluyveromyces*, *Pichia*, *Candida*, offer metabolic versatility under stressful conditions (high temperature, extreme pH values and hyperosmotic or oxidative conditions) [15,16], their complex and interesting enzymatic activities that allow them to grow on a wide range of different substrates, and produce secondary metabolites like volatile aroma compounds (aldehydes, ketones, esters, lactones, acids, terpenes), they are ideal for several biotechnological applications. NCY’s unique metabolic processes can produce bio-technologically relevant compounds from AFW and recycle them using biorefining approaches [17].

Usually, aroma compounds are produced by extraction with conventional solvent systems from plants or by chemical synthesis from petroleum-based compounds (80% of commercialized products). However, due to the increasing consumer awareness of “natural” products, green biotechnologies are preferred [18]. The importance of ‘de novo synthesis’ achieved through fermentation technology allows the transformation of suitable precursors into defined aromatic compounds and bioconversion/biotransformation, single-phase/multi-phase biological processes, which use enzymes also expressed by engineered microorganisms, are widely recognized [3]. Fermentative processes, lead to the production of a mixture of aroma molecules, whose complexity can be modulated by using different microbial starters or different combinations of starters and substrates. NCY such as *Debaryomyces*, *Kluyveromyces*, *Pichia* and *Candida* spp. are among the best aroma producers utilizing agro-food waste as substrates in submerged or solid-state fermentation processes [19]. Compounds mainly belonging to alcohol and esters (isoamyl alcohol, phenylethyl alcohol, ethyl acetate, isoamyl acetate, 2-phenylethyl isobutyrate and phenylethyl acetate) with floral or fruity aromatic notes are the most prominent volatile compounds produced by fermentation of AFW [20]. Different authors reported NCY fermenting ability on various substrates, as in the case of *K. marxianus* that produced interesting aroma compounds mixtures when tomato, grape, and pepper pomace, rice bran, sugarcane bagasse and sugar beet molasses were harnessed as substrates. Also promising results were obtained when these biocatalysts were utilized in the immobilized form [6,7,16,17]. The highest production yields of specific aroma compounds, such as 2-phenylethanol, have been achieved utilizing *P. kudriavzevii*, which was isolated from fermented sugarcane bagasse. This non-*Saccharomyces* strain was exploited in solid-state fermentation utilizing sugarcane bagasse and sugar beet as substrates and phenylalanine as a specific precursor [8,18]. In the context of climate change, the increasing demand of yeasts strains with superior, innovative features or endowed with specific metabolic traits for the adaptation to a particular bio-process designs, are prompting the scientific community to find these microorganisms in new and unconventional habitats. Thus, the increasing interest for non-*Saccharomyces* yeasts that are able to offer intrinsically different sensorial profiles that go beyond those achievable by the traditional *Saccharomyces* strains.

In this study, a targeted screening of yeast strains was conducted on real fruit and vegetable AFW at 28 °C, evaluating adaptive growth, primary metabolite yield, and secondary aroma compounds.

The substrate-matched yeasts isolation was the strength point of the strategy to pursue the best performances by leveraging on the selection of the best adapted microorganisms to the fermentation substrates. By exploiting the metabolic versatility of NCY, the way is paved for scalable biorefining of waste, thereby promoting the goals of a sustainable bioeconomy that goes beyond ethanol-focused processes.

## 2. Materials and Methods

### 2.1. Sample Collection from Fruit and Vegetables Waste

The fruit and vegetable waste samples were collected from agricultural producers of different villages located at different altitudes, between Avellino and Benevento provinces, in Campania region. In detail, the plums, apples and potatoes from Altavilla Irpina at 334 m a.b.s; figs and celery from Gesualdo at 676 m a.b.s; kiwi from San Mango sul Calore at 470 m a.b.s.; figs and bunch grapes from Foglianise 350 m a.b.s; while the strawberry grapes were bought in the food shop and grape marc was collected from a wine cellar near Avellino city 348 m a.b.s. Samples were collected from fruit and vegetable waste represented by rotten and injured fruit, pulp and peels of apples, potatoes, grapes, kiwi, figs, stalks and leaves of celery.

**Figure 1:**
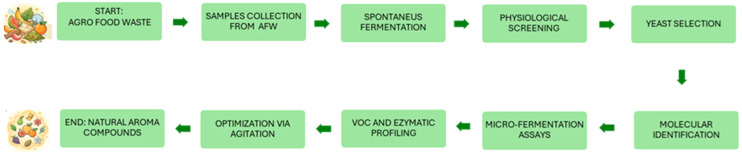
Flow diagram for yeast strains screening and AFW valorization.

Figure 1 shows a flowchart illustrating the strain selection rationale.

### 2.2. Spontaneous Fermentation Tests

Mixed fruit and vegetable waste, after grinding and homogenization, were placed in 1 L Erlenmeyer flasks, filled with 1:2 distilled water and utilized as substrates for spontaneous fermentation tests.

The different substrate mixes were composed as follows:

- substrate “a”: kiwi puree (30 g), apple peels (10 g), potatoes pulp and peels (20 g), grape pomace (40 g) and distilled water (200 mL).
- substrate “b”: celery leaves and stalks (20 g), rotten figs (60 g), rotten grape (20 g), and distilled water (200 mL).
- Substrate “c”: rotten strawberry grape (60 g), potatoes pulp and skins (40 g) and distilled water (200 mL).

Two flasks for each substrate were set up. All the flasks were incubated under static condition at 28 °C for 30 days. The kinetics of the batch fermentations were determined by measuring the weight loss of the entire set up on the same flask (flask 1) every day. Microbiological analyses were carried out on aliquots withdrawn at fixed time intervals times (after 0, 5, 10, 15, 20, 25 and 30 days) from the other flask (flask 2).

Spontaneous fermentation processes were carried out by incubating the Erlenmeyer flasks at 28 °C for 30 days under static conditions. The test was performed in duplicate.

### 2.3. Native Yeasts Isolation and Colony Counts

Samples (10 g), withdrawn in sterile conditions, from each fermented biomass at fixed time intervals (0 and 30 days), placed in sterile Stomacher bags, added with 90 mL sterile Ringer solution were homogenized for 1 min at 250 rpm, with Stomacher 400 Circulator (Seward, UK). Decimal dilutions (100 µL) prepared from homogenized samples (1 mL) were plated onto solid media. YPD agar (20 g L^−1^ bacteriological peptone, 20 g L^−1^, dextrose, 10 g L^−1^, yeast extract, 15 g L^−1^ agar), and WL agar (Wallersteins Laboratory nutrient agar) (Thermo-Fischer Scientific, Milan, Italy). The plates were incubated at 28 °C for 3–5 days. The analysis was performed in triplicate.

The enumeration of yeast populations grown during each waste biomass fermentation and the randomly isolation of colonies with different colors and morphology were carried out. In order to check their purity, single colonies were selected, streaked twice onto plates with the same media utilized for the isolation and then incubated at 28 °C for 3–5 days.

### 2.4. Microscope Analysis of Yeast Cell Morphology

The selected yeast strains were transferred from plates to liquid medium by inoculating test tubes containing 5 mL of YPD broth (20 g L^−1^ bacteriological peptone, 20 g L^−1^, dextrose, 10 g L^−1^, yeast extract) that were incubated at 28 °C for 24 h under static condition. After reinoculation and growth for 24 h, cell morphology and absence of microbial contamination were determined by phase-contrast microscope (N300B, Orma Eurotek, Scientific Istruments, Milan, Italy) analysis. The obtained pure cultures were transferred onto YPD slants that, after incubation at 28 °C for 3 days, were stored at 4 °C and preserved in a new culture collection, inside the ISA-CNR Institute’s yeast strains library for subsequent evaluation of physiological and technological features.

### 2.5. Carbohydrate Assimilation Tests

The ability of the native yeasts to ferment or assimilate various carbohydrates was tested by utilizing Yeast Nitrogen Base medium (YNB, Thermo-Fisher Scientific, Milan, Italy) and different carbohydrates such as glucose, maltose, fructose and sucrose (Sigma-Aldrich, Milan, Italy). In detail, test tubes with a small inverted Durham tube inside, contained 5.0 mL of liquid medium (YNB 6.7 g L^−1^ and different carbohydrates 2%). The tubes were inoculated with 2% of the yeast broth cultures grown for 24 h in YPD broth and incubated in static condition at 28 °C for three weeks. The yeasts’ growth was assessed by CO_2_ gas-formation inside the Durham tube or turbidity observation [9,21]. The test was performed in duplicate.

### 2.6. Sporification Test

Aliquots from yeasts liquid cultures, grown for 24 h in YPD broth, were utilized to inoculate slants of acetate agar medium (sodium acetate 4 g L^−1^; glucose 1 g L^−1^; agar 20 g L^−1^), making a cut on the solid medium. The inoculated slants were incubated for 30 days at room temperature. Spore presence was assessed by light microscope analysis. The test was performed in duplicate.

### 2.7. Growth Rate Determination of Isolated Strains

In order to detect yeast strains ability and rate of growth, 1% inoculated YPD liquid cultures, were incubated at 28 °C for 24 h and optical densities at 600 nm were determined spectrophotometrically at defined time intervals (Varian, Cary mod. 50 BIO UV, Agilent, Technologies, Santa Clara, CA, USA). The test was performed in duplicate.

### 2.8. Micro-Fermentation on Waste Biomass

#### 2.8.1. Substrates Preparation

Biomasses utilized for substrates preparation were composed by of peels, pulp and rotten parts of different seasonal fruit and vegetables to simulate the composition of household wastes.

The substrates compositions were the following:

substrate A: plums peels and pulps (20 g), potatoes peels and pulps (40 g) and distilled water (60 mL)
substrate B: figs peels and pulps (60 g) and distilled water (60 mL).

The different mix of biomasses were crushed, homogenized, placed in 300 mL Erlenmeyer flasks and, after adding deionized water at 1:1 ratio, were sterilized at 121 °C for 15 min.

The dissolved sugar concentrations were determined utilizing the portable refractometer (Mettler Toledo, Milan, Italy).

#### 2.8.2. Micro-Fermentation Assays

Aliquots (3 mL) withdrawn from yeast cultures grown in YPD broth for 24 h at 28 °C were centrifuged at 6000 rpm (2016xgiri) for 5 min. Pellets, washed twice with 3 mL of sterile of phosphate-buffered saline (PBS) solution PBS sterile buffer and resuspended in 3 mL of Ringer solution (were used to inoculate at 2% the flasks containing the different substrates.

Two flasks for each substrate inoculated with the selected native yeast strain were set up. All the flasks were incubated under static condition at 28 °C for 15 days, together with one flask as control (not inoculated medium).

The kinetic of the batch fermentations were determined by measuring the weight loss of the entire set up on the same flask (flask 1) every day [9,10,11,12,13,14,15,16,17,18,19,20,21]. Microbiological and chemical analysis (detection of pH, total titratable acidity (TTA) and VOCs) were carried out on aliquots withdrawn at fixed time intervals (0, 3, 6, 12 and 15 days) from the other flask (flask 2). The micro-fermentation assays were performed in duplicate. These tests were carried out in order to determine the biomass waste mix, that allows native yeast strains to produce the most elevated amounts of high-value aroma compounds.

#### 2.8.3. Microbiological Analyses

The progress of micro-fermentations was checked through determinations of viable yeast cells’ density by plate count. Samples (10 g), collected from each inoculated and uninoculated flasks (flask 2 and control), were placed in sterile Stomacher bags, added with 90 mL of sterile Ringer solution (Thermo-Fisher Scientific, Milan, Italy) and homogenized with Stomacker circulator 400 (Seward, UK), for 1 min at 250 rpm. From serial dilutions, prepared from 1 mL of homogenate, aliquots of 100 µL were plated onto YPD and WL agar Petri dishes (Thermo-Fisher, Scientific, Milan, Italy). After plates incubation at 28 °C for 3–5 days, CFU counts allowed to enumerate the yeast populations during the fermentation processes at fixed interval times [22].

#### 2.8.4. pH and Total Titratable Acidity (TTA) Determination

pH measurement was performed following the AOAC Official Methods [23]. The analysis was performed on 10 g of sample using a pH-meter equipped with a glass electrode (XS, pH 8 + DHS) (Bomarc, Modena, Italy). Measurements were performed in duplicate at all fixed time intervals (0, 3, 6, 12 and 15 days).

The TTA was realized following the AOAC Official Methods [23]. The TTA was determined by titration with a 0.1 M NaOH solution. Ten grams of substrate added with 90 mL of deionized water was stirred for 5 min to obtain a homogeneous suspension. TTA values were obtained from the volumes of 0.1 M NaOH required to reach pH 8.1 ± 0.2. The analyses were performed in duplicate at all fixed time intervals (0, 3, 6, 12 and 15 days).

### 2.9. HS-SPME-GC/MS Analysis of Volatile Components

Headspace volatiles from each sample were analyzed by HS-SPME-GC/MS, using a 7890 Agilent GC system coupled to an Agilent 5975 (Agilent Technologies, Santa Clara, CA, USA) inert quadrupole mass spectrometer equipped with a Gerstel MPS2 autosampler (Gerstel, Mülheim, Germany), as described by Boscaino et al. [10,24] with some modifications. Briefly, 2 g of samples were placed into a 20 mL headspace vial, and 5 μL of 3-octanol (internal standard, 100 mg/L standard solution) were added. The vial was placed in a thermostatic block (40 °C) on a stirrer, the fiber was inserted and maintained in the sample headspace for 30 min, then removed and immediately inserted into the GC/MS injector for the desorption of compounds. The extraction was performed automatically by the multipurpose sampler of the GC/MS system. A silica fiber, coated with 50/30 μm Divinylbenzene/Carboxen/PolyDiMethylSiloxane (DVB/Carboxen/PDMS) SPME fiber (Supelco, Bellefonte, PA, USA) was used for analysis. The operating conditions were as follows: HP-Innowax capillary column (30 m × 0.25 mm ID, film thickness 0.25 μm, Agilent Technologies, Santa Clara, CA, USA) gas carrier was helium (flow 1.5 mL/min), and SPME injections were splitless (straight glass line, 0.75 mm ID) at 240 °C for 5 min, during which time thermal desorption of the analytes from the fiber occurred. The oven parameters were as follows: initial temperature of 40 °C held for 3 min, followed by an increase to 240 °C at a rate of 5 °C/min, and then held for 0 min. The injector, the quadrupole, the source and the transfer line temperature were maintained at 240 °C, 150 °C, 230 °C and 200 °C, respectively. Electron ionization mass spectra in full-scan mode were recorded at 70 eV electron energy in the range 31–400 amu. Volatile organic compounds (VOCs) identification was achieved by comparing mass spectra with the Nist library (NIST 20) and by matching the retention indices (RI) calculated according to the equation of Van Den Dool & Kratz [11,25] and based on a series of alkanes. The data are expressed as relative peak area (RAP) compared to the internal standard. Blank experiments were carried out in two different modalities: blank of the fiber and blank of the empty vial. All the analyses were performed in duplicate for each sample and the results expressed as mean value ± standard deviation.

### 2.10. Molecular Identification of Selected Yeast Isolates

The yeast strains were identified by analyzing the D1/D2 domain of 26S rDNA sequence. The genetic region was PCR-amplified directly from individual yeast colonies following the protocol described by Arroyo-Lopez et al. [12,26]. The standard primers utilized are commonly referred in the literature as NL1 and NL4 [13,27].

### 2.11. Extracellular Enzymatic Activities Determination

Extracellular enzymatic profiles of yeast strains *W. anomalus* YDSCYP4, *P. kluyveri* YDSCYP5 and *S. cerevisiae* YSFWL3 were determined through the semi-quantitative standardized API ZYM micro method system (Bio-Mérieux, Florence, Italy). Following the manufacturer’s instructions, a semi-quantitative screening of 19 different enzyme activities (Alkaline phosphatase, Esterase (4), Esterase Lipase (C14), Lipase, Leucine arylamidase, Valine arylamidase, Cysteine arylamidase, Trypsin, α-chymotrypsin, Acid phosphatase, Naphthol-AS-BI-phosphohydrolase, α-galactosidase, β-galactosidase, β-glucuronidase, α-glucosidase, β-glucosidase, N-acetyl-β-glucosaminidase, α-mannosidase, α-fucosidase) was carried out. All yeast strains were grown overnight in YPD broth at 28 °C. Then, 65 μL were transferred from the yeast suspension into each cupule of the API-ZYM strips and incubated at 37 °C for 4 h. Then, ZYM A (tri-hydroxy-methyl-amino-methane, HCl, and lauryl sulfate) and ZYM B (fast blue BB and 2-methoxy-ethanol) reagents were added to each cupule and all the strips were incubated at room temperature for 5 min, to allow the development of colorimetric reactions. Enzyme profiles of yeast strains were determined by comparison with the Reading Table of API-ZYM kit system.

### 2.12. Micro-Fermentation Assays Under Agitation on Waste Biomass B

Biomass B and the three yeast strains *P. kluyveri* YSDYP5, *W. anomalus* YSDYP4 and *S. cerevisiae* YSFWL3 were utilized for the micro-fermentation assay performed under agitation conditions. For this purpose, biomass B (100 g of peels and fig fruits homogenates) and 100 mL of deionized water were placed in 2 L Erlenmeyer flasks, sterilized at 121 °C for 15 min. and then inoculated (2%) with yeast liquid cultures grown in YPD broth for 24 h at 28 °C. Then, the flasks were incubated in orbital shaker (Eppendorf Inc., Hamburg, Germany) at 28 °C, 150 rpm for 72 h.

### 2.13. Statistical Analysis

The mean and standard deviation were calculated for each experimental parameter. Differences among the experimental samples were determined for each volatile compound by analysis of variance, Duncan’s test, and the results were considered significant if the associated *p* values were below 0.05. To evaluate the relationship between fermentation progression (expressed as weight loss) and the synthesis of volatile compounds for each yeast strain, a covariance analysis (ANCOVA) was performed. Finally, a two-way ANOVA was specifically conducted on the volatile compounds that had shown statistical significance in the ANCOVA models. Statistical data processing was performed using XLSTAT (v 2025). Principal Component Analysis (PCA) was carried out using Tanagra 1.4 software.

## 3. Results and Discussion

### 3.1. Spontaneous Alcoholic Fermentation of Wastes—Weight Loss

Fermentations were monitored through determinations of flasks’ weight loss and microbiological analyses. Measurements of weight losses were carried out along the entire micro-fermentation experiments until any weight change was recorded for three sequential days. With biomass “a” and “b” the constant weight loss recorded along the entire spontaneous fermentation, that led to a reduction of 17.5 and 25 g of the flasks weight after 30 days respectively, was significant of a bio-process that proceeded without any interruption. Differently for biomass “c”, any weight loss was detected. These data clearly showed that the native microflora in waste biomasses ‘a’ and ‘b’ was able to trigger and carry out alcoholic fermentation, whereas in ‘c’, it was unable to initiate this biological process. Moreover, weight loss provides an indirect evaluation of fermentative vigor; it shows the conversion rate of fermentable sugars into ethanol and carbon dioxide. The fermentative vigor is a very important technological feature to be evaluated during the selection of yeast strains [14,28,29].

### 3.2. Microbial Analysis of Yeast Populations During Spontaneous Fermentation of the Different Waste Biomasses

**Figure 2:**
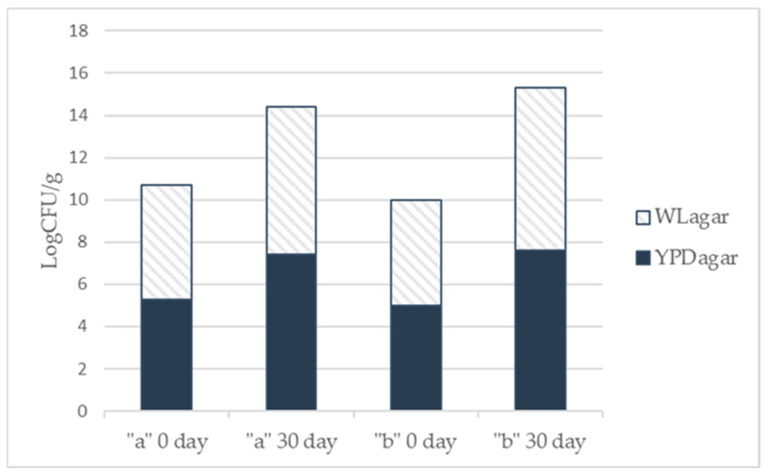
Enumeration of native yeast populations loads during the waste spontaneous fermentation processes, carried out during 30 days at 28 °C.

Data obtained from the microbiological analysis of spontaneous fermentation processes carried out by the native yeast populations on waste biomasses “a” and “b”, at different times (after 0, 5, 10, 15, 20, 25 and 30 days), gave the similar results. In Figure 2, the data of the loads of yeast populations were reported, expressed as Log CFU/g, at time 0 (start) and time 30 days (end of process). In detail, for waste biomass “a” on YPD and WL agar plates the yeast loads grew of about two logarithmic orders, 5.3 to 7.4 Log CFU/g and 5.4 to 7 Log CFU/g respectively, during the spontaneous fermentation process. While, for waste biomass “b” the yeast loads detected on YPD and WL agar were slightly more than two logarithmic orders, in particular, 5 to 7.6 Log CFU/g on YPD and 5 to 7.7 Log CFU/g on WLagar, along the thirty days. These data underline that the waste figs (biomass “b”) contained higher lot of sugars but also a major amount of fermentative yeasts population respect at biomass “a”.

Unfortunately, on substrate “c” any yeast colony was detected on the two media plates along the fermentation. This lack to start fermentation could be caused by the origin of the strawberry grapes because they were bought in a food shop. In particular, the packages at the end of shelf-lifetime recommended, and the bunches of grapes that showed several damaged and rotten berries were chosen. On the other hand, it is known that at industrial level the fruits before the packaging were treated with antimicrobial and antifungal additives to kill all microbial components, and to preserve the fruits as bunches of grapes particularly sensible to mold proliferations [30]. Probably, then the residual yeast flora on these berries was destroyed or was not able to grow.

For yeasts isolation, five colonies from each plate, that showed different shape, appearance and color were picked up in a random way and streaked for purification on plates with the same composition. The isolated yeast strains were finally transferred on slants and stored in the ISA-CNR strain library. These microbiological data highlighted that fruit and vegetable wastes used as substrates for spontaneous fermentation processes, could be a potential source of new autochthonous yeast strains. Furthermore, from the native microflora of these agro-food waste (AFW), yeast strains with interesting and innovative technological features, could be isolated and characterized for subsequent utilizations as substitute or support of the most well-known strains and species in biotechnological processes for the production of new added value biomolecules or fermented products at industrial levels [15,16,17,18,19,20,21,22,23,24,25,26,27,28,29,30,31]. Moreover, these wastes could also be used as such or modified as growth media for the production of yeast biomass, since they are microelements sources and contain various assimilable nutrients [9,17,18,20,32].

### 3.3. Morphological and Physiological Characterization of Yeast Strains

In Table 1 are reported the results obtained from the analysis of the different morphological and physiological features of the native strains isolated from waste “a” and “b”. With respect to the appearance, shape and color of colonies, the results highlighted that many of them showed smoot surfaces umbonate shape and only some of them presented jagged edges; finally, the colors were white and cream principally. Then, 10 colonies that gave pure cultures in liquid YPD broth, as detected by phase-contrast optical microscopy analysis, were chosen for the subsequent experiments.

#### 3.3.1. Assimilation of Carbohydrate Sources

This test determined the ability of the isolated yeast strains to grow on particular carbon compounds supplied individually as the sole source of energy. In detail, all the strains showed the ability to quickly ferment glucose (Gl), fructose (Fr) and sucrose (Su). Maltose (Ma), instead, as reported in Table 1, was fermented only by YDSCYP4, not utilized by 5 strains, and assimilated but not fermented (CO_2_ absence in Durham tubs) by YDSCYP1, YSFWL1, YSFWL2 and YSFWL3 [21].

#### 3.3.2. Sporulation Test

The results obtained are reported in Table 1. The observation by phase contrast microscope of the yeast cultures grown on agar acetate medium after 30 days revealed the presence of spores in two strains isolated from waste “a” YDSYP1 and YDSYP3 and four from waste medium “b” YSFWL1, YSFWL3, YSFWL4 and YSFWL5. Spore-forming capacity is an important feature that allows yeast strains to withstand several adverse conditions related to chemical-physical parameters (temperature, pH, osmolarity) nutrient starvation surviving in a resistant spore form [19,33].

#### 3.3.3. Molecular Identification of Yeast Strains

The identification of the isolated 10 yeast strains at species level was possible due to the elevated identity levels (≥99%) of the PCR-amplified sequences of 26S rDNA D1 and D2 domains with those present in the NCBI database. It is worth noting that all the strains isolated from biomass “b” (figs and grape waste) belonged to *S. cerevisiae* due to the high fermentable sugars content of the biomass. It is not surprising in consideration of the more relevant rates of sugar uptake and the more elevated tolerance to considerable sugar concentrations of *Saccharomyces* strains respect to non-*Saccharomyces* spp. [9,20,34,35]. Non-*Saccharomyces* yeasts were instead isolated from biomass “a” (kiwi, apple and potatoes residues) with the predominance of *Pichia* and *Wickerhamomyces* spp. Also, Goncalves et al. [21,36] reported the prevalence of *Pichia* species with a good representation of *P. kluyveri* strains in their studies on native yeasts microflora from fruit and vegetable wastes with culture dependent approach. As reported in Table 2, the aroma producer *W. anomalus* (formerly *P. anomala*) [22,37] was also isolated and its presence in AFW was already reported by the previously cited Goncalves studies.

### 3.4. Yeast Growth in Liquid Medium

For each isolated strain, the capacity to grow in liquid culture and produce microbial biomass was assessed utilizing YPD broth as substrate. On the basis of the spectrophotometrically measured absorbance at 600 nm, reported as optical density (O.D.), only seven strains that showed O.D. equal or higher than 0.7 (chosen as reference value) were selected for further experiments since they were considered to be endowed with an appreciable capability to produce vital biomass under culturing conditions. These strains comprised *W. anomalus* YDSCYP1 and YDSCYP4, *P. kluyveri* YDSCYP5 and *S. cerevisiae* YSFWL1, YSFWL2, YSFWL3 and YSFWL5 that showed O.D. of 1.26, 0.70, 0.8, 1.54, 2.4, and 2.10 respectively [28,29].

### 3.5. Fermentation Kinetic of Yeast Strains Grown on Biomass A and B

The seven isolates previously reported were utilized as inocula in fermentation process for aromatic compounds production utilizing waste biomasses as substrates. The fermentation abilities of the seven strains were determined by measuring weight losses of the entire set up (Erlenmeyer flask and liquid growth culture), at defined times (after 3, 6, 12 and 15 days) [28]. The data of weight losses were shown by a Parwise plot (Figure 1). Real values were obtained by subtracting the weight losses of uninoculated samples (reported in figures as Controls) due to evaporation, that in our experiments reached about 2 g at the end of the process. Fermentation kinetics of yeast strains isolated from fruit and vegetable biomass waste “a” (*W. anomalus* YDSCYP1 and YDSCYP4 and *P. kluyveri* YDSCYP5) and from figs and grape biomass waste “b” (*S. cerevisiae* YSFWL1, YSFWL2, YSFWL3, YSFWL5) were determined utilizing two different biomasses as substrates, namely biomass A (50% distilled water, 30% and 20% plums and potatoes biomasses) and biomass B (50% distilled water and 50% figs biomasses). The analysis of weight losses produced by *W. anomalus* YDSCYP1 and YDSCYP4 and *P. kluyveri* YDSCYP5 strains utilizing biomass A as substrate, revealed that *W. anomalus* YDSCYP4 showed the fastest adaptation capacity to the process conditions of batch micro-fermentation conducted in static mode at 28 °C. This strain produced the highest weight loss (3.73 g) after only 3 days and retained this fermentative capacity along the entire process giving a weight reduction of 9.49 g after 15 days. The other strains *W. anomalus* YDSCYP1, and *P. kluyveri* YDSCYP5 produced on average value of weight loss of 1.61 g after 72 h of incubation and this fermentation rate was maintained along 15 days of the process, giving an average value of 3.87 g of weight reduction for the two strains.

**Figure 3:**
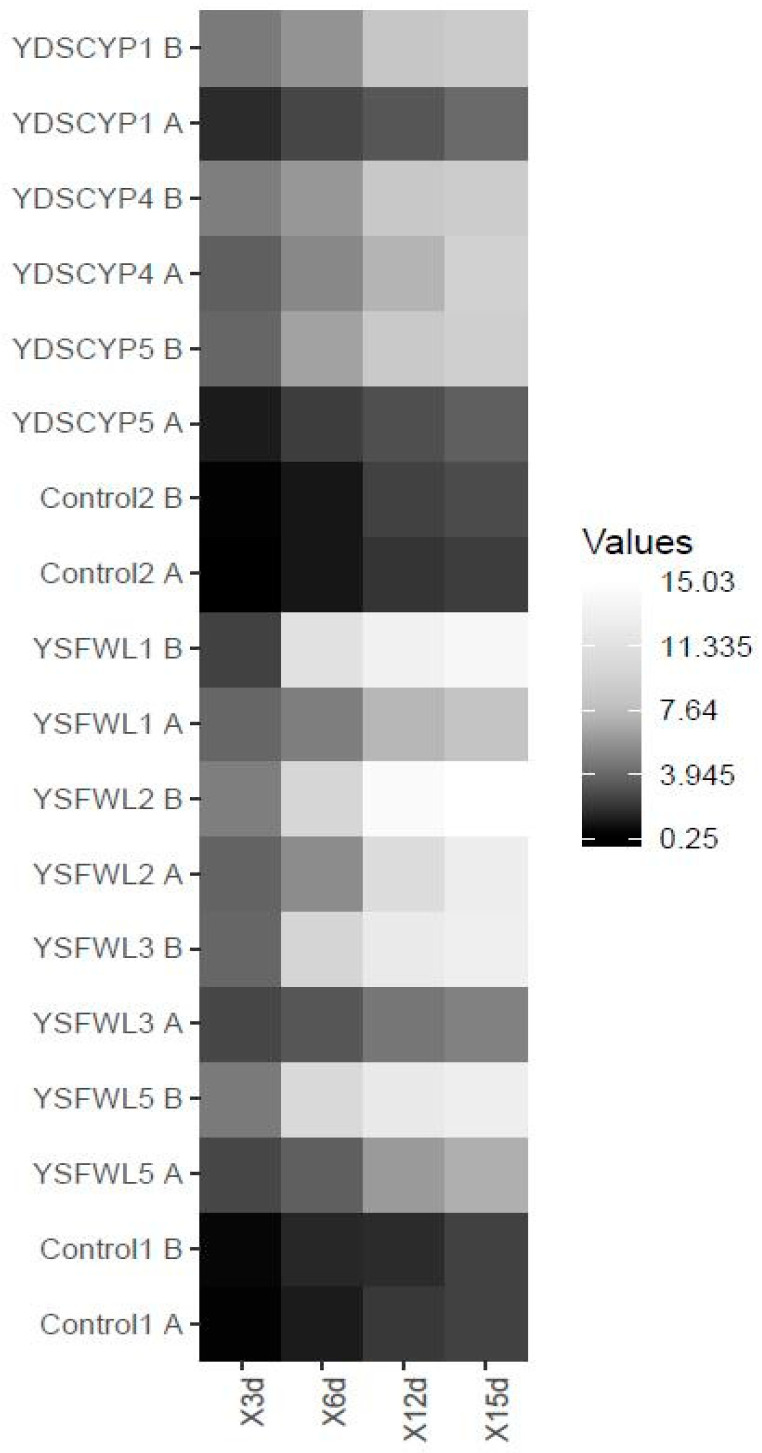
Parwise plot of weight loss.

The strain *W. anomalus* YDSCYP4 showed this capacity of fast adaptation also with biomass B, as shown in Figure 3. With biomass B also *W. anomalus* YDSCYP1 and *P. kluyveri* YDSCYP5 demonstrated after 15 days similar weight losses (8.88 and 9.26 g respectively) to *W. anomalus* YDSCYP4 (8.99 g). Specifically, *P. kluyveri* YDSCYP5 reached after 6 days values of weight losses slightly higher than that of *W. anomalus* YDSCYP4, and this trend was maintained until the end of the process when reached similar values to those of *W. anomalus* YDSCYP4. Conclusively it is worth noting that non-*Saccharomyces* strains showed a more vigorous fermentative activity when biomass B was utilized as substrate. This was demonstrated by average weight losses (9.04 g) higher in respect to that of 5.75 g measured with biomass A [15,16,17,18,19,20].

As regarding yeasts isolated from figs waste (*S. cerevisiae* YSFWL1, YSFWL2, YSFWL3, YSFWL5), not all the strains were able to grow on biomass A. Only the strains YSFWL1 e and YSFWL2 activated their metabolic activity within the initial three days, producing increasing weight losses along the entire process. YSFWL2 strain showed a more pronounced fermentative activity that lowered weight by 53, 9% more respect to YSFWL1 reaching a weight loss of 12.7 g after 15 days of process. YSFWL3 and YSFWL5 activated the fermentative metabolism only after a week that represents too long period that exposes to contamination risk. Moreover, also after 15 days lower levels of metabolic activity were achieved as indicated by a maximum weight loss of 7.05 g for YSFWL5, as suggest [29,30,31,32].

Differently, all the strains were able to utilize biomass B switching on their fermentative metabolism in the first three days as demonstrated by the comparison of weight losses with those of controls; moreover, they continued to reduce the weight along the entire process. Again, *S. cerevisiae* YSFWL1 showed a good capacity to convert fermentable sugars to ethanol and CO_2_, followed by *S. cerevisiae* YSFWL2 mainly starting from the 12th day of the process. In parallel, *S. cerevisiae* YSFWL3 and YSFWL5 strains showed a similar fermentation rate despite they produced lower weight losses especially after 15 days.

In summary, the most marked weight losses after 15 days of processing were produced when biomass B was utilized as a substrate, with mid values of 9.04 and 9.38 g for YDSCYPx and YSFWLx strains respectively, that were sensibly higher than 5.75 and 8.25 g obtained by YDSCYPx and YSFWLx, respectively, when biomass A was utilized. Moreover, from the above reported data, was evident that the strains isolated from figs waste (YSFWL1, YSFWL2, YSFWL3, YSFWL5) exhibited the best ability to utilize the fermentable sugars with both substrates (biomass A and B), and the best results were achieved with biomass B to which they were better adapted; this was highlighted by a mid-value of weight loss after 15 days about 14% higher [9,10,11,12,13,14,15,16,17,18,19,20,21,22,23,24,25,26,27,28]. The most pronounced weight reductions was measured for *S. cerevisiae* YSFWL1 followed by YSFWL2 strain on both biomass A and B, this was already evident from the initial three days of process and increased along the time. *S. cerevisiae* YSFWL3 and YSFWL5, instead, showed the slowest rate of fermentable sugars utilization as highlighted by significant weight losses only between 6th and 12th day and a limited value (7.27 g) at the end of the process [29].

### 3.6. Biomass Yield from Batch Fermentations Utilizing Biomass A and B as Substrate

**Figure 4:**
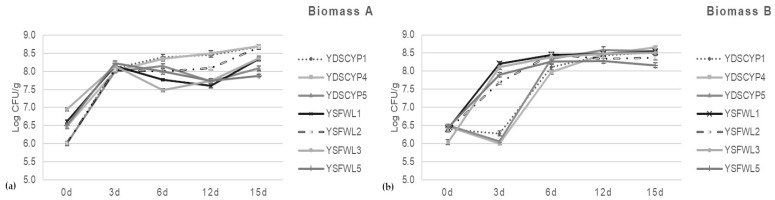
In panel (**a**) vital cell loads reached by YDSCYPx and YSFWLx native yeast strains during fermentation on Biomass A were reported. In panel (**b**) vital cell loads on Biomass B were showed.

Figure 4a,b reported microbial counts (Log CFU/g) from batch micro-fermentation experiments. Figure 4a shows the data when utilizing biomass A as a carbon source and YDSCYPx and YSFWLx strains as inocula. When YDSCYPx yeasts, isolated from fruit and vegetable waste, were utilized, the data showed that all the strains were able to quickly adapt and carry out the fermentation process. In particular, *W. anomalus* YDSCYP4 and YDSCYP1 produced the highest vital cell count of 8.70 and 8.68 (Log CFU/g), respectively, after 15 days remaining in exponential phase.

Also, *S. cerevisiae* YSFWL1, YSFWL2, YSFWL3 and YSFWL5 strains were able to survive and grow on biomass A and the highest vital count was recorded for *S. cerevisiae* YSFWL2 after 15 days (8.67 Log CFU/g) while *S. cerevisiae* YSFWL5 produced the least numerous population that accounted for 7.88 (Log CFU/g). Moreover, growth curves profiles showed that *S. cerevisiae* YSFWL2 gave better performances than YSFWL1 and YSFWL3. Figure 4b reported the growth kinetics of the same yeast strains cultured utilizing biomass B, showed that the non-*Saccharomyces* strains YDSCYPx showed more difficulty in the adaptation, reaching lower vital counts in the first to 3 days respect to biomass A. After the growth rate was accelerated, giving higher vital loads that accounted to 8.51, 8.55, 8.65 (Log CFU/g) for *W. anomalus* YDSCPY1, *P. kluyveri* YDSCYP5 and *W. anomalus* YDSCYP4 respectively. Moreover after 15 days the growth curve of *W. anomalus* YDSCPY1 e *P kluyveri* YDSCYP5 reached the plateau while that of *W. anomalus* YDSCPY4 remained in exponential phase as when biomass A was utilized. On the other hand, all the strains of autochthonous *S. cerevisiae*, inoculated in biomass B, showed a quicker growth rate and reached the stationary phase already after 12 days. YSFWL2 e YSFWL5 reached the highest microbial load already after 6 days (8.46 and 8.26 Log CFU/g respectively), that slightly lowered up to 15 days. Differently, YSFWL1 and YSFWL3 strains achieved the highest vital cell counts at 15 days (8.53 and 8.51 Log CFU respectively) showing values comparable to those of YDSCYPx strains.

Biomass B, (figs waste), being a substrate richer in fermentable sugars than biomass A (15% and 6% dissolved sugars respectively), allowed a faster adaptation of the *Saccharomyces* strains YSFWLx and the achievement of higher biomass yields in particular for the strains *P. kluyveri* YDSCYP5 and *S. cerevisiae* YSFWL3 [16,18,23,31,32]. In accordance with experimental data concerning adaptation to different chemical and physical conditions, micro-macronutrients availability and temperature and considerations about fermentative abilities and speed of process activation, together with the similarity of the microscope images of ascospores with those reported by Sidari et al. [19,33], it is confirmed that YSFWLx strains belong to the *Saccharomyces* genus [8,14,15,16,17,18,19,20,21,22,23,24,28,34].

### 3.7. Batch Fermentations pH

The obtained data evidenced that YDSCYPx strains were able to slightly modify the pH of culture medium when both biomass A and B were utilized. With biomass A, all the strains modify pH in the range 3.9–4.7 showing a similar behavior in the first 72 h and along the entire bio-process. In particular, for *W. anomalus* YDSCYP1 and YDSCYP4 after three days were measured low reductions in pH that came back to the initial values after six days and rise at the end of the process. *P. kluyveri* YDSCYP5, instead, did not modify the pH of culture medium at three days but give a lower value at 15 days. This is also confirmed by the volatile organic acids profiles detected (Table 2).

With biomass B, *W. anomalus* YDSCYP1 and YDSCYP4 and *P. kluyveri* YDSCYP5 lowered the pH values of culture medium in the time interval between third and sixth day of process and successively a pH rise was measured until the end of the process. This is in accordance with the amount of volatile acids compounds determined by SPME-GC/MS (Table 3) Also *S. cerevisiae* YSFWLx strains had a limited capacity to modify starting pH and with both biomass A and B and the variations were in the range 0.3–0.6.

The *S. cerevisiae* YSFWLx strains in biomass A, showed a pH reduction along the entire fermentation process with the only exception of YSFWL2 that lowered the medium pH after 72 h that increased on the 15th day. With biomass B, instead, all the strains decreased pH culture medium. This behavior was confirmed by the data of the volatile organic acids production as reported in Table 2 and Table 3. In brief slight pH variations in all the fermentation processes were measured and as reported in the literature, this is strictly linked to the kind of microorganism utilized. As highlighted by several authors, the metabolic activities of yeasts, differently from lactic bacteria, did not produce acidic compounds able to change the chemical-physical and technological features of the culture medium [14,15,16,17,18,19,20,21,22,23,24,25,28,29]. Yeast produces only limited amounts of compounds such as aldehydes, esters, glycerol, succinic and other organic acids, that have a limited capacity to modify pH [9,10,26,27].

### 3.8. Production of Volatile Organic Compounds

#### 3.8.1. Analysis of VOCs Produced with Biomass A as Substrate

The analysis of volatile organic compounds (VOCs) produced during batch micro-fermentation experiments utilizing biomass A as carbon and energy sources allowed to detect a maximum of 27 volatile compounds (Table 2) belonging to the following classes: aldehydes (3), ketones (1), esters and acetates (9), alcohols (7), acids (5) and terpenoids (2). alcohols, esters and acetates classes were the best represented VOCs.

Table 2 reports the amounts, expressed as RAP ± sd (Relative Area Peaks ± standard deviation), of VOCs produced by different native yeast strains when biomass A was utilized as substrate. In these batch fermentation experiments, a limited production of these volatile compounds by all the strains were measured.

Esters, acetates and alcohols were among the best represented classes of volatile compounds produced along the fermentation experiments.

Isoamyl alcohol and ethanol were produced at the highest levels among the alcohol compounds. The strains YSFWL1, YSFWL2, YSFWL3 and YSFWL5 (*S. cerevisiae*), isolated from figs waste, when utilized as single inoculum, generated fair amounts of isoamyl alcohol whose values increased with time. The highest amount was detected for YSFWL1 after 15 days from the inoculum as indicated by a RAP of 1572.23 ± 1.45. For the other strains comparable quantities were measured (1194.05 ± 22.09, 1202.35 ± 21.62 and 1231.88 ± 34.15, for YSFWL2, YSFWL3 and YSFWL5 respectively). Isoamyl acetate amounts, however, were not similarly elevated also after 15 days, probably as suggested by Yan et al. [28,38], due to a limited alcohol acetyl transferase activity and/or availability of acetylCoA. It is worth noting that, looking at the weight loss profile and growth curves (Figure 3 and Figure 4), all the above mentioned strains were scarcely active at metabolic level during batch micro-fermentation experiments. Moreover, since all the strains were in the exponential phase of growth, in the time interval 12–15 days, acetylCoA utilization for cell biomass production, limits its availability for ester acetate production through trans-acetylation reactions.

Concerning native strains isolated from fruits and vegetables waste (*W. anomalus* YDSCYP1 and YDSCYP4 and *P. kluyveri* YDSCYP5), they did not give a relevant contribution to isoamyl alcohol and isoamyl acetate production with the exception of *P. kluyveri* YDSCYP5 that produced isoamyl acetate (1014.12 ± 19.31) and ethyl acetate (1465.48 ± 23.09) predominantly after 3 and 15 days of fermentation respectively.

Moreover, also YDSCYP4 after 72 h gave good levels of ethyl acetate (2851.56 ± 191.27) that decreased after to 15 days to negligible amounts. This correlates with a higher growth rate for this strain at 15 days of process when, as reported in Figure 4a, the microorganism culture was at the exponential phase of growth. *W. anomalus* ability of enhancing different aroma is in accordance with several authors that reported fair increases in fruity acetate esters in wine-like media often highlighting ethyl acetate yields of above 150 mg/L [39,40,41]. Moreover, the outperformances of *W. anomalus* vs. *Saccharomyces* strains with respect to aroma molecules production on low-sugar biomass A via a stress-adapted metabolism, is highly consistent with biorefining reports [42].

Also concerning ethanol production, *S. cerevisiae* YSFWL1, YSFWL2, YSFWL3 and YSFWL5 gave higher contributions than *W. anomalus* YDSCYP1 and YDSCYP4 and *P. kluyveri* YDSCYP5 and the most significant amounts of ethyl alcohol derived from YSFWL1 after 15 days of fermentation (4279.28 ± 28.05). This is in agreement with data about weight loss that were higher for yeast strains isolated from figs (mid values of 8.25 g and 5.75 g over 15 days of process for native yeasts YSFWLx and YDSCYPx respectively). Ethanol amounts increased during time for all the YSFWLx strains with the exception of YSFWL2 that reduced its alcohol production by 34.19% after 15 days of fermentation process. As concerning *W. anomalus* YDSCYP1 and YDSCYP4 and *P. kluyveri* YDSCYP5 strains, the obtained ethanol amounts were sensibly lower (mid value 940.94 after 3 days and were reduced to zero after 15 days of fermentation except for YDSCYP5 that yielded 87.5% alcohol of that measured after 3 days (912.09 ± 16.13 and 798.16 ± 27.80 after 3 and 15 days of fermentation). This is in complete agreement with the widely documented low capacity of alcohols production by non-*Saccharomyces* strains [29,35,37,38] conversely to their high yields of compounds such as acetate esters. In particular, *W. anomalus* is reported as a better ester producer respect to *Saccharomyces* strains [30,43,44,45] together with *P. kluyveri* that can strongly boost banana-like and fruity esters. This is confirmed also by a large screening among 99 non-*Saccharomyces* strains carried out by Gutierrez et al. [46] that identified a *P. kluyveri* strain as exceptional producer of strong, pleasant banana aroma rather than solvent-like notes, among the *Pichia* spp. [31,47,48,49].

Differently from the alcohol and ester acetate compounds the other volatile molecules were produced in more limited amounts as evidenced in Table 2.

As concerning aldehyde compounds after 15 days, the production of 2-methylpropanal was observed in batch fermentations inoculated with *S. cerevisiae* YSFWL1, YSFWL3 e YSFWL5 (56.65 ± 1.30, 92.53 ± 0.26, 80.76 ± 1.75 respectively) together with 2-methylbutanal only by YSFWL5 (95.55 ± 2.04). While these compounds were absent in the uninoculated controls and thus liberated by the fermentative activities, conversely furfural aldehyde (7.75 ± 0.07) was already present in the control at time zero. Acetoin volatile compound was produced after 15 days in batch cultivations inoculated with *S. cerevisiae* YSFWL1, YSFWL2 e YSFWL5 (15.51 ± 0.24, 14.69 ± 0.15, 6.65 ± 0.21 respectively). Acetic acid, the best represented compound among acids, was mainly produced by YSFWL2 (218.27 ± 15.52) after 72 h. Finally β-myrcene and limonene (mid value 1.24 and 6.02 respectively), present among terpenoids, were characteristic of potatoes and plums waste according to Qin et al. [32,50] and Chen et al. [33,51].

The ANCOVA analysis revealed a positive linear relationship between fermentation progression (expressed as weight loss) and the synthesis of various volatiles (see Table S1 in Supplementary Materials). Among the compounds monitored, those that showed the most marked response—defined by statistical significance of *p* < 0.05 and a β slope coefficient > 4.4—were ethyl acetate for yeasts YDSCYP1 (3d), YDSCYP4 (3d) e YDSCYP5 (15d); isoamyl acetate for YSFWL1 (15d); and ethanol for YSFWL1 (3d and 15d), YSFWL2 (3d), YSFWL3 (15d) and YSFWL5 (15d). In particular, ethyl acetate showed a high slope (β > 14) in YDSCYP 1 and YDSCYP4 yeasts after only 3 days of fermentation. This extremely significant value suggests that these strains are highly efficient in the synthesis of acetate esters in the early stages, being able to drastically modify the aromatic profile of the substrate within the first 72 h of the process.

In order to better understand the differences among the VOCs profiles of the native yeast strains during fermentation on biomass A, a principal component analysis (PCA) of the volatile compounds was performed.

**Figure 5:**
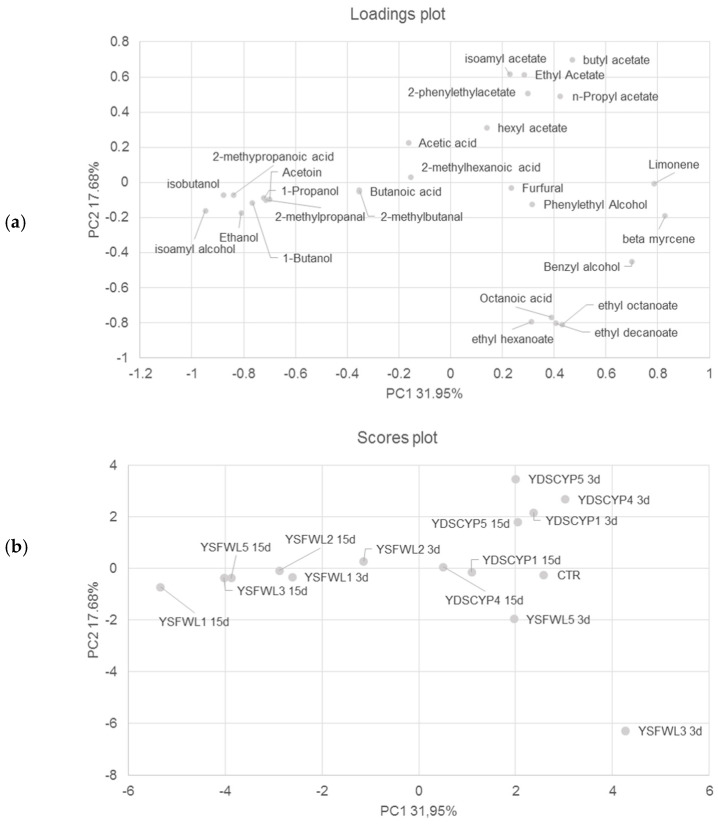
PCA analysis of the volatile compound. Panel (**a**) Loading plot. Panel (**b**) Score plot of VOCs detected with Biomass A.

Scores plot of the samples are shown in Figure 5b, and the corresponding loadings, establishing the relative importance of the variables, are shown in Figure 5a.

The first two principal components (PCs) explained about 49.63% of the total variance of the data. As determined by the two PCs (factors), samples were located in two different clusters. In detail, it was possible to point out two groups comprising most native yeast fermentative strains. In particular, as shown by the scores plot Figure 5b, the first group comprised *W. anomalus* YDSCYP1 and YDSCYP4 and *P. kluyveri* YDSCYP5 after 3 and 15 days of fermentation, YSFWL5 (3d) and the control substrate, and were characterized principally by acetates and terpenes. In the second group, there were YSFWL1 (3d and 15d) and the strains *S. cerevisiae* YSFWL2, YSFWL3, YSFWL5 (15d) that were characterized principally by the production of alcohols such as isoamyl alcohol. Finally, *S. cerevisiae* YSFWL3 (3d) was localized in the IV quadrant of the scatterplot and was characterized by ester compounds.

#### 3.8.2. Analysis of VOCs Produced with Biomass B as Substrate

In Table 3 were reported the amounts of different VOCs expressed as (RAP ± sd), obtained from batch fermentations utilizing biomass B as substrate and the single strains *S. cerevisiae* YSFWL1, YSFWL2, YSFWL3 and YSFWL5, *W. anomalus* YDSCYP1 and YDSCYP4 and *P. kluyveri* YDSCYP5 as inocula. Analysis of VOCs produced allowed to point out 25 compounds belonging to the classes of aldehydes (3), ketones (1), esters and acetates (10), alcohols (6), acids (3) and terpenoids (2). Aldehydes were revealed in the control sample at zero day (0d) (15.22 ± 1.15), and only 2-methylpropanal that was produced by *S. cerevisiae* YSFWL3 after 15 days, increased from 7.43 ± 0.68 (0 day) to 22.75 ± 1.01 (15 days). This class of compounds was not revealed in any other fermented sample. For ketones was pointed out only acetoin that was already present in the control sample at 0 day. In the fermented substrate acetoin increased from 3 days to 15 days when *S. cerevisiae* YSFWL1 (3d 4.56± 0.25 to 15d 118.19 ± 0.79) and *S. cerevisiae* YSFWL3 (3d 5.79 ± 0.21 to 15d 60.78 ± 3.6) conducted the process. Moreover, was already present at 3d when *S. cerevisiae* YSFWL5 (83.02 ± 0.26) was inoculated and remained unchanged up to 15d (88.07 ± 2.15).

Also, utilizing biomass B as substrate, native yeast strains produced volatile compounds mainly belonging to esters and acetates class among which the most represented were ethyl acetate and isoamyl acetate. In particular, ethyl acetate increased in batch fermentations inoculated with *S. cerevisiae* YSFWL1, YSFWL2 and YSFWL5 already after 3 days and also until 15 days only for YSFWL1 and YSFWL5, for YSFWL2, instead, a reduction was detected. When YSFWL3 was utilized ethyl acetate lowered drastically after 3 days and increased after 15 reaching lower values than those detected for the other strains. YDSCYPx strains showed an ethyl acetate production that increased up to 72 h especially by YDSCYP1 and YDSCYP4 then decreasing after 15 days. YDSCYP5, instead, gave a less evident increase after 72 h reaching a constant amount up to 15 days. *S. cerevisiae* YSFWLx strains produced increasing amounts of isoamyl acetate up to 72 h that decreased except for YSFWL5 that did not produce this acetate ester. YSDCYPx strains gave isoamyl acetate up to 3rd day and its quantity decreased at the end of the micro-fermentation with the sole exception of YDSCYP5 that allowed to achieve the highest yield up to 72 h that remained constant up to the 15th day.

Among alcohols the best represented were ethanol and isoamyl alcohols. While all YSFWLx strains were able to increase ethanol yields, YDSCYPx strains produced ethanol up to 3rd day. Subsequently *W. anomalus* YDSCYP1 and YDSCYP4 strains drastically reduced the yields up 15th day whereas YDSCYP5 retained it constant until the end of the bio-process.

Isoamyl alcohol production increased in all batch inoculated with YSFWLx, on the contrary for YDSCYPx strains only *W. anomalus* YDSCYP1 and YDSCYP4 showed a weak capacity of production up to 72 h that decreased until the end of the process.

Acids were represented mainly by acetic acid and YSFWL2 produced the highest amount at 3rd day (172.40 ± 14.59) together with *W. anomalus* YSDCYP1 and YSDCYP4 (313.24 ± 0.03 and 249.83 ± 8.78 respectively).

Finally, among terpenoids β-pinene and limonene (mid value 0.63 and 1.41 respectively) characteristics of figs biomass were detected [34,35,52,53,54].

The ANCOVA analysis revealed a positive linear relationship between fermentation progression (expressed as weight loss) and the synthesis of various volatiles (Table S2, in Supplementary Materials). In particular, the compounds that showed statistical significance of *p* < 0.05 and a coefficient β > 4.4 were ethyl acetate for yeasts YDSCYP1 (3d), YDSCYP4 (3d) and YDSCYP5 (3d and 15d); isoamyl acetate for YDSCYP5 (3d and 15d), isoamyl alcohol for all yeasts; and ethanol for YSFWL1 (15d), YSFWL2 (15d), YSFWL5 (15d), YDSCYP1 (3d) and YDSCYP4 3d, in the production of ethyl acetate (β > 23) by the YDSCYP1 and YDSCYP4 yeasts was also observed in substrate B after only three days of fermentation. This increase was significantly higher than that observed in substrate A, suggesting that the specific composition of substrate B acts as a metabolic stimulus, enhancing the ability of these yeasts to synthesize this ester in the early stages of the process.

As it is clear from the data reported in Table 3, non-*Saccharomyces* strains of the YDSCYPx group were better producers of acetate esters in respect to the *Saccharomyces* ones. Moreover, they liberated the highest amount of ethyl and isoamyl acetate esters after 72 h of fermentation when they showed the lowest vital cell counts (Figure 4b). *P. kluyveri* YDSCYP5 gave the best yields of isoamyl acetate, which remained constant throughout the fermentation process. This strain also achieved similar yields and a similar production pattern for ethyl acetate over time. These central findings are consistent with the existing literature reporting a greater ester production capacity for *W. anomalus* and *P. kluyveri* compared to *Saccharomyces* strains [52]. The authors found that the strain *P. kluyveri* PkY2 isolated from naturally fermented fruit juice was able to produce 5.66 fold more esters than the control (*S. cerevisiae*) in Yinhong fruit wine obtained through fermentation of plum juice. Also, ref. [37] verified that inoculation with *P. kluyveri* LPBIIA resulted in a 2- to 6-fold increase in the concentration of isoamyl acetate in the obtained brandy compared to commercial products. Several authors [55], van Wyk and coworkers [49] have reported that the presence of elevated levels of alcohol acetyl transferase activity in non-*Saccharomyces* strains and particularly in *P. kluyveri* and a more effective metabolic acetyl CoA generation through respiratory or specialized oxidative pathways, are the basis of the enhanced acetate ester formation [56]. In this context, Wei and coworkers [57] had a central role in identifying, through transcriptomics and exometabolomic methodologies, the key genes and enzymes linked to the overproduction of the aroma-active higher alcohols and esters during fermentation of apple juice by *P. kluyveri*. In the first four days of fermentation when the highest concentrations of aroma-active compounds including acetate esters and fusel alcohols were detected, sensibly enhanced expression was evidenced not only for genes coding for enzymes involved in all the steps of the Ehrlich pathway (aminotransferases, decarboxilases, alcohol and aldehyde dehydrogenases) and ester synthesis (acetyl and acyl transferases) but also for the genes responsible for the synthesis of branched aliphatic and aromatic amino acids which are the substrates for alcohol formation. Moreover, duplication events of specific genes coding for ADH (alcohol dehydrogenases) and LEU4 (enzyme involved in leucine synthesis) highlighted the genomic potential of *P. kluyveri* responsible of the higher evolved capacity of non-Saccharomyces yeasts respect to *S. cerevisiae*, of enhancing the aroma complexity of several products. This determined the well-established practice of utilizing non-*Saccharomyces* strains, such as *W. anomalus* and *P. kluyveri*, in starter cultures for enhancing the floral and fruity aromas of various fermented foods, including chocolate [58], as well wine [48,59], beer [60] and kombucha [49], among others. Finally, these data emphasized the crucial impact of substrate composition on the yield and diversity of aroma compounds, as evidenced by the significantly lower quantities of flavor molecules produced when biomass A was utilized with all the inoculated strains. This finding is supported by several studies, including those reported by [37], which demonstrated high volatile productivities only when spent grain wort was added to grape juice. Consequently, the exceptional performance observed with fig waste (biomass B) may be due to its high fermentable sugar content, favorable amino acid profile and the metabolic capability of the YDSCYPx strains.

These promising findings encourage the discussion of potential applicability of the isolated autochthonous strains to produce aroma-active molecules in industrial biorefinery applications. However, particular attention has to be paid to several aspects regarding substrate, strains and specific operational constraints that affect the industrial scalability of the results. The wide heterogeneity at strain level within the *W. anomalus* and *P. kluyveri* spp. implies that the elevated performances of YDSCYP4 and YDSCYP5 may not be universal traits of these species. The specific metabolic characteristics of these isolates could be the results of the isolation procedures that may have selected for yeasts adapted to these specific substrates compositions and this potentially determined their performance on biomass B. However, this substrate-matched strain selection limits the predictive value for biorefining applications on different AFW of these isolates. Moreover, the absence of axenic culture conditions in AFW biomasses utilized in industrial fermentation poses serious concerns due to the prohibitive costs for ensuring eventual sterile conditions. Consequently, strains validations also respect competitive vulnerability against possible native *Saccharomyces* contaminants of the substrates need to be tested. These studies with a pure-culture approach, albeit scientifically advantageous, could have overestimate these strains performances in industrial settings. The aroma dependence of NCY performance on substrate composition, combined with the heterogeneous composition of agri-food waste, which also varies seasonally, requires the implementation of standardized pre-treatment protocols to ensure consistent performance across different agricultural ecosystems. Another challenge is the low ethanol-producing capacity of NCY compared to *Saccharomyces* strains. Consequently, incomplete utilization of sugars poses significant economic constraints for cost-effective, integrated biorefinery processes. Studies on mixed fermentation strategies utilizing the sequential inoculation of non-*Saccharomyces* strains as initial starters, followed by *S. cerevisiae* strains, could address the issue of lower ethanol yields while overcoming the dominance of *S. cerevisiae* over NCY. Finally industrial translation will require nontrivial attention to reproduce the critical heat and oxygen transfer patterns through adequate process and equipment design, to accommodate NCY-specific requirements. To this aim, an accurate pilot scale validation involving the accurate control of the surface-to-volume ratio, heat dissipation and the precise control of dissolved oxygen and pH could ensure to define the adequate process conditions to allow successful performances.

**Figure 6:**
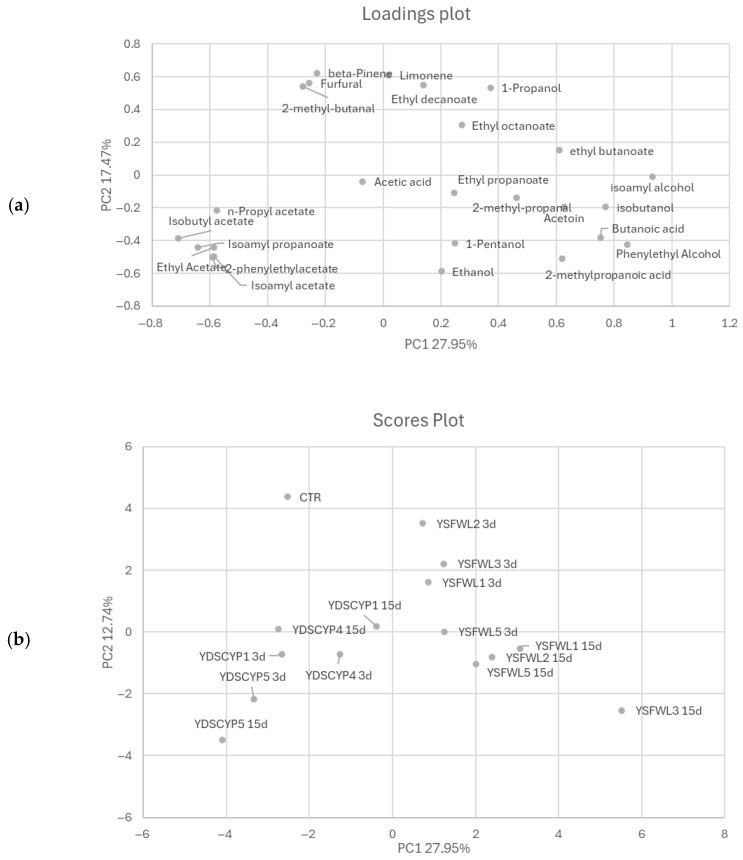
PCA analysis of volatile compounds. Panel (**a**) Loading plot. Panel (**b**) Score plot of VOCs detected with Biomass B.

In order to better understand the differences between the samples a PCA of the volatile compounds was performed Figure 6a,b. The first two PCs explained about 45.42% of the total variance of the data, with PC1 and PC2 explaining 27.95% and 17.47% of the total variance, respectively.

Scores plot of the samples are shown in Figure 6b, and the corresponding loadings, establishing the relative importance of the variables, are shown in Figure 6a. The samples were divided into 2 principal groups based on the relationship between samples (scores) and their volatile compounds (loadings).

As shown by score plot, the strains were distributed according to their origins: one group included all the strains YDSCYPx at 3rd and 15th days while, the other grouped *S. cerevisiae* YSFWLx at the same time intervals. YDSCYPx were characterized by esters and acetates classes compounds such as isoamyl propanoate, isoamyl acetate, 2-phenylethyl acetate, ethylacetate, isobutyl acetate and propylacetate compounds. YSFWLx were characterized by different aromatic compounds such as alcohols, aldehydes, ketones and esters. Finally, uninoculated controls were featured by terpenoids such as limonene and β-pinene and aldehydes, such as furfural e and 2-methylbutanal.

#### 3.8.3. Statistical Interaction and Substrate Influence—Two-Way ANOVA

Following the kinetic characterization, a Two-way ANOVA was specifically conducted on the volatile compounds that had previously shown statistical significance in the ANCOVA models (see Table S3 in Supplementary Materials). This targeted approach allowed for a deeper evaluation of the combined effects of yeast strain (Y) and substrate (S) on the most metabolically active metabolites (ethyl acetate, isoamyl acetate, ethanol, isoamyl alcohol). The analysis revealed a highly significant interaction (Y × S, *p* < 0.001) for these key compounds, particularly for ethyl acetate and isoamyl acetate. These results confirm that while fermentation progression (weight loss) drives the initial synthesis, the final aromatic magnitude is strictly governed by the specific yeast-substrate synergy, with Biomass B acting as a more potent metabolic trigger compared to biomass A.

### 3.9. Effect of Agitation

In order to better confirm and define the fermentative capacities of native yeasts, the abilities to produce aroma compounds and improve their yields particularly of alcohol and ester classes, new batch micro-fermentation experiments under agitation were carried out (28 °C, for 72 h, 180 rpm) utilizing biomass B. This substrate was selected due to the sensibly higher yields of alcohols and esters obtained when it was utilized in fermentation under static conditions respect to biomass A. In particular, with the non-*Saccharomyces* yeasts *W. anomalus* YDSCYP1 and YDSCYP4 and *P. kluyveri* YDSCYP5, 110 and 166% higher yields for alcohols and esters were measured and with native *Saccharomyces* about seven folds higher amounts of alcohol were produced. The non-*Saccharomyces* strains utilized for experiments under agitation were *W. anomalus* YDSCYP4 and *P. kluyveri* YDSCYP5 since they showed a more active fermentative metabolism on biomass B. This was deduced from data of weight loss and ethanol production measured after fermentation in static mode. Among the strains isolated from figs waste, instead, YSFWL3 was chosen, since it proved to be the best isoamyl alcohol producer. According to the literature which reported a limited cellular growth under oxygen limitation for non-*Saccharomyces* strains [38], agitation [61,62] improved *W. anomalus* YDSCYP4 and *P. kluyveri* YDSCYP5 growth. This was confirmed by vital cell counts data that, after 72 h of cultivation, reached values of log 8, exceeding by a logarithmic order those measured under static conditions. Higher oxygen supply, in addition to stimulating respiratory sugar metabolism and consequently cellular growth, enhanced also the long chain alcohol production by all the strains. In particular, as reported in Table 4, the yields of isoamyl alcohol increased by 136 and 40% respect to those measured when *W. anomalus* YDSCYP4 and *P. kluyveri* YDSCYP5 were grown in static mode, while *S. cerevisiae* YSFWL3 produced more 28%. As underlined by several authors, fusel alcohols production is enhanced by oxygen supply [36,37,61,62,63]. These compounds, that originate from 2-oxoacids via decarboxylation and reduction are enhanced when the aerobic metabolism is boosted. 2-oxoacids, in fact, result from the oxidative deamination of correspondent amino acids (Ehrlich pathway) and are intermediates of tricarboxylic acid cycle. Oxygen supplements had a positive effect also on the production of esters that remained the most abundant sensory compounds, as when the growth was conducted under static batch conditions. In particular, under agitation 18 and 14% more ethyl and isoamyl acetate for *P. kluyveri* YDSCYP5 were measured respectively. As reported by several authors, esters formation is regulated by a sensitive balance between positively and negatively affecting factors among which the role of agitation is controversial. Alcohol acyl transferases, key enzymes in acetate esters formation that are highly expressed in *P. kluyveri* [38,49], are inhibited by oxygen [39,40,41,42,56,62,64,65] and negatively affected by aerobic conditions as reported by Saerens et al. [42,66] and Sumby et al. [44,67]. However, Mendez-Zamora et al. [31,44] found that agitation stimulated esters production by two *P. kluyveri* strains grown in different liquid media. The increased stirring speed enhanced the production of acetate esters (ethyl acetate and isoamyl acetate) and the medium chain fatty acid ester ethyl decanoate together with the formation of ethanol and higher alcohols. The agitation in a closed system that was represented by shake flask, was reported as a positive factor also by Rojas et al. [42,56] that evidenced an increased acetate esters production by two non-*Saccharomyces* strains belonging to *P. anomala* and *H. guilliermondii* species. Friedlung et al. [45,68] in this regard clarified that agitation stimulated the production of biomass and promoted ester synthesis that was induced by the shift to anaerobic conditions during growth in closed systems. In fact, as they verified, the anaerobiosis was easily reached in liquid yeast cultures conducted in closed systems of rotating shake flasks, due to shielding O_2_ access to the medium by the CO_2_, produced through the respiratory metabolism. Otherwise, the only anaerobiosis condition inhibited ester formation.

As was evident from our results, the agitation had a positive effect also on the ethanol production by *P. kluyveri* YDSCYP5 that reached more elevated yield than under static cultivation mode. Growth under moderate agitation revealed to be particularly suited for *P. kluyveri* that, as already reported [46,48], produces cellular aggregates as islet formations, on the liquid medium surface. Adequate stirring is very useful to obtain a homogeneous distribution of cells and nutrients that promotes biomass growth, oxidative metabolism and also the onset of the anaerobic conditions. These data are in complete agreement with a similar behavior previously highlighted by Miguel et al. [47,69] that reported an enhanced rate of growth and ethanol production for a strain of *P. kluyveri* grown in liquid synthetic wort medium under agitation. In particular, biomass production was improved as highlighted by the shift in the growth curve from linear to exponential shape when stirring was applied; the linear curve, in fact, indicated the effect of limiting factors such as dissolved oxygen and nutrients availability under static conditions.

Conclusively, the integrative interpretation of fermentation kinetics and VOC generation obtained from fermentation experiments conducted in static mode or under agitation, clearly evidenced that weight loss data strongly correlates with those of ester/alcohol production. Substrate sugars depletion and CO_2_ liberation, through glycolysis, channel to ethanol and fusel pathways involved in volatile aroma compounds production such as higher alcohols and esters. Strains with rapid early weight loss, e.g., YDSCYP4 (3.73 g after 72 h on biomass A), yielded higher isoamyl alcohol/acetate (Table 2 and Table 3), than *S. cerevisiae* YSFWL3, which showed slower kinetic and reduced VOCs among YSFWLx group on the same substrate A despite its fair growth on biomass B. ANCOVA analysis (Tables S1 and S2 in Supplementary Materials) confirms this weight–VOC relationship (*p* < 0.05 per strain).

It was also evident that biomass growth and patterns of aroma molecules production depend on the complex synergy between the metabolic strategies of the different strains and the composition of the substrates. The stressful conditions of biomass A, represented by a lower sugar content, slowed down the *Saccharomyces* YSFWLx growth rate and preferentially boosted their production of ethyl acetate vs. fusel esters suggesting a weaker amino acid catabolism. Moreover, the availability of fermentable sugars in the substrate was found to be the primary determinant of fermentative performance and microbial biomass yield. Biomass B supported faster adaptability, particularly in the *Saccharomyces* YSFWLx group, which produced higher biomass yields (Log CFU/g) and reached stationary phase more quickly (by day 12) than biomass A. Moreover, as demonstrated by higher weight losses, they achieved a more efficient consumption of sugars which, driving a faster NADH/NADPH availability for alcohol dehydrogenase, enhanced the conversion of the saccharide compounds into primary metabolites such as ethanol. This contrasted with the NCY *W. anomalus* and *P. kluyveri*, which preferentially produced acetate esters. These strains better channel overflow to aroma molecules such as fusel compounds utilizing the deamination and decarboxylation reactions of Ehrlich pathway that utilized amino acids as substrates. No stressful condition ensured by the sugar rich biomass B favored non-*Saccharomyces* esterase/arylamidase activity (API-ZYM data see below) leading leucine/valine conversion to isoamyl acetate so explaining ester dominance vs. *S. cerevisiae* ethanol bias. Competition for precursors such as acetyl-CoA for biomass and VOC production was a key metabolic factor in determining the relative abundance of alcohol and ester. *Saccharomyces* strains prioritized the use of acetyl-CoA for high and fast biomass generation, thereby limiting its availability for trans-acetylation reactions and acetate ester production. Esters liberation was maximized only after the exponential growth phase, when sufficient acetyl-CoA was available for VOC synthesis. The metabolic signature of the NCY *W. anomalus* and *P. kluyveri* was characterized by favoring the flux of acetyl-CoA towards acetate production over high biomass and primary metabolite generation. Finally, agitation was found to enhance respiratory metabolism, biomass growth, and higher alcohol production via the Ehrlich pathway, which is based on amino acid oxidative deamination reactions. However, oxidative conditions could inhibit alcohol acetyltransferase activity, which is responsible for ester formation. Nevertheless, the eventual shift to anaerobic conditions in closed shake systems ultimately led to significantly higher ester yields, as demonstrated by *P. kluyveri* YDSCYP5, which produced 18% more ethyl acetate when agitated.

### 3.10. Determination of Enzymatic Profile

The enzyme activities of the three strains *W. anomalus* YDSCYP4, *P. kluyveri* YDSCYP5 and *S. cerevisiae* YSFWL3 were evaluated and the data was reported in Table 5. The results were recorded on result sheets using a value scale ranging from 0 to 5, to assign the colors developed in each cupule of the API strip. The attribution of the value was 0 to indicate negative reaction, 5 maximum reaction intensity; 1, 2, 3 and 4 intermediate reaction ability. In this assay, values 3 and 4 were the only reactions considered to be positive.

The arylamidase and esterase-lipase enzymes that are related to aroma formation, were among the most represented activities. Arylamidases that were detected in all the strains, are responsible for the processive hydrolysis of N terminal moieties of polypeptidic chains releasing amino acids, known precursors of aroma compounds [70]. In particular, leucine arylamidase was highly expressed by *P. kluyveri* YDSCYP5 that also produced the more elevated amounts of isoamyl acetate when biomass B was utilized as substrate under static and agitation conditions. The availability of leucine, in fact, is fundamental for the formation of isoamyl alcohol (isoamyl acetate precursor) that is produced from this branched chain amino acid through the sequential deamination, decarboxylation and reduction reactions of Ehrlich pathway [48,71]. This pathway, which was primarily studied in *S. cerevisiae* by Ehrlich [72] at the beginning of the nineties, requires the availability of a specific amino acid, involved firstly in the transamination reactions catalyzed by aminotransferases enzymes followed by decarboxylation of the resulting α-ketoacids to aldheydes finally reduced by alcohol dehydrogenases to the corresponding alcohols [39,48]. Esterase activities were also detected in the three strains with the highest production levels in *W. anomalus* YDSCYP4 and *P. kluyveri* YDSCYP5. As described by Escribano et al. [49,70], esterase activity is widely represented in *P. kluyveri* species, and all the isolated strains expressed these extracellular activities. Acetate accumulation that results from the balance between the ester synthesizing and hydrolyzing reactions, involves the activity of alcohol acetyl transferase (AAT) and esterases. Acetyl CoA and higher alcohols that are utilized by AAT to be condensed in acetate esters can also be substrates of esterases that through their transesterification activity are able to convert them into acetate esters. Earlier findings compared the utilization of alcohol acetyl transferase (AAT) and inverse esterase (IE) catalyzed reactions in different yeast species for the production of esters such as ethyl and isoamyl acetate starting from the corresponding alcohols [45,68].The authors found that *W. anomalus* (formerly *P. anomala*) preferentially utilizes acetate for ester synthesis in IE catalyzed reactions differently from *S. cerevisiae* that prefers the utilization of acetylCoA in the AAT involving pathway.

### 3.11. Limitations

The present study is a laboratory-scale investigation which employed semi-quantitative VOC analysis to evaluate ten indigenous strains on two agri-food waste substrates. While the study demonstrates technical feasibility and identifies promising strains, future work will focus on bridging the gap between laboratory demonstration and economically sustainable implementation in a biorefinery. A semi-quantitative measurement of VOCs was chosen in order to facilitate a broad preliminary screening of yeast and substrate combinations. Absolute quantification is required for industrial applications; therefore, future studies will prioritize the characterization of the main aromatic compounds identified in this work (e.g., isoamyl acetate and ethyl acetate). For these key metabolites, an absolute quantitative determination will be carried out using external calibration curves and high-purity standards to confirm their exact concentrations and evaluate their Odor Activity Values (OAV). Furthermore, laboratory-scale conditions may differ from industrial processes. However, micro-fermentations were specifically chosen as they provide a highly controlled environment for accurately assessing the growth dynamics of the inoculated strains. This approach ensured accurate monitoring of weight loss, VOC (volatile organic compound) production and microbial kinetics, whilst minimizing external variables and interference. The yeast strains were selected based on rigorous physiological and technological criteria, as the present study aimed to characterize the dynamic interaction between the inoculum and the matrix over time. As regards the substrates, two agro-industrial by-products were chosen to explore the valorization of plant waste through yeast fermentation. These matrices were utilized as unconventional model culture media to assess the metabolic adaptability and aromatic potential of the selected indigenous yeast strains.

The reported results should be viewed primarily as an important proof of concept justifying continued investment in NCY research to produce flavorings from agri-food waste, rather than as an industrial solution ready for implementation. In the context of sustainable biotechnology, these results demonstrate that fermentation technologies can successfully transform environmentally harmful waste into high-value-added molecules, providing a ‘natural’ alternative to petroleum-based chemical synthesis.

## 4. Conclusions

This work aims to advance the frontiers of “white biotechnology” and the circular economy by establishing a closed-loop biorefinery model that utilizes agro-food waste (AFW) as both a source of microbial biodiversity and a functional substrate. Native non-conventional yeasts (NCY), *W. anomalus* and *P. kluyveri*, were identified as superior biocatalysts for the synthesis of high-value acetate esters. In contrast, *S. cerevisiae* strains proved more efficient for ethanol and higher alcohol production. Moreover, it provided a mechanistic link between extracellular enzymatic profiles and the synthesis of specific “natural” aroma molecules. These findings demonstrate a viable pathway for resource upcycling, transforming zero-cost, environmentally burdensome organic waste into source of high-value molecules. However, harnessing the results of this research for applications in the biorefinery sector requires integrated approaches that combine strain improvement, process optimization, substrate standardization and economic analysis, in order to realize the potential of NCY-based biorefinery processes.

## Figures and Tables

**Table 1:** Overview of the morphological, physiological features, and molecular identification at specie level of isolated native yeasts strains.

Strains Code	AFW Substrate	Cell Morphology	Spore	O.D.	Carbohydrates ^a^*				Identification
					Gl	Fr	Su	Ma	
**YDSCYP1**	biomass “**a**”	ovoid to elongate	**+**	1.26	**+++**	**+**	**++**	**a**	***Wickerhamomyces anomalus***
**YDSCYP2**	biomass “**a**”	cylindrical, pseudohyphae	**-**	0.50	**+++**	**++**	**++**	**-**	***Pichia californica***
**YDSCYP3**	biomass “**a**”	ovoid to elongate	**+**	0.60	**+++**	**++**	**++**	**-**	***Pichia kluyveri***
**YDSCYP4**	biomass “**a**”	ovoid to elongate	**-**	0.70	**+++**	**++**	**++**	**++**	***Wickerhamomyces anomalus***
**YDSCYP5**	biomass “**a**”	ovoid to elongate	**-**	0.80	**+++**	**++**	**++**	**-**	***Pichia kluyveri***
**YSFWL1**	biomass “**b**”	Ovoidal	**+**	2.60	**+++**	**++**	**++**	**a**	***Saccharomyces cerevisiae***
**YSFWL2**	biomass “**b**”	Cylindric	**-**	1.54	**+++**	**+**	**++**	**a**	***Saccharomyces cerevisiae***
**YSFWL3**	biomass “**b**”	Round	**+**	2.40	**+++**	**++**	**++**	**a**	***Saccharomyces cerevisiae***
**YSFWL4**	biomass “**b**”	ovoidal	**+**	0.55	**+++**	**++**	**++**	**-**	***Saccharomyces cerevisiae***
**YSFWL5**	biomass “**b**”	Round	**+**	2.10	**+++**	**++**	**++**	**-**	***Saccharomyces cerevisiae***

* Carbohydrates abbreviation: glucose (Gl), fructose (Fr), sucrose (Su) and maltose (Ma). ^a^ Growth on different carbohydrates: “+++” strong (1 week) “++” medium (2 week); “+” weak (3 week); “+, -” positive or negative fermentative capabilities; a = assimilation capability.

**Table 2:** Volatile organic compounds (VOCs) identified by solid phase microextraction/gas chromatography-mass spectrometry from sample collected after 3 and 15 days from batch fermentation conducted in static mode utilizing biomass A as substrate and YDSCYPx and YSFWLx strains as inocula.

		CTR	YP1 3d	YP4 3d	YP5 3d	YP1 15d	YP4 15d	YP5 15d	WL1 3d	WL2 3d	WL3 3d	WL5 3d	WL1 15d	WL2 15d	WL3 15d	WL5 15d
RI	Aldehydes															
810	2-methylpropanal	nd ^d^	nd ^d^	nd ^d^	nd ^d^	nd ^d^	nd ^d^	nd ^d^	nd ^d^	nd ^d^	nd ^d^	nd ^d^	56.6 ± 1.3 ^c^	nd ^d^	92.5 ± 0.3 ^a^	80.8 ± 1.7 ^b^
932	2-methylbutanal	nd ^b^	nd ^b^	nd ^b^	nd ^b^	nd ^b^	nd ^b^	nd ^b^	nd ^b^	nd ^b^	nd ^b^	nd ^b^	nd ^b^	nd ^b^	nd ^b^	95.5 ± 2.0 ^a^
1470	furfural	7.7 ± 0.1 ^a^	nd ^b^	nd ^b^	nd ^b^	nd ^b^	nd ^b^	nd ^b^	nd ^b^	nd ^b^	nd ^b^	nd ^b^	nd ^b^	nd ^b^	nd ^b^	nd ^b^
	tot	7.7 ± 0.1	nd	nd	nd	nd	nd	nd	nd	nd	nd	nd	56.6 ± 0.6	nd	92.5 ± 0.3	176.3 ± 1.9
	Ketones															
1290	acetoin	nd ^f^	nd ^f^	nd ^f^	nd ^f^	2.4 ± 0.1 ^e^	3.3 ± 0.1 ^d^	nd ^f^	nd ^f^	nd ^f^	nd ^f^	nd ^f^	15.5 ± 0.2 ^a^	14.7 ± 0.2 ^b^	2.5 ± 0.1 ^e^	6.6 ± 0.2 ^c^
	tot	nd	nd	nd	nd	2.4 ± 0.1	3.3 ± 0.1	nd	nd	nd	nd	nd	15.5 ± 0.2	14.7 ± 0.2	2.5 ± 0.1	6.6 ± 0.2
	Esters and Acetates															
906	ethyl acetate	522.4 ± 22.5 ^f^	2674.9 ± 337.1 ^b^	2851.6 ± 191.2 ^a^	865.8 ± 14.1 ^d^	23.9 ± 0.7 ^h^	nd ^i^	1465.5 ± 23.0 ^c^	90.7 ± 0.9 ^gh^	165.9 ± 0.4 ^g^	35.4 ± 0.2 ^h^	41.4 ± 0.7 ^h^	760.0 ± 12.2 ^de^	730.1 ± 13.5 ^e^	500.4 ± 14.1 ^f^	439.0 ± 16.4 ^f^
957	propyl acetate	9.4 ± 0.2 ^d^	77.7 ± 0.1 ^b^	142.4 ± 2.4 ^a^	nd ^e^	nd ^e^	nd ^e^	50.3 ± 9.0 ^c^	nd ^e^	nd ^e^	nd ^e^	nd ^e^	nd ^e^	nd ^e^	nd ^e^	nd ^e^
1075	butyl acetate	nd ^d^	16.6 ± 0.2 ^a^	13.0 ± 0.2 ^b^	19.2 ± 0.7 ^c^	nd	nd	16.9 ± 0.8 ^a^	nd ^d^	nd ^d^	nd ^d^	nd ^d^	nd ^d^	nd ^d^	nd ^d^	nd ^d^
1130	isoamyl acetate	4.33 ± 0.25 ^i^	227.5 ± 4.4 ^e^	328.9 ± 2.1 ^c^	1014.1 ± 19.3 ^a^	nd ^i^	nd ^i^	417.5 ± 15.8 ^b^	90.7 ± 0.5 ^g^	149.1 ± 1.7 ^f^	24.1 ± 0.1 ^h^	32.2 ± 0.4 ^h^	267.5 ± 15.4 ^d^	7.4 ± 0.2 ^ii^	26.2 ± 1.2 ^h^	7.0 ± 0.1 ^i^
1245	ethyl hexanoate	nd ^e^	nd ^e^	nd ^e^	nd ^e^	nd ^e^	nd ^e^	nd ^e^	nd ^e^	32.1 ± 0.1 ^c^	84.2 ± 0.2 ^a^	54.8 ± 1.0 ^b^	17.6 ± 0.7 ^d^	nd ^e^	nd ^e^	nd ^e^
1267	hexyl acetate	nd ^e^	nd ^e^	1.12 ± 0.02 ^d^	14.55 ± 0.23 ^a^	nd ^e^	nd ^e^	3.38 ± 0.15 ^b^	nd ^e^	15.4 ± 0.4 ^a^	2.1 ± 0.1 ^c^	nd ^e^	nd ^e^	nd ^e^	nd ^e^	nd ^e^
1412	ethyl octanoate	nd ^f^	nd ^f^	nd ^f^	11.50 ± 0.17 ^c^	5.6 ± 0.1 ^e^	nd ^f^	nd ^f^	nd ^f^	8.9 ± 0.1 ^d^	448.5 ± 2.2 ^a^	105.3 ± 0.4 ^b^	nd ^f^	nd ^f^	nd ^f^	nd ^f^
1630	ethyl decanoate	nd^d^	nd ^d^	nd ^d^	nd ^d^	nd ^d^	nd ^d^	nd ^d^	5.37 ± 0.03 ^c^	nd ^d^	201.7 ± 8.3 ^a^	26.3 ± 1.0 ^b^	nd ^d^	nd ^d^	nd ^d^	nd ^dc^
1820	2-phenethylacetate	nd ^g^	15.1 ± 0.1 ^c^	26.7 ± 1.5 ^b^	132.4 ± 0.6 ^a^	2.5 ± 0.1 ^f^	nd ^g^	10.4 ± 2.2 ^d^	nd ^g^	nd ^g^	6.1 ± 0.1 ^e^	nd ^g^	nd ^g^	nd ^g^	nd ^g^	nd ^g^
	tot	536.1 ± 2.9	3011.8 ± 4.8	3363.8 ± 3.2	2057.6 ± 4.4	32.0 ± 0.1	nd	1964.0 ± 6.5	186.1 ± 0.0	371.1 ± 0.4	802.1 ± 1.6	260.1 ± 0.5	1045.1 ± 4.0	737.5 ± 1.9	526.6 ± 2.2	446.0 ± 3.3
	Alcohols															
942	ethanol	82.5 ± 0.1 ^i^	975.7 ± 1.5 ^g^	935.0 ± 2.2 ^g^	912.1 ± 16.1 ^gh^	1.6 ± 0.1 ^i^	nd ^i^	798.2 ± 27.8 ^h^	3842.4 ± 59.8 ^b^	3064.7 ± 40.7 ^c^	1338.0 ± 27.5 ^f^	1407.0 ± 2.2 ^f^	4279.3 ± 28.0 ^a^	2017.0 ± 17.3 ^e^	2620.0 ± 42.1 ^d^	3627.0 ± 110.8 ^c^
1048	1-propanol	nd ^f^	nd ^f^	nd ^f^	nd ^f^	nd ^f^	nd ^f^	nd ^f^	4.9 ± 0.1 ^d^	nd ^f^	nd ^f^	nd ^f^	12.7 ± 0.2 ^c^	14.6 ± 0.3 ^b^	27.8 ± 1.4 ^a^	3.6 ± 0.1 ^e^
1124	isobutanol	nd ^h^	nd ^h^	nd ^h^	5.7 ± 0.2 ^g^	nd ^h^	nd ^h^	5.08 ± 0.9 ^g^	31.4 ± 0.1 ^c^	11.5 ± 0.4 ^f^	nd ^h^	nd ^h^	55.9 ± 1.3 ^a^	21.8 ± 1.6 ^d^	40.5 ± 1.0 ^b^	14.8 ± 0.3 ^e^
1150	1-butanol	nd ^e^	nd ^e^	nd ^e^	nd ^e^	nd ^e^	nd ^e^	nd ^e^	33.55 ± 0.24 ^a^	nd ^e^	nd ^e^	nd ^e^	24.1 ± 1.2 ^c^	nd ^e^	19.0 ± 1.6 ^d^	28.1 ± 0.7 ^b^
1210	isoamyl alcohol	nd ^l^	29.4 ± 0.8 ^f^	25.4 ± 1.6 ^g^	nd ^i^	12.2 ± 0.1 ^h^	19.3 ± 1.7 ^i^	13.7 ± 1.0 ^h^	1139.3 ± 11.2 ^b^	908.8 ± 19.1 ^c^	110.2 ± 0.6 ^e^	169.3 ± 1.2 ^d^	1572.2 ± 1.4 ^a^	1194.0 ± 22.1 ^b^	1202.5 ± 21.6 ^b^	1231.9 ± 34.1 ^b^
1844	benzyl alcohol	10.6 ± 0.2 ^c^	6.5 ± 0.1 ^e^	4.6 ± 0.1 ^f^	4.4 ± 0.1 ^f^	1.2 ± 0.1 ^gh^	0.7 ± 0. 1 ^hi^	8.2 ± 0.5 ^d^	3.1 ± 0.1 ^fg^	nd ^i^	15.3 ± 0.2 ^a^	13.7 ± 0.1 ^b^	3.2 ± 0.1 ^fg^	nd ^i^	nd ^i^	nd ^i^
1902	phenylethanol	nd ^f^	13.3 ± 0.1 ^c^	11.1 ± 0.2 ^cd^	4.0 ± 0.1 ^ef^	26.9 ± 0.8 ^a^	25.9 ± 1.5 ^a^	26.5 ± 1.7 ^a^	2.5 ± 0.1 ^f^	14.2 ± 0.3 ^c^	21.0 ± 1.6 ^b^	6.9 ± 0.1 ^de^	13.2 ± 0.1 ^c^	6.9 ± 0.1 ^de^	8.8 ± 0.1 ^d^	nd ^f^
	tot	93.1 ± 0.1	1024.7 ± 0.5	976.1 ± 4.6	926.1 ± 3.3	42.6 ± 0.2	46.0 ± 0.6	852.2 ± 7.9	5057.2 ± 10.2	3999.2 ± 12.1	1484.5 ± 7.5	1596.9 ± 0.9	5960.7 ± 4.6	3254.4 ± 5.9	3918.5 ± 9.7	4905.4 ± 29.2
	Acids															
1445	acetic acid	4.4 ± 0.2 ^h^	61.8 ± 0.5 ^c^	61.6 ± 1.4 ^c^	13.4 ± 0.1 ^g^	nd ^l^	1.4 ± 0.1 ^i^	87.1 ± 15.6 ^b^	55.6 ± 0.1 ^d^	218.3 ± 15.5 ^a^	12.79 ± 0.35 ^g^	7.9 ± 0.1 ^h^	40.6 ± 2.1 ^e^	32.9 ± 0.6 ^f^	38.8 ± 0.2 ^f^	35.9 ± 0.6 ^f^
1580	2-methypropanoic acid	nd ^g^	1.0 ± 0.1 ^f^	nd ^g^	nd ^g^	3.2 ± 0.1 ^e^	3.0 ± 0.1 ^e^	2.6 ± 0.2 ^e^	4.7 ± 0.1 ^d^	11. 7 ± 0.3 ^b^	nd ^g^	nd ^g^	23.1 ± 0.6 ^a^	11.4 ± 0.2 ^b^	8.1 ± 0.1 ^c^	7.5 ± 0.2 ^c^
1624	butanoic acid	nd ^e^	nd ^e^	nd ^e^	nd ^e^	nd ^e^	nd ^e^	nd ^e^	nd ^e^	9.7 ± 0.1 ^a^	0.6 ± 0.00 ^d^	nd ^e^	4.5 ± 0.1 ^b^	2.9 ± 0.2 ^c^	nd ^e^	nd ^e^
	2-methylhexanoic acid	nd ^g^	0.8 ± 0.1	nd ^g^	nd ^g^	nd ^g^	32.4 ± 1.2 ^a^	5.1 ± 0.1 ^d^	1.3 ± 0.1 ^fg^	10.1 ± 0.1 ^b^	nd ^g^	nd ^g^	8.3 ± 0.1 ^c^	4.2 ± 0.1 ^de^	3.1 ± 0.1 ^ef^	nd ^g^
2030	octanoic acid	nd ^b^	nd ^b^	nd ^b^	nd ^b^	nd ^b^	nd ^b^	nd ^b^	nd ^b^	nd ^b^	6.27 ± 0.01 ^a^	nd ^b^	nd ^b^	nd ^b^	nd ^b^	nd ^b^
	tot	4.2 ± 0.2	63.77 ± 0.7	61.6 ± 1.4	13.4 ± 0.1	3.2 ± 0.1	36.8 ± 1.2	94.87 ± 10.1	61.6 ± 0.1	249.7 ± 16.0	19.6 ± 0.6	7.9 ± 0.1	76.4 ± 1.5	51.4 ± 1.0	49.9 ± 0.4	43.4 ± 0.6
	Terpenoids															
1154	β-myrcene	3.3 ± 0.2 ^c^	2.3 ± 0.1 ^d^	8.1 ± 0.1 ^a^	0.6 ± 0.1 ^g^	1.4 ± 0.1 ^f^	nd ^h^	1.7 ± 0.1 ^e^	nd ^h^	nd ^h^	3.2 ± 0.1 ^c^	5.0 ± 0.1 ^b^	nd ^h^	nd ^h^	nd ^h^	nd ^h^
1547	limonene	13.6 ± 0.4 ^e^	8.6 ± 0.10 ^ef^	23.6 ± 1.8 ^d^	5.2 ± 0.1 ^fgh^	6.9 ± 0.1 ^fg^	7.8 ± 0.1 ^ef^	5.6 ± 0.1 ^egh^	nd ^h^	35.5 ± 0.9 ^c^	74.9 ± 0.1 ^b^	304.7 ± 8.6 ^a^	nd ^h^	nd ^h^	nd ^h^	nd ^h^
	tot	16.9 ± 1.1	10.9 ± 0.1	31.7 ± 1.9	5.8 ± 0.1	8.3 ± 0.1	7.8 ± 0.1	7.3 ± 0.1	nd	35.5 ± 0.9	78.1 ± 0.1	309.7 ± 2.5	nd	nd	nd	nd

Abbreviations: nd, not detected; RI = retention index, identification via comparison with RI database for a high polar column for InnoWAX or similar stationary phases [24]; YPx for YDSCYPx; WLx for YSFWLx. Results are expressed as RAP = relative peak area (peak area of compound/peak area of internal standard) ⇥ 100 (RAP ± SD). Compounds with different letters in the same row are significantly different according to the Duncan’s test (*p* < 0.05).

**Table 3:** Volatile organic compounds (VOCs) identified by solid phase microextraction/gas chromatography-mass spectrometry from sample collected after 3 and 15 days from batch fermentation conducted in static mode utilizing biomass B as substrate and YDSCYPx and YSFWLx strains as inocula.

		CTR	YP1 3d	YP4 3d	YP5 3d	YP1 15d	YP4 15d	YP5 15d	WL1 3d	WL2 3d	WL3 3d	WL5 3d	WL1 15d	WL2 15d	WL3 15d	WL5 15d
RI	Aldehydes															
810	2-methylpropanal	7.4 ± 0.7 ^b^	nd ^c^	nd ^c^	nd ^c^	nd ^c^	nd ^c^	nd ^c^	nd ^c^	nd ^c^	nd ^c^	nd ^c^	nd ^c^	nd ^c^	22.7 ± 1.0 ^b^	nd ^c^
932	2-methylbutanal	4.1 ± 0.5 ^a^	nd ^c^	0.8 ± 0.1 ^b^	nd ^c^	nd ^c^	nd ^c^	nd ^c^	nd ^c^	nd ^c^	nd ^c^	nd ^c^	nd ^c^	nd ^c^	nd ^c^	nd ^c^
1470	furfural	3.6 ± 0.5 ^a^	nd ^b^	nd ^b^	nd ^b^	nd ^b^	nd ^b^	nd ^b^	nd ^b^	nd ^b^	nd ^b^	nd ^b^	nd ^b^	nd ^b^	nd ^b^	nd ^b^
	tot	15.1 ± 1.1	nd	0.8 ± 0.1	nd	nd	nd	nd	nd	nd	nd	nd	nd	nd	22.7 ± 1.0	nd
	Ketones															
1290	acetoin	9.7 ± 1.0 ^e^	nd ^h^	nd ^h^	nd ^h^	13.1 ± 0.1 ^e^	nd ^h^	nd ^h^	4.6 ± 0.2 ^fg^	2.2 ± 0.2 ^gh^	5.8 ± 0.2 ^f^	83.0 ± 0.3 ^c^	118.2 ± 0.8 ^a^	1.6 ± 0.1 ^gh^	60.8 ± 3.6 ^d^	88.1 ± 2.1 ^b^
	tot	9.7 ± 1.0	nd	nd	nd	13.1 ± 0.1	nd	nd	4.6 ± 0.2	2.2 ± 0.2	5.8 ± 0.2	83.0 ± 0.3	118.2 ± 0.8	1.6 ± 0.1	60.8 ± 3.6	88.1 ± 2.1
	Esters and Acetates															
906	ethyl acetate	23.7 ± 3.2 ^hi^	5665.2 ± 134.5 ^a^	5117.1 ± 78.7 ^b^	3455.0 ± 29.0 ^d^	315.0 ± 11.0 ^e^	41.4 ± 1.9 ^ghi^	3663.2 ± 130.4 ^c^	188.98 ± 18.47 ^efgh^	273.7 ± 23.3 ^ef^	nd ^i^	178.4 ± 9.7 ^efghi^	223.6 ± 12.3 ^efg^	157.9 ± 5.4 ^efghi^	94.6 ± 2.9 ^fghi^	210.6 ± 14.3 ^efg^
952	ethyl propanoate	nd ^c^	nd ^c^	nd ^c^	nd ^c^	nd ^c^	nd ^c^	nd ^c^	nd ^c^	nd ^c^	nd ^c^	5.8 ± 0.2 ^b^	nd ^c^	nd ^c^	nd ^c^	7.4 ± 0.1 ^a^
957	propyl acetate	19.1 ± 2.2 ^c^	237.5 ± 13.0 ^a^	nd ^d^	nd ^d^	nd ^d^	236.9 ± 14.4 ^a^	144.7 ± 1.3 ^b^	nd ^d^	nd ^d^	nd ^d^	nd ^d^	nd ^d^	nd ^d^	nd ^d^	nd ^d^
990	isobutyl acetate	21.2 ± 2.0 ^d^	24.3 ± 1.2 ^c^	nd ^h^	68.7 ± 1.2 ^b^	6.9 ± 0.1 ^g^	20.1 ± 1.0 ^d^	102.8 ± 0.2 ^a^	13.8 ± 0.4 ^f^	17.8 ± 0.7 ^e^	nd ^h^	nd ^h^	nd ^h^	nd ^h^	nd ^h^	nd ^h^
1042	ethyl butanoate	nd ^f^	nd ^f^	nd ^f^	nd ^f^	nd ^f^	nd ^f^	nd ^f^	0.9 ± 0.03 ^c^	1.3 ± 0.1 ^b^	nd ^f^	nd ^f^	1.5 ± 0.02 ^a^	0.7 ± 0.02 ^e^	0.8 ± 0.02 ^d^	nd ^f^
1130	isoamyl acetate	53.9 ± 8.1 ^ef^	453.3 ± 8.1 ^cd^	491. 7 ± 20.5 ^c^	3840.8 ± 176.4 ^a^	34.2 ± 0.8 ^ef^	0.9 ± 0.1 ^f^	3537.4 ± 16.13 ^b^	306.3 ± 16.0 ^d^	397.6 ± 23.1 ^cd^	162.2 ± 5.2 ^e^	nd ^f^	16.6 ± 1.2 ^ef^	26.1 ± 0.9 ^ef^	36.1 ± 0.8 ^ef^	10.3 ± 0.05 ^ef^
1184	isoamyl propanoate	nd ^d^	nd ^d^	nd ^d^	8.6 ± 0.1 ^a^	nd ^d^	8.1 ± 0.01 ^b^	7.90 ± 0.02 ^c^	nd ^d^	nd ^d^	nd ^d^	nd ^d^	nd ^d^	nd ^d^	nd ^d^	nd ^d^
1412	ethyl octanoate	nd ^de^	2.1 ± 0.1 ^d^	nd ^e^	nd ^e^	nd ^e^	nd ^e^	nd ^e^	7.68 ± 0.41 ^b^	nd ^e^	16.1 ± 0.1 ^a^	6.6 ± 0.2 ^c^	6.4 ± 0.1 ^c^	nd ^e^	nd ^e^	nd ^e^
1630	Ethyl decanoate	nd ^c^	nd ^c^	nd ^c^	nd ^c^	nd ^c^	nd ^c^	nd ^c^	nd ^c^	7.89 ± 0.22 ^a^	6.1 ± 0.2 ^b^	nd ^c^	nd ^c^	nd ^c^	nd ^c^	nd ^c^
1820	2-phenylethylacetate	30.7 ± 1.0 ^cd^	9.2 ± 0.1 ^fg^	38.2 ± 2.1 ^c^	170.2 ± 11.0 ^b^	17.9 ± 0.4 ^ef^	23.4 ± 1.6 ^de^	396.4 ± 7.1 ^a^	8.0 ± 0.4 ^fg^	4.73 ± 0.20 ^g^	4.1 ± 0.1 ^g^	nd ^h^	nd ^h^	nd ^h^	nd ^h^	nd ^h^
	tot	148.5 ± 13.1	6391.7 ± 158.0	5156.2 ± 73.9	7543.3 ± 137.7	374.1 ± 12.2	821.5 ± 21.2	7852.8 ± 105.6	525.6 ± 35.7	715.89 ± 47.8	188.5 ± 5.2	190.8 ± 9.4	252.0 ± 13.6	186.0 ± 6.4	131.5 ± 2.2	233.6 ± 14.3
	Alcohols															
942	ethanol	254.2 ± 13.7 ^gh^	2368.3 ± 198.6 ^b^	2230.0 ± 84.6 ^bc^	1606.3 ± 2.6 ^e^	nd ^h^	310.2 ± 14.3 ^gh^	1617.0 ± 6.2 ^e^	509.2 ± 36.5 ^g^	511.4 ± 9.7 ^g^	845.3 ± 37.5 ^f^	1710.5 ± 6.4 ^de^	2296.9 ± 235.4 ^bc^	1999.0 ± 196.4 ^cd^	1440.6 ± 36.9 ^e^	2981.0 ± 72.5 ^a^
1048	1-propanol	nd ^g^	nd ^g^	nd ^g^	nd ^g^	nd ^g^	nd ^g^	nd ^g^	12.8 ± 0.5 ^c^	19.1 ± 1.1 ^a^	13.9 ± 0.2 ^b^	nd ^g^	3.2 ± 0.1 ^f^	5.5 ± 0.02 ^d^	4.5 ± 0.1 ^e^	nd ^g^
1124	isobutanol	6.2 ± 0.6 ^i^	5.7 ± 0.1 ^i^	22.0 ± 1.5 ^h^	52.8 ± 2.1 ^e^	47.3 ± 0.8 ^fg^	3. 8 ± 0.2 ^i^	44.0 ± 0.6 ^g^	82.6 ± 3.3 ^c^	54.6 ± 1.8 ^e^	88.5 ± 0.8 ^b^	50.1 ± 0.2 ^ef^	69.8 ± 1.5 ^d^	52.2 ± 0.1 ^e^	134.5 ± 1.3 ^a^	54.7 ± 0.2 ^e^
1210	isoamyl alcohol	150.7 ± 12.8 ^h^	310.2 ± 20.1 ^g^	409.7 ± 24.9 ^f^	143.4 ± 2.7 ^h^	90.1 ± 1.5 ^m^	126.9 ± 1.9 ^i^	101.2 ± 0.4 ^L^	1947.3 ± 61.7 ^i d^	1627.4 ± 54.5 ^e^	1896.9 ± 150.0 ^de^	1960.1 ± 108.7 ^d^	2052.6 ± 86.5 ^cd^	2408.9 ± 52.8 ^b^	2942.2 ± 267.6 ^a^	2299.0 ± 131.2 ^bc^
1263	1-pentanol	nd ^f^	nd ^f^	7.9 ± 0.2 ^c^	nd ^f^	8.6 ± 0.2 ^b^	nd ^f^	7.1 ± 0.1 ^d^	nd ^f^	nd ^f^	3.4 ± 0.2 ^e^	nd ^f^	nd ^f^	nd ^f^	154.0 ± 0.2 ^a^	nd ^f^
1902	phenylethanol	2.4 ± 0.1 ^h^	8.0 ± 0.1 ^g^	31.4 ± 1.4 ^d^	5.75 ± 0.1 ^gh^	32.2 ± 0.1 ^d^	6.8 ± 0.1 ^g^	25.7 ± 1.1 ^e^	20.8 ± 1.6 ^f^	17.1 ± 1.6 ^f^	28.8 ± 0.2 ^de^	24.8 ± 0.4 ^e^	63.1 ± 0.2 ^b^	52.2 ± 3.4 ^c^	90.2 ± 1.1 ^s^	62.1 ± 1.8 ^b^
	tot	413.5 ± 14.2	2692.2 ± 198.5	2701.0 ± 28.0	1808.3 ± 7.3	178.3 ± 0.7	447.7 ± 86.9	1795.0 ± 5.3	2572.7 ± 104.1	2229.6 ± 68.7	2876.9 ± 186.1	3745.5 ± 102.1	4485.6 ± 146.8	4517.9 ± 147.2	4626.9 ± 302.3	5396.8 ± 60.8
	Acids															
1445	acetic acid	29.8 ± 1.3 ^gh^	313.2 ± 0.1 ^a^	249.8 ± 8.8 ^b^	81.0 ± 0.5 ^e^	34.0 ± 1.0 ^gh^	27.2 ± 1.5 ^gh^	23.0 ± 1.2 ^h^	51.6 ± 2.5 ^f^	172.4 ± 14.6 ^c^	90.3 ± 4.3 ^e^	nd ^i^	40.5 ± 1.8 ^fg^	86.2 ± 0.8 ^e^	135.6 ± 3.1 ^d^	36.3 ± 1.4 ^gh^
1580	2-methylpropanoic acid	4.3 ± 0.3 ^e^	2.5 ± 0.1 ^f^	5.3 ± 0.1 ^e^	20.8 ± 0.4 ^c^	14.8 ± 0.1 ^d^	3.9 ± 0.1 ^g^	22.5 ± 1.8 ^c^	3.6 ± 0.2 ^e^	4.5 ± 0.2 ^e^	5.8 ± 0.1 ^e^	14.4 ± 0.4 ^d^	38.7 ± 1.8 ^b^	67.08 ± 0.9 ^a^	69.6 ± 3.4 ^a^	15.3 ± 0.1 ^d^
1624	butanoic acid	nd ^f^	nd ^f^	nd ^f^	nd ^f^	nd ^f^	nd ^f^	nd ^f^	nd ^f^	nd ^f^	nd ^f^	2.5 ± 0.1 ^e^	4.3 ± 0.2 ^c^	10.4 ± 0.4 ^b^	16.1 ± 0.2 ^a^	3.3 ± 0.1 ^d^
	tot	34.1 ± 1.6	315.7 ± 0.1	255.1 ± 1.4	101.8 ± 0.9	48.8 ± 2.1	31.1 ± 8.9	45.5 ± 3.0	55.2 ± 2.1	176.9 ± 13.1	96.1 ± 4.2	16.9 ± 0.5	83.5 ± 1.9	163.7 ± 0.8	221.4 ± 6.7	55.0 ± 1.5
	Terpenoids															
1112	β pinene	6.6 ± 0.4 ^a^	nd ^d^	nd ^d^	nd ^d^	nd ^d^	nd ^d^	nd ^d^	0.5 ± 0.05 ^c^	nd ^d^	1.1 ± 0.01 ^b^	nd ^d^	nd ^d^	nd ^d^	nd ^d^	nd ^d^
1181	limonene	3.4 ± 0.1 ^b^	nd ^d^	nd ^d^	nd ^d^	nd ^d^	nd ^d^	nd ^d^	1.5 ± 0.05 ^c^	13.4 ± 0.3 ^a^	nd ^d^	nd ^d^	nd ^d^	nd ^d^	nd ^d^	nd ^d^
	tot	10.0 ± 0.5	nd	nd	nd	nd	nd	nd	2.0 ± 0.1	13.4 ± 0.3	1.1 ± 0.01	nd	nd	nd	nd	nd

Abbreviations: nd, not detected; RI = retention index, identification via comparison with RI database for a high polar column for InnoWAX or similar stationary phases, [24]; YPx for YDSCYPx; WLx for YSFWLx. Results are expressed as RAP = relative peak area (peak area of compound/peak area of internal standard) ⇥ 100 (RAP ± SD). Compounds with different letters in the same row are significantly different according to the Duncan’s test (*p* < 0.05).

**Table 4:** Volatile organic compounds (VOCs) identified by solid phase microextraction/gas chromatography-mass spectrometry from sample collected after 72 h from batch fermentation conducted in under agitation utilizing biomass B as substrate and *W. anomalus* YDSCYP4, *P. kluyveri* YDSCYP5 and *S. cerevisiae* YSFWL3 strains as inocula.

RI		CTR	YSFWL3 3d	YDSCYP4 3d	YDSCYP5 3d
	Aldehydes				
810	2-methylpropanal	7.47 ± 0.99 ^b^	nd ^c^	nd ^c^	66.01 ± 0.10 ^a^
932	2-methylbutanal	4.45 ± 0.20 ^a^	nd ^b^	nd ^b^	nd ^b^
1470	furfural	2.81 ± 0.02 ^a^	nd ^b^	nd ^b^	nd ^b^
	tot	14.73 ± 2.74	nd	nd	66.01 ± 0.10
	Ketones				
1290	acetoin	7.25 ± 0.60 ^c^	17.65 ± 1.06 ^b^	55.82 ± 0.47 ^a^	2.85 ± 0.14 ^d^
	tot	7.25 ± 0.60	17.65 ± 1.06	55.82 ± 0.47	2.85 ± 0.14
	Esters and Acetates				
906	ethyl Acetate	33.01 ± 1.13 ^c^	270.66 ± 2.69 ^b^	4227.20 ± 3.42 ^a^	4088.0 ± 104.5 ^a^
952	ethyl propanoate	nd ^b^	nd ^b^	nd ^b^	800.84 ± 11.54 ^a^
957	propyl acetate	10.28 ± 0.20 ^b^	nd ^c^	nd ^c^	558.43 ± 5.15 ^a^
990	isobutyl acetate	16.32 ± 1.10 ^d^	40.02 ± 2.33 ^c^	121.79 ± 0.49 ^b^	161.10 ± 1.24 ^a^
1042	ethyl butanoate	nd ^c^	nd ^c^	1.09 ± 0.0 ^a^	4.89 ± 0.11 ^a^
1130	isoamyl acetate	52.01 ± 0.15 ^c^	372.07 ± 17.19 ^b^	366.91 ± 3.07 ^b^	4396.80 ± 128.84 ^a^
1184	isoamyl propanoate	nd ^b^	nd ^b^	nd ^b^	88.89 ± 2.64 ^a^
1267	hexyl acetate	4.25 ± 0.21 ^b^	12.61 ± 0.23 ^a^	nd ^d^	2.92 ± 0.13 ^c^
1412	ethyl octanoate	nd ^d^	32.27 ± 1.06 ^b^	2.61 ± 0.17 ^c^	67.72 ± 0.22 ^a^
1530	ethyl nonanoate	2.11 ± 0.0 ^bc^	3.07 ± 0.23 ^a^	1.96 ± 0.18 ^c^	2.75 ± 0.23 ^ab^
1630	ethyl decanoate	2.96 ± 0.12 ^b^	nd ^c^	nd ^c^	3.70 ± 0.04 ^a^
1820	2-phenylethylacetate	28.37 ± 0.55 ^b^	3.50 ± 0.18 ^d^	5.06 ± 0.22 ^c^	178.46 ± 7.32 ^a^
	tot	149.31 ± 1.50	737.26 ± 18.76	4726.62 ± 1.11	10,354.51 ± 4.4
	Alcohols				
942	ethanol	295.72 ± 1.14 ^c^	296.37 ± 12.49 ^c^	1264.81 ± 29.64 ^b^	18,078.82 ± 894.9 ^a^
1048	1-propanol	nd ^c^	22.05 ± 1.73 ^a^	8.16 ± 0.20 ^b^	nd ^c^
1124	isobutanol	nd ^d^	171.91 ± 16.95 ^a^	45.92 ± 2.65 ^c^	119.77 ± 2.73 ^b^
1210	isoamyl alcohol	140.23 ± 0.24 ^c^	2435.66 ± 76.15 ^a^	968.25 ± 34.61 ^b^	208.88 ± 8.88 ^c^
1263	1-pentanol	6.68 ± 0.03 ^a^	nd ^c^	5.23 ± 0.09 ^b^	nd ^c^
1359	1-hexanol	nd ^b^	8.42 ± 0.18 ^a^	8.22 ± 0.11 ^a^	nd ^b^
1902	phenylethyl Alcohol	2.29 ± 0.07 ^d^	43.30 ± 0.27 ^a^	37.20 ± 0.56 ^b^	23.02 ± 0.48 ^c^
	tot	444.94 ± 0.9	2977.71 ± 107.2	2341.09 ± 66.63	18,430.49 ± 900.5
	Acids				
1445	acetic acid	29.71 ± 0.09 ^c^	306.49 ± 23.11 ^a^	171.42 ± 1.65 ^b^	nd ^d^
1580	2-methylpropanoic acid	2.32 ± 0.03 ^d^	22.82 ± 1.38 ^a^	12.95 ± 0.31 ^b^	7.48 ± 0.36 ^c^
1624	butanoic acid	nd ^b^	2.06 ± 0.13 ^a^	nd ^b^	nd ^b^
	tot	32.04 ± 0.06	333.08 ± 24.7	184.37 ± 1.34	7.48 ± 0.36
	Terpenoids				
1112	beta-pinene	6.39 ± 0.02 ^a^	3.71 ± 0.06 ^b^	2.61 ± 0.17 ^c^	1.99 ± 0.02 ^d^
1181	limonene	44.73 ± 2.29 ^a^	30.96 ± 1.87 ^b^	50.33 ± 0.73 ^a^	28.24 ± 1.98 ^b^
	tot	51.11 ± 2.27	34.67 ± 1.93	52.94 ± 0.56	30.23 ± 1.97

Abbreviations: nd, not detected; RI = retention index, identification via comparison with RI database for a high polar column for InnoWAX or similar stationary phases, [24]. Results are expressed as RAP = relative peak area (peak area of compound/peak area of internal standard) ⇥ 100 (RAP ± SD). Compounds with different letters in the same row are significantly different according to the Duncan’s test (*p* < 0.05).

**Table 5:** The enzyme profiles obtained by API ZYM on the selected native yeast strains YDSCYP4, YDSCYP5 and YSFWL3. The assignment values: 0 = negative reaction; 1, 2, 3 and 4 = intermediate reaction intensity; 5 = maximum reaction intensity.

	Enzyme	Activity of Yeast Strain YDSCYP4	Activity of Yeast Strain YDSCYP5	Activity of Yeast Strain YSFWL3
1	Control	0	0	0
2	Alkaline phosphatase	3	2	3
3	Esterase (C4)	5	4	4
4	Esterase Lipase (C8)	5	3	4
5	Lipase (C14)	3	0	0
6	Leucine-arylamidase	5	5	5
7	Valine-arylamidase	4	3	4
8	Cystine-arylamidase	2	1	4
9	Trypsin	0	0	0
10	Chymotrypsin	0	0	0
11	Acid phosphatase	5	4	5
12	Naphthol-AS-BL-phosphohydrolase	5	4	5
13	α-galactosidase	0	0	0
14	β-galactosidase	0	0	0
15	β-glucuronidase	0	0	0
16	α-glucosidase	3	2	2
17	β-glucosidase	0	0	2
18	α-glucoseaminidase	0	0	0
19	α-manosidase	0	0	0
20	α-fucosidase	0	0	0

## Data Availability

The original contributions presented in this study are included in the article/Supplementary Materials. Further inquiries can be directed to the corresponding author.

## Supplementary Materials

The following supporting information can be downloaded at: https://www.mdpi.com/article/10.3390/foods15081445/s1, Table S1. Detailed parameters of the ANCOVA models for each volatile compound. The table reports the estimated coefficients (Value), standard errors, and *p*-values for the interactions weight loss and yeast strains in Biomass A; Table S2. Detailed parameters of the ANCOVA models for each volatile compound. The table reports the estimated coefficients (Value), standard errors, and *p*-values for the interactions weight loss and yeast strains in Biomass B, static condition; Table S3. Two-way ANOVA results for selected volatile compounds during fermentation.
