# Supplementary material for: Agro-Food Waste for Isolation of Non-Conventional Yeasts and Flavor Compounds Production
Source: Foods. 2026 Apr 21;15(8):1445. doi: 10.3390/foods15081445 (PMC13114978; doi:10.3390/foods15081445)
Supplement: Supplementary file 1 [file foods-15-01445-s001.zip › Table S1.pdf]

Table S1. Detailed parameters of the ANCOVA models for each volatile compound. The table reports the estimated coefficients (Value), standard errors, and p-values for the interactions weight loss and yeast strains in Biomass A.

(2-methyl propanal):

Source	Value	Standard error	t	Pr > |t|	Lower bound (95%)	Upper bound (95%)	p-values signification codes
Intercept	3,747	9,217	0,407	0,691	-16,021	23,514	°
weight loss*biomass A-YP1 15d	-0,017	0,041	-0,406	0,691	-0,104	0,071	°
weight loss*biomass A-YP1 3d	-0,016	0,040	-0,406	0,691	-0,103	0,070	°
weight loss*biomass A-YP4 15d	-0,016	0,040	-0,406	0,691	-0,103	0,070	°
weight loss*biomass A-YP4 3d	-0,016	0,039	-0,406	0,691	-0,101	0,069	°
weight loss*biomass A-YP5 15d	-0,015	0,037	-0,406	0,691	-0,095	0,065	°
weight loss*biomass A-YP5 3d	-0,015	0,037	-0,406	0,691	-0,094	0,064	°
weight loss*biomass A-WL1 15d	0,222	0,039	5,737	**<0,0001**	0,139	0,304	***
weight loss*biomass A-WL1 3d	-0,015	0,038	-0,406	0,691	-0,097	0,066	°
weight loss*biomass A-WL2 15d	-0,017	0,041	-0,406	0,691	-0,104	0,071	°
weight loss*biomass A-WL2 3d	-0,016	0,039	-0,406	0,691	-0,100	0,068	°
weight loss*biomass A-WL3 15d	0,371	0,039	9,625	**<0,0001**	0,288	0,453	***
weight loss*biomass A-WL3 3d	-0,015	0,038	-0,406	0,691	-0,097	0,066	°
weight loss*biomass A-WL5 15d	0,321	0,038	8,350	**<0,0001**	0,239	0,404	***
weight loss*biomass A-WL5 3d	-0,015	0,038	-0,406	0,691	-0,096	0,066	°
weight loss*biomass A-CTR	-0,014	0,036	-0,406	0,691	-0,091	0,062	°

*Signification codes: 0 < *** < 0.001 < ** < 0.01 < * < 0.05 < . < 0.1 < ° < 1*

(2-methyl butanal):

Source	Value	Standard error	t	Pr > |t|	Lower bound (95%)	Upper bound (95%)	p-values signification codes
Intercept	-3,262	3,630	-0,899	0,384	-11,047	4,524	°
weight loss*biomass A-YP1 15d	0,014	0,016	0,898	0,384	-0,020	0,049	°

weight loss*biomass A-YP1 3d	0,014	0,016	0,898	0,384	-0,020	0,048	°
weight loss*biomass A-YP4 15d	0,014	0,016	0,898	0,384	-0,020	0,049	°
weight loss*biomass A-YP4 3d	0,014	0,016	0,898	0,384	-0,019	0,047	°
weight loss*biomass A-YP5 15d	0,013	0,015	0,898	0,384	-0,018	0,045	°
weight loss*biomass A-YP5 3d	0,013	0,015	0,898	0,384	-0,018	0,044	°
weight loss*biomass A-WL1 15d	0,014	0,015	0,898	0,384	-0,019	0,046	°
weight loss*biomass A-WL1 3d	0,013	0,015	0,898	0,384	-0,019	0,045	°
weight loss*biomass A-WL2 15d	0,014	0,016	0,898	0,384	-0,020	0,049	°
weight loss*biomass A-WL2 3d	0,014	0,016	0,898	0,384	-0,019	0,047	°
weight loss*biomass A-WL3 15d	0,014	0,015	0,898	0,384	-0,019	0,046	°
weight loss*biomass A-WL3 3d	0,013	0,015	0,898	0,384	-0,019	0,046	°
weight loss*biomass A-WL5 15d	0,412	0,015	27,203	**<0,0001**	0,380	0,445	***
weight loss*biomass A-WL5 3d	0,013	0,015	0,898	0,384	-0,019	0,045	°
weight loss*biomass A-CTR	0,013	0,014	0,898	0,384	-0,017	0,043	°

*Signification codes: 0 < *** < 0.001 < ** < 0.01 < * < 0.05 < . < 0.1 < ° < 1*

(Furfural):

Source	Value	Standard error	t	Pr > |t|	Lower bound (95%)	Upper bound (95%)	p-values signification codes
Intercept	-0,048	0,228	-0,211	0,836	-0,538	0,442	°
weight loss*biomass A-YP1 15d	0,000	0,001	0,211	0,836	-0,002	0,002	°
weight loss*biomass A-YP1 3d	0,000	0,001	0,211	0,836	-0,002	0,002	°
weight loss*biomass A-YP4 15d	0,000	0,001	0,211	0,836	-0,002	0,002	°
weight loss*biomass A-YP4 3d	0,000	0,001	0,211	0,836	-0,002	0,002	°
weight loss*biomass A-YP5 15d	0,000	0,001	0,211	0,836	-0,002	0,002	°
weight loss*biomass A-YP5 3d	0,000	0,001	0,211	0,836	-0,002	0,002	°
weight loss*biomass A-WL1 15d	0,000	0,001	0,211	0,836	-0,002	0,002	°
weight loss*biomass A-WL1 3d	0,000	0,001	0,211	0,836	-0,002	0,002	°
weight loss*biomass A-WL2 15d	0,000	0,001	0,211	0,836	-0,002	0,002	°
weight loss*biomass A-WL2 3d	0,000	0,001	0,211	0,836	-0,002	0,002	°
weight loss*biomass A-WL3 15d	0,000	0,001	0,211	0,836	-0,002	0,002	°

weight loss*biomass A-WL3 3d	0,000	0,001	0,211	0,836	-0,002	0,002	°
weight loss*biomass A-WL5 15d	0,000	0,001	0,211	0,836	-0,002	0,002	°
weight loss*biomass A-WL5 3d	0,000	0,001	0,211	0,836	-0,002	0,002	°
weight loss*biomass A-CTR	0,030	0,001	34,111	**<0,0001**	0,028	0,032	***

*Signification codes: 0 < *** < 0.001 < ** < 0.01 < * < 0.05 < . < 0.1 < ° < 1*

(Acetoin):

Source	Value	Standard error	t	Pr > |t|	Lower bound (95%)	Upper bound (95%)	p-values signification codes
Intercept	0,899	1,089	0,826	0,423	-1,436	3,235	°
weight loss*biomass A-YP1 15d	0,007	0,005	1,369	0,193	-0,004	0,017	°
weight loss*biomass A-YP1 3d	-0,004	0,005	-0,825	0,423	-0,014	0,006	°
weight loss*biomass A-YP4 15d	0,010	0,005	2,194	**0,046**	0,000	0,021	*
weight loss*biomass A-YP4 3d	-0,004	0,005	-0,825	0,423	-0,014	0,006	°
weight loss*biomass A-YP5 15d	-0,004	0,004	-0,825	0,423	-0,013	0,006	°
weight loss*biomass A-YP5 3d	-0,004	0,004	-0,825	0,423	-0,013	0,006	°
weight loss*biomass A-WL1 15d	0,061	0,005	13,407	**<0,0001**	0,051	0,071	***
weight loss*biomass A-WL1 3d	-0,004	0,004	-0,825	0,423	-0,013	0,006	°
weight loss*biomass A-WL2 15d	0,061	0,005	12,652	**<0,0001**	0,051	0,072	***
weight loss*biomass A-WL2 3d	-0,004	0,005	-0,825	0,423	-0,014	0,006	°
weight loss*biomass A-WL3 15d	0,007	0,005	1,487	0,159	-0,003	0,017	°
weight loss*biomass A-WL3 3d	-0,004	0,005	-0,825	0,423	-0,013	0,006	°
weight loss*biomass A-WL5 15d	0,024	0,005	5,278	**0,000**	0,014	0,034	***
weight loss*biomass A-WL5 3d	-0,004	0,004	-0,825	0,423	-0,013	0,006	°
weight loss*biomass A-CTR	-0,003	0,004	-0,825	0,423	-0,012	0,006	°

*Signification codes: 0 < *** < 0.001 < ** < 0.01 < * < 0.05 < . < 0.1 < ° < 1*

(Ethyl Acetate):

Source	Value	Standard error	t	Pr > |t|	Lower bound (95%)	Upper bound (95%)	p-values signification codes
Intercept	-581,657	738,803	-0,787	0,444	-2166,231	1002,916	°
weight loss*biomass A-YP1 15d	2,677	3,269	0,819	0,427	-4,334	9,687	°
weight loss*biomass A-YP1 3d	14,258	3,237	4,405	**0,001**	7,315	21,201	***
weight loss*biomass A-YP4 15d	2,553	3,245	0,787	0,445	-4,407	9,512	°
weight loss*biomass A-YP4 3d	14,708	3,166	4,645	**0,000**	7,917	21,499	***
weight loss*biomass A-YP5 15d	8,248	2,979	2,769	**0,015**	1,858	14,638	*
weight loss*biomass A-YP5 3d	5,779	2,952	1,958	0,070	-0,552	12,110	.
weight loss*biomass A-WL1 15d	5,618	3,096	1,815	0,091	-1,022	12,257	.
weight loss*biomass A-WL1 3d	2,767	3,041	0,910	0,378	-3,757	9,290	°
weight loss*biomass A-WL2 15d	5,823	3,282	1,774	0,098	-1,216	12,862	.
weight loss*biomass A-WL2 3d	3,196	3,158	1,012	0,329	-3,578	9,969	°
weight loss*biomass A-WL3 15d	4,532	3,086	1,469	0,164	-2,087	11,151	°
weight loss*biomass A-WL3 3d	2,554	3,057	0,836	0,417	-4,002	9,111	°
weight loss*biomass A-WL5 15d	4,262	3,085	1,382	0,189	-2,355	10,879	°
weight loss*biomass A-WL5 3d	2,563	3,032	0,845	0,412	-3,939	9,065	°
weight loss*biomass A-CTR	4,260	2,853	1,493	0,158	-1,859	10,378	°

*Signification codes: 0 < *** < 0.001 < ** < 0.01 < * < 0.05 < . < 0.1 < ° < 1*

(n-Propyl acetate):

Source	Value	Standard error	t	Pr > |t|	Lower bound (95%)	Upper bound (95%)	p-values signification codes
Intercept	38,601	44,697	0,864	0,402	-57,265	134,468	°
weight loss*biomass A-YP1 15d	-0,171	0,198	-0,863	0,403	-0,595	0,253	°
weight loss*biomass A-YP1 3d	0,171	0,196	0,875	0,396	-0,249	0,591	°
weight loss*biomass A-YP4 15d	-0,169	0,196	-0,863	0,403	-0,590	0,252	°
weight loss*biomass A-YP4 3d	0,445	0,192	2,321	**0,036**	0,034	0,855	*
weight loss*biomass A-YP5 15d	0,047	0,180	0,260	0,799	-0,340	0,433	°

weight loss*biomass A-YP5 3d	-0,154	0,179	-0,863	0,403	-0,537	0,229	°
weight loss*biomass A-WL1 15d	-0,162	0,187	-0,863	0,403	-0,563	0,240	°
weight loss*biomass A-WL1 3d	-0,159	0,184	-0,863	0,403	-0,553	0,236	°
weight loss*biomass A-WL2 15d	-0,171	0,199	-0,863	0,403	-0,597	0,255	°
weight loss*biomass A-WL2 3d	-0,165	0,191	-0,863	0,403	-0,575	0,245	°
weight loss*biomass A-WL3 15d	-0,161	0,187	-0,863	0,403	-0,562	0,239	°
weight loss*biomass A-WL3 3d	-0,160	0,185	-0,863	0,403	-0,556	0,237	°
weight loss*biomass A-WL5 15d	-0,161	0,187	-0,863	0,403	-0,561	0,239	°
weight loss*biomass A-WL5 3d	-0,158	0,183	-0,863	0,403	-0,552	0,235	°
weight loss*biomass A-CTR	-0,113	0,173	-0,654	0,524	-0,483	0,257	°

*Signification codes: 0 < *** < 0.001 < ** < 0.01 < * < 0.05 < . < 0.1 < ° < 1*

(butyl acetate):

Source	Value	Standard error	t	Pr > |t|	Lower bound (95%)	Upper bound (95%)	p-values signification codes
Intercept	-0,143	0,531	-0,270	0,791	-1,283	0,996	°
weight loss*biomass A-YP1 15d	0,001	0,002	0,270	0,791	-0,004	0,006	°
weight loss*biomass A-YP1 3d	0,073	0,002	31,437	**<0,0001**	0,068	0,078	***
weight loss*biomass A-YP4 15d	0,001	0,002	0,270	0,791	-0,004	0,006	°
weight loss*biomass A-YP4 3d	0,056	0,002	24,755	**<0,0001**	0,051	0,061	***
weight loss*biomass A-YP5 15d	0,001	0,002	0,270	0,791	-0,004	0,005	°
weight loss*biomass A-YP5 3d	0,001	0,002	0,270	0,791	-0,004	0,005	°
weight loss*biomass A-WL1 15d	0,001	0,002	0,270	0,791	-0,004	0,005	°
weight loss*biomass A-WL1 3d	0,001	0,002	0,270	0,791	-0,004	0,005	°
weight loss*biomass A-WL2 15d	0,001	0,002	0,270	0,791	-0,004	0,006	°
weight loss*biomass A-WL2 3d	0,001	0,002	0,270	0,791	-0,004	0,005	°
weight loss*biomass A-WL3 15d	0,001	0,002	0,270	0,791	-0,004	0,005	°
weight loss*biomass A-WL3 3d	0,001	0,002	0,270	0,791	-0,004	0,005	°
weight loss*biomass A-WL5 15d	0,001	0,002	0,270	0,791	-0,004	0,005	°
weight loss*biomass A-WL5 3d	0,001	0,002	0,270	0,791	-0,004	0,005	°
weight loss*biomass A-CTR	0,001	0,002	0,270	0,791	-0,004	0,005	°

*Signification codes: 0 < *** < 0.001 < ** < 0.01 < * < 0.05 < . < 0.1 < ° < 1*

(Isoamyl acetate):

Source	Value	Standard error	t	Pr > |t|	Lower bound (95%)	Upper bound (95%)	p-values signification codes
Intercept	-115,111	88,135	-1,306	0,213	-304,143	73,920	°
weight loss*biomass A-YP1 15d	0,509	0,390	1,305	0,213	-0,327	1,345	°
weight loss*biomass A-YP1 3d	1,500	0,386	3,884	**0,002**	0,672	2,328	**
weight loss*biomass A-YP4 15d	0,505	0,387	1,305	0,213	-0,325	1,335	°
weight loss*biomass A-YP4 3d	1,902	0,378	5,035	**0,000**	1,092	2,712	***
weight loss*biomass A-YP5 15d	2,147	0,355	6,040	**<0,0001**	1,384	2,909	***
weight loss*biomass A-YP5 3d	4,509	0,352	12,803	**<0,0001**	3,753	5,264	***
weight loss*biomass A-WL1 15d	1,602	0,369	4,339	**0,001**	0,810	2,394	***
weight loss*biomass A-WL1 3d	0,846	0,363	2,333	**0,035**	0,068	1,625	*
weight loss*biomass A-WL2 15d	0,544	0,392	1,389	0,186	-0,296	1,384	°
weight loss*biomass A-WL2 3d	1,128	0,377	2,995	**0,010**	0,320	1,936	**
weight loss*biomass A-WL3 15d	0,590	0,368	1,602	0,131	-0,200	1,380	°
weight loss*biomass A-WL3 3d	0,575	0,365	1,578	0,137	-0,207	1,358	°
weight loss*biomass A-WL5 15d	0,509	0,368	1,384	0,188	-0,280	1,299	°
weight loss*biomass A-WL5 3d	0,604	0,362	1,671	0,117	-0,171	1,380	°
weight loss*biomass A-CTR	0,461	0,340	1,354	0,197	-0,269	1,191	°

*Signification codes: 0 < *** < 0.001 < ** < 0.01 < * < 0.05 < . < 0.1 < ° < 1*

(Ethyl hexanoate):

Source	Value	Standard error	t	Pr > |t|	Lower bound (95%)	Upper bound (95%)	p-values signification codes
Intercept	13,813	10,887	1,269	0,225	-9,537	37,164	°
weight loss*biomass A-YP1 15d	-0,061	0,048	-1,268	0,226	-0,164	0,042	°
weight loss*biomass A-YP1 3d	-0,060	0,048	-1,268	0,226	-0,163	0,042	°

weight loss*biomass A-YP4 15d	-0,061	0,048	-1,268	0,226	-0,163	0,042	°
weight loss*biomass A-YP4 3d	-0,059	0,047	-1,268	0,226	-0,159	0,041	°
weight loss*biomass A-YP5 15d	-0,056	0,044	-1,268	0,226	-0,150	0,039	°
weight loss*biomass A-YP5 3d	-0,055	0,043	-1,268	0,226	-0,148	0,038	°
weight loss*biomass A-WL1 15d	0,016	0,046	0,347	0,734	-0,082	0,114	°
weight loss*biomass A-WL1 3d	-0,057	0,045	-1,268	0,226	-0,153	0,039	°
weight loss*biomass A-WL2 15d	-0,061	0,048	-1,268	0,226	-0,165	0,042	°
weight loss*biomass A-WL2 3d	0,078	0,047	1,680	0,115	-0,022	0,178	°
weight loss*biomass A-WL3 15d	-0,058	0,045	-1,268	0,226	-0,155	0,040	°
weight loss*biomass A-WL3 3d	0,291	0,045	6,463	**<0,0001**	0,195	0,388	***
weight loss*biomass A-WL5 15d	-0,058	0,045	-1,268	0,226	-0,155	0,040	°
weight loss*biomass A-WL5 3d	0,168	0,045	3,764	**0,002**	0,072	0,264	**
weight loss*biomass A-CTR	-0,053	0,042	-1,268	0,226	-0,143	0,037	°

*Signification codes: 0 < *** < 0.001 < ** < 0.01 < * < 0.05 < . < 0.1 < ° < 1*

(Hexyl acetate):

Source	Value	Standard error	t	Pr > |t|	Lower bound (95%)	Upper bound (95%)	p-values signification codes
Intercept	7,890	7,878	1,002	0,334	-9,007	24,787	°
weight loss*biomass A-YP1 15d	-0,035	0,035	-1,001	0,334	-0,110	0,040	°
weight loss*biomass A-YP1 3d	-0,035	0,035	-1,001	0,334	-0,109	0,039	°
weight loss*biomass A-YP4 15d	-0,035	0,035	-1,001	0,334	-0,109	0,040	°
weight loss*biomass A-YP4 3d	-0,029	0,034	-0,859	0,405	-0,101	0,043	°
weight loss*biomass A-YP5 15d	-0,018	0,032	-0,574	0,575	-0,086	0,050	°
weight loss*biomass A-YP5 3d	0,027	0,031	0,845	0,412	-0,041	0,094	°
weight loss*biomass A-WL1 15d	-0,033	0,033	-1,001	0,334	-0,104	0,038	°
weight loss*biomass A-WL1 3d	-0,032	0,032	-1,001	0,334	-0,102	0,037	°
weight loss*biomass A-WL2 15d	-0,035	0,035	-1,001	0,334	-0,110	0,040	°
weight loss*biomass A-WL2 3d	0,032	0,034	0,948	0,359	-0,040	0,104	°
weight loss*biomass A-WL3 15d	-0,033	0,033	-1,001	0,334	-0,104	0,038	°
weight loss*biomass A-WL3 3d	-0,024	0,033	-0,739	0,472	-0,094	0,046	°

weight loss*biomass A-WL5 15d	-0,033	0,033	-1,001	0,334	-0,103	0,038	°
weight loss*biomass A-WL5 3d	-0,032	0,032	-1,001	0,334	-0,102	0,037	°
weight loss*biomass A-CTR	-0,030	0,030	-1,001	0,334	-0,096	0,035	°

*Signification codes: 0 < *** < 0.001 < ** < 0.01 < * < 0.05 < . < 0.1 < ° < 1*

(Ethyl octanoate):

Source	Value	Standard error	t	Pr > |t|	Lower bound (95%)	Upper bound (95%)	p-values signification codes
Intercept	67,896	47,807	1,420	0,177	-34,640	170,432	°
weight loss*biomass A-YP1 15d	-0,275	0,212	-1,301	0,214	-0,729	0,178	°
weight loss*biomass A-YP1 3d	-0,297	0,209	-1,419	0,178	-0,747	0,152	°
weight loss*biomass A-YP4 15d	-0,298	0,210	-1,419	0,178	-0,748	0,152	°
weight loss*biomass A-YP4 3d	-0,291	0,205	-1,419	0,178	-0,730	0,149	°
weight loss*biomass A-YP5 15d	-0,274	0,193	-1,419	0,178	-0,687	0,140	°
weight loss*biomass A-YP5 3d	-0,225	0,191	-1,179	0,258	-0,635	0,185	°
weight loss*biomass A-WL1 15d	-0,284	0,200	-1,419	0,178	-0,714	0,145	°
weight loss*biomass A-WL1 3d	-0,279	0,197	-1,419	0,178	-0,701	0,143	°
weight loss*biomass A-WL2 15d	-0,301	0,212	-1,419	0,178	-0,757	0,154	°
weight loss*biomass A-WL2 3d	-0,252	0,204	-1,232	0,238	-0,690	0,187	°
weight loss*biomass A-WL3 15d	-0,283	0,200	-1,419	0,178	-0,712	0,145	°
weight loss*biomass A-WL3 3d	1,573	0,198	7,955	**<0,0001**	1,149	1,998	***
weight loss*biomass A-WL5 15d	-0,283	0,200	-1,419	0,178	-0,711	0,145	°
weight loss*biomass A-WL5 3d	0,153	0,196	0,782	0,447	-0,267	0,574	°
weight loss*biomass A-CTR	-0,262	0,185	-1,419	0,178	-0,658	0,134	°

*Signification codes: 0 < *** < 0.001 < ** < 0.01 < * < 0.05 < . < 0.1 < ° < 1*

(Ethyl decanoate):

Source	Value	Standard error	t	Pr > |t|	Lower bound (95%)	Upper bound (95%)	p-values signification codes

Intercept	-29,436	20,418	-1,442	0,171	-73,228	14,356	°
weight loss*biomass A-YP1 15d	0,130	0,090	1,441	0,172	-0,064	0,324	°
weight loss*biomass A-YP1 3d	0,129	0,089	1,441	0,172	-0,063	0,321	°
weight loss*biomass A-YP4 15d	0,129	0,090	1,441	0,172	-0,063	0,322	°
weight loss*biomass A-YP4 3d	0,126	0,088	1,441	0,172	-0,062	0,314	°
weight loss*biomass A-YP5 15d	0,119	0,082	1,441	0,172	-0,058	0,295	°
weight loss*biomass A-YP5 3d	0,118	0,082	1,441	0,172	-0,057	0,292	°
weight loss*biomass A-WL1 15d	0,123	0,086	1,441	0,172	-0,060	0,307	°
weight loss*biomass A-WL1 3d	0,121	0,084	1,441	0,172	-0,059	0,301	°
weight loss*biomass A-WL2 15d	0,131	0,091	1,441	0,172	-0,064	0,325	°
weight loss*biomass A-WL2 3d	0,126	0,087	1,441	0,172	-0,061	0,313	°
weight loss*biomass A-WL3 15d	0,123	0,085	1,441	0,172	-0,060	0,306	°
weight loss*biomass A-WL3 3d	0,956	0,084	11,313	**<0,0001**	0,775	1,137	***
weight loss*biomass A-WL5 15d	0,123	0,085	1,441	0,172	-0,060	0,306	°
weight loss*biomass A-WL5 3d	0,229	0,084	2,730	**0,016**	0,049	0,408	*
weight loss*biomass A-CTR	0,114	0,079	1,441	0,172	-0,056	0,283	°

*Signification codes: 0 < *** < 0.001 < ** < 0.01 < * < 0.05 < . < 0.1 < ° < 1*

(2-phenyl ethyl acetate):

Source	Value	Standard error	t	Pr > |t|	Lower bound (95%)	Upper bound (95%)	p-values signification codes
Intercept	5,899	12,430	0,475	0,642	-20,760	32,558	°
weight loss*biomass A-YP1 15d	-0,015	0,055	-0,270	0,791	-0,133	0,103	°
weight loss*biomass A-YP1 3d	0,040	0,054	0,742	0,470	-0,076	0,157	°
weight loss*biomass A-YP4 15d	-0,026	0,055	-0,474	0,643	-0,143	0,091	°
weight loss*biomass A-YP4 3d	0,089	0,053	1,673	0,117	-0,025	0,203	°
weight loss*biomass A-YP5 15d	0,018	0,050	0,358	0,726	-0,090	0,125	°
weight loss*biomass A-YP5 3d	0,505	0,050	10,173	**<0,0001**	0,399	0,612	***
weight loss*biomass A-WL1 15d	-0,025	0,052	-0,474	0,643	-0,136	0,087	°
weight loss*biomass A-WL1 3d	-0,024	0,051	-0,474	0,643	-0,134	0,085	°
weight loss*biomass A-WL2 15d	-0,026	0,055	-0,474	0,643	-0,145	0,092	°

weight loss*biomass A-WL2 3d	-0,025	0,053	-0,474	0,643	-0,139	0,089	°
weight loss*biomass A-WL3 15d	-0,025	0,052	-0,474	0,643	-0,136	0,087	°
weight loss*biomass A-WL3 3d	0,001	0,051	0,015	0,988	-0,110	0,111	°
weight loss*biomass A-WL5 15d	-0,025	0,052	-0,474	0,643	-0,136	0,087	°
weight loss*biomass A-WL5 3d	-0,024	0,051	-0,474	0,643	-0,134	0,085	°
weight loss*biomass A-CTR	-0,023	0,048	-0,474	0,643	-0,126	0,080	°

*Signification codes: 0 < *** < 0.001 < ** < 0.01 < * < 0.05 < . < 0.1 < ° < 1*

(Ethanol):

Source	Value	Standard error	t	Pr > |t|	Lower bound (95%)	Upper bound (95%)	p-values signification codes
Intercept	248,414	966,008	0,257	0,801	-1823,467	2320,295	°
weight loss*biomass A-YP1 15d	-1,091	4,274	-0,255	0,802	-10,257	8,075	°
weight loss*biomass A-YP1 3d	3,184	4,233	0,752	0,464	-5,894	12,263	°
weight loss*biomass A-YP4 15d	-1,090	4,243	-0,257	0,801	-10,190	8,010	°
weight loss*biomass A-YP4 3d	2,941	4,140	0,710	0,489	-5,938	11,820	°
weight loss*biomass A-YP5 15d	2,214	3,896	0,568	0,579	-6,141	10,570	°
weight loss*biomass A-YP5 3d	2,650	3,860	0,687	0,504	-5,628	10,928	°
weight loss*biomass A-WL1 15d	16,875	4,048	4,169	**0,001**	8,194	25,556	***
weight loss*biomass A-WL1 3d	14,915	3,977	3,751	**0,002**	6,386	23,445	**
weight loss*biomass A-WL2 15d	7,856	4,291	1,831	0,089	-1,348	17,060	.
weight loss*biomass A-WL2 3d	14,849	4,129	3,596	**0,003**	5,993	23,706	**
weight loss*biomass A-WL3 15d	9,899	4,035	2,453	**0,028**	1,244	18,553	*
weight loss*biomass A-WL3 3d	4,506	3,997	1,127	0,278	-4,066	13,079	°
weight loss*biomass A-WL5 15d	14,105	4,034	3,497	**0,004**	5,453	22,757	**
weight loss*biomass A-WL5 3d	4,752	3,964	1,199	0,251	-3,750	13,253	°
weight loss*biomass A-CTR	-0,640	3,730	-0,172	0,866	-8,640	7,360	°

*Signification codes: 0 < *** < 0.001 < ** < 0.01 < * < 0.05 < . < 0.1 < ° < 1*

(1-propanol):

Source	Value	Standard error	t	Pr > |t|	Lower bound (95%)	Upper bound (95%)	p-values signification codes
Intercept	-5,399	4,198	-1,286	0,219	-14,403	3,606	°
weight loss*biomass A-YP1 15d	0,024	0,019	1,285	0,220	-0,016	0,064	°
weight loss*biomass A-YP1 3d	0,024	0,018	1,285	0,220	-0,016	0,063	°
weight loss*biomass A-YP4 15d	0,024	0,018	1,285	0,220	-0,016	0,063	°
weight loss*biomass A-YP4 3d	0,023	0,018	1,285	0,220	-0,015	0,062	°
weight loss*biomass A-YP5 15d	0,022	0,017	1,285	0,220	-0,015	0,058	°
weight loss*biomass A-YP5 3d	0,022	0,017	1,285	0,220	-0,014	0,058	°
weight loss*biomass A-WL1 15d	0,076	0,018	4,312	**0,001**	0,038	0,114	***
weight loss*biomass A-WL1 3d	0,042	0,017	2,450	**0,028**	0,005	0,079	*
weight loss*biomass A-WL2 15d	0,089	0,019	4,769	**0,000**	0,049	0,129	***
weight loss*biomass A-WL2 3d	0,023	0,018	1,285	0,220	-0,015	0,062	°
weight loss*biomass A-WL3 15d	0,139	0,018	7,902	**<0,0001**	0,101	0,176	***
weight loss*biomass A-WL3 3d	0,022	0,017	1,285	0,220	-0,015	0,060	°
weight loss*biomass A-WL5 15d	0,037	0,018	2,136	0,051	0,000	0,075	.
weight loss*biomass A-WL5 3d	0,022	0,017	1,285	0,220	-0,015	0,059	°
weight loss*biomass A-CTR	0,021	0,016	1,285	0,220	-0,014	0,056	°

*Signification codes: 0 < *** < 0.001 < ** < 0.01 < * < 0.05 < . < 0.1 < ° < 1*

(isobutanol):

Source	Value	Standard error	t	Pr > |t|	Lower bound (95%)	Upper bound (95%)	p-values signification codes
Intercept	-5,338	12,004	-0,445	0,663	-31,084	20,409	°
weight loss*biomass A-YP1 15d	0,024	0,053	0,444	0,664	-0,090	0,138	°
weight loss*biomass A-YP1 3d	0,023	0,053	0,444	0,664	-0,089	0,136	°
weight loss*biomass A-YP4 15d	0,023	0,053	0,444	0,664	-0,090	0,137	°
weight loss*biomass A-YP4 3d	0,023	0,051	0,444	0,664	-0,087	0,133	°
weight loss*biomass A-YP5 15d	0,042	0,048	0,868	0,400	-0,062	0,146	°

weight loss*biomass A-YP5 3d	0,044	0,048	0,916	0,375	-0,059	0,147	°
weight loss*biomass A-WL1 15d	0,256	0,050	5,097	**0,000**	0,148	0,364	***
weight loss*biomass A-WL1 3d	0,151	0,049	3,057	**0,009**	0,045	0,257	**
weight loss*biomass A-WL2 15d	0,121	0,053	2,264	**0,040**	0,006	0,235	*
weight loss*biomass A-WL2 3d	0,072	0,051	1,401	0,183	-0,038	0,182	°
weight loss*biomass A-WL3 15d	0,191	0,050	3,817	**0,002**	0,084	0,299	**
weight loss*biomass A-WL3 3d	0,022	0,050	0,444	0,664	-0,084	0,129	°
weight loss*biomass A-WL5 15d	0,084	0,050	1,680	0,115	-0,023	0,192	°
weight loss*biomass A-WL5 3d	0,022	0,049	0,444	0,664	-0,084	0,128	°
weight loss*biomass A-CTR	0,021	0,046	0,444	0,664	-0,079	0,120	°

*Signification codes: 0 < *** < 0.001 < ** < 0.01 < * < 0.05 < . < 0.1 < ° < 1*

(1-butanol):

Source	Value	Standard error	t	Pr > |t|	Lower bound (95%)	Upper bound (95%)	p-values signification codes
Intercept	4,488	10,113	0,444	0,664	-17,202	26,177	°
weight loss*biomass A-YP1 15d	-0,020	0,045	-0,443	0,664	-0,116	0,076	°
weight loss*biomass A-YP1 3d	-0,020	0,044	-0,443	0,664	-0,115	0,075	°
weight loss*biomass A-YP4 15d	-0,020	0,044	-0,443	0,664	-0,115	0,076	°
weight loss*biomass A-YP4 3d	-0,019	0,043	-0,443	0,664	-0,112	0,074	°
weight loss*biomass A-YP5 15d	-0,018	0,041	-0,443	0,664	-0,106	0,069	°
weight loss*biomass A-YP5 3d	-0,018	0,040	-0,443	0,664	-0,105	0,069	°
weight loss*biomass A-WL1 15d	0,082	0,042	1,938	0,073	-0,009	0,173	.
weight loss*biomass A-WL1 3d	0,120	0,042	2,872	**0,012**	0,030	0,209	*
weight loss*biomass A-WL2 15d	-0,020	0,045	-0,443	0,664	-0,116	0,076	°
weight loss*biomass A-WL2 3d	-0,019	0,043	-0,443	0,664	-0,112	0,074	°
weight loss*biomass A-WL3 15d	0,060	0,042	1,432	0,174	-0,030	0,151	°
weight loss*biomass A-WL3 3d	-0,019	0,042	-0,443	0,664	-0,108	0,071	°
weight loss*biomass A-WL5 15d	0,099	0,042	2,338	**0,035**	0,008	0,189	*
weight loss*biomass A-WL5 3d	-0,018	0,041	-0,443	0,664	-0,107	0,071	°
weight loss*biomass A-CTR	-0,017	0,039	-0,443	0,664	-0,101	0,066	°

Signification codes: 0 < *** < 0.001 < ** < 0.01 < * < 0.05 < . < 0.1 < ° < 1

(isoamyl alcohol):

Source	Value	Standard error	t	Pr > |t|	Lower bound (95%)	Upper bound (95%)	p-values signification codes
Intercept	-3,548	1520,143	-0,002	0,998	-3263,930	3256,835	°
weight loss*biomass A-YP1 15d	0,069	6,725	0,010	0,992	-14,355	14,494	°
weight loss*biomass A-YP1 3d	0,144	6,661	0,022	0,983	-14,141	14,430	°
weight loss*biomass A-YP4 15d	0,100	6,677	0,015	0,988	-14,219	14,420	°
weight loss*biomass A-YP4 3d	0,124	6,515	0,019	0,985	-13,848	14,097	°
weight loss*biomass A-YP5 15d	0,070	6,130	0,011	0,991	-13,079	13,218	°
weight loss*biomass A-YP5 3d	0,014	6,074	0,002	0,998	-13,013	13,041	°
weight loss*biomass A-WL1 15d	6,597	6,369	1,036	0,318	-7,064	20,258	°
weight loss*biomass A-WL1 3d	4,701	6,258	0,751	0,465	-8,721	18,123	°
weight loss*biomass A-WL2 15d	5,317	6,753	0,787	0,444	-9,167	19,801	°
weight loss*biomass A-WL2 3d	3,898	6,498	0,600	0,558	-10,038	17,835	°
weight loss*biomass A-WL3 15d	5,033	6,350	0,793	0,441	-8,587	18,652	°
weight loss*biomass A-WL3 3d	0,470	6,290	0,075	0,941	-13,020	13,960	°
weight loss*biomass A-WL5 15d	5,156	6,348	0,812	0,430	-8,459	18,771	°
weight loss*biomass A-WL5 3d	0,709	6,238	0,114	0,911	-12,670	14,088	°
weight loss*biomass A-CTR	0,014	5,870	0,002	0,998	-12,576	12,603	°

Signification codes: 0 < *** < 0.001 < ** < 0.01 < * < 0.05 < . < 0.1 < ° < 1

(Benzyl alcohol):

Source	Value	Standard error	t	Pr > |t|	Lower bound (95%)	Upper bound (95%)	p-values signification codes
Intercept	13,390	12,183	1,099	0,290	-12,739	39,519	°
weight loss*biomass A-YP1 15d	-0,051	0,054	-0,940	0,363	-0,166	0,065	°

weight loss*biomass A-YP1 3d	-0,032	0,053	-0,593	0,562	-0,146	0,083	°
weight loss*biomass A-YP4 15d	-0,055	0,054	-1,037	0,317	-0,170	0,059	°
weight loss*biomass A-YP4 3d	-0,038	0,052	-0,721	0,483	-0,150	0,074	°
weight loss*biomass A-YP5 15d	-0,019	0,049	-0,384	0,707	-0,124	0,087	°
weight loss*biomass A-YP5 3d	-0,036	0,049	-0,740	0,471	-0,140	0,068	°
weight loss*biomass A-WL1 15d	-0,042	0,051	-0,831	0,420	-0,152	0,067	°
weight loss*biomass A-WL1 3d	-0,042	0,050	-0,843	0,413	-0,150	0,065	°
weight loss*biomass A-WL2 15d	-0,059	0,054	-1,098	0,291	-0,176	0,057	°
weight loss*biomass A-WL2 3d	-0,057	0,052	-1,098	0,291	-0,169	0,054	°
weight loss*biomass A-WL3 15d	-0,056	0,051	-1,098	0,291	-0,165	0,053	°
weight loss*biomass A-WL3 3d	0,008	0,050	0,158	0,877	-0,100	0,116	°
weight loss*biomass A-WL5 15d	-0,056	0,051	-1,098	0,291	-0,165	0,053	°
weight loss*biomass A-WL5 3d	0,001	0,050	0,024	0,981	-0,106	0,108	°
weight loss*biomass A-CTR	-0,011	0,047	-0,230	0,821	-0,112	0,090	°

*Signification codes: 0 < *** < 0.001 < ** < 0.01 < * < 0.05 < . < 0.1 < ° < 1*

(Phenyl ethanol):

Source	Value	Standard error	t	Pr > |t|	Lower bound (95%)	Upper bound (95%)	p-values signification codes
Intercept	33,961	34,087	0,996	0,336	-39,149	107,070	°
weight loss*biomass A-YP1 15d	-0,031	0,151	-0,207	0,839	-0,355	0,292	°
weight loss*biomass A-YP1 3d	-0,090	0,149	-0,605	0,555	-0,411	0,230	°
weight loss*biomass A-YP4 15d	-0,035	0,150	-0,234	0,819	-0,356	0,286	°
weight loss*biomass A-YP4 3d	-0,098	0,146	-0,671	0,513	-0,411	0,215	°
weight loss*biomass A-YP5 15d	-0,030	0,137	-0,219	0,830	-0,325	0,265	°
weight loss*biomass A-YP5 3d	-0,120	0,136	-0,877	0,395	-0,412	0,173	°
weight loss*biomass A-WL1 15d	-0,087	0,143	-0,608	0,553	-0,393	0,220	°
weight loss*biomass A-WL1 3d	-0,129	0,140	-0,921	0,373	-0,430	0,172	°
weight loss*biomass A-WL2 15d	-0,120	0,151	-0,794	0,441	-0,445	0,205	°
weight loss*biomass A-WL2 3d	-0,084	0,146	-0,578	0,572	-0,397	0,228	°
weight loss*biomass A-WL3 15d	-0,105	0,142	-0,737	0,473	-0,410	0,200	°

weight loss*biomass A-WL3 3d	-0,054	0,141	-0,380	0,709	-0,356	0,249	°
weight loss*biomass A-WL5 15d	-0,142	0,142	-0,996	0,336	-0,447	0,164	°
weight loss*biomass A-WL5 3d	-0,111	0,140	-0,794	0,440	-0,411	0,189	°
weight loss*biomass A-CTR	-0,131	0,132	-0,996	0,336	-0,413	0,151	°

*Signification codes: 0 < *** < 0.001 < ** < 0.01 < * < 0.05 < . < 0.1 < ° < 1*

(Acetic acid):

Source	Value	Standard error	t	Pr > |t|	Lower bound (95%)	Upper bound (95%)	p-values signification codes
Intercept	73,322	105,373	0,696	0,498	-152,681	299,325	°
weight loss*biomass A-YP1 15d	-0,324	0,466	-0,695	0,498	-1,324	0,676	°
weight loss*biomass A-YP1 3d	-0,050	0,462	-0,109	0,915	-1,041	0,940	°
weight loss*biomass A-YP4 15d	-0,315	0,463	-0,682	0,507	-1,308	0,677	°
weight loss*biomass A-YP4 3d	-0,050	0,452	-0,111	0,913	-1,019	0,918	°
weight loss*biomass A-YP5 15d	0,055	0,425	0,130	0,898	-0,856	0,967	°
weight loss*biomass A-YP5 3d	-0,239	0,421	-0,568	0,579	-1,142	0,664	°
weight loss*biomass A-WL1 15d	-0,137	0,442	-0,310	0,761	-1,084	0,810	°
weight loss*biomass A-WL1 3d	-0,073	0,434	-0,168	0,869	-1,003	0,857	°
weight loss*biomass A-WL2 15d	-0,179	0,468	-0,383	0,707	-1,183	0,825	°
weight loss*biomass A-WL2 3d	0,619	0,450	1,374	0,191	-0,347	1,585	°
weight loss*biomass A-WL3 15d	-0,144	0,440	-0,328	0,748	-1,088	0,800	°
weight loss*biomass A-WL3 3d	-0,250	0,436	-0,574	0,575	-1,185	0,685	°
weight loss*biomass A-WL5 15d	-0,156	0,440	-0,354	0,728	-1,100	0,788	°
weight loss*biomass A-WL5 3d	-0,268	0,432	-0,620	0,545	-1,195	0,659	°
weight loss*biomass A-CTR	-0,266	0,407	-0,654	0,524	-1,139	0,607	°

*Signification codes: 0 < *** < 0.001 < ** < 0.01 < * < 0.05 < . < 0.1 < ° < 1*

(2-methyl propanoic acid):

Source	Value	Standard error	t	Pr > |t|	Lower bound (95%)	Upper bound (95%)	p-values signification codes
Intercept	3,970	6,397	0,621	0,545	-9,750	17,690	°
weight loss*biomass A-YP1 15d	-0,003	0,028	-0,115	0,910	-0,064	0,057	°
weight loss*biomass A-YP1 3d	-0,013	0,028	-0,458	0,654	-0,073	0,047	°
weight loss*biomass A-YP4 15d	-0,004	0,028	-0,158	0,877	-0,065	0,056	°
weight loss*biomass A-YP4 3d	-0,017	0,027	-0,620	0,545	-0,076	0,042	°
weight loss*biomass A-YP5 15d	-0,006	0,026	-0,216	0,832	-0,061	0,050	°
weight loss*biomass A-YP5 3d	-0,016	0,026	-0,620	0,545	-0,071	0,039	°
weight loss*biomass A-WL1 15d	0,080	0,027	2,985	**0,010**	0,023	0,137	**
weight loss*biomass A-WL1 3d	0,003	0,026	0,111	0,913	-0,054	0,059	°
weight loss*biomass A-WL2 15d	0,033	0,028	1,159	0,266	-0,028	0,094	°
weight loss*biomass A-WL2 3d	0,033	0,027	1,203	0,249	-0,026	0,092	°
weight loss*biomass A-WL3 15d	0,017	0,027	0,644	0,530	-0,040	0,075	°
weight loss*biomass A-WL3 3d	-0,016	0,026	-0,620	0,545	-0,073	0,040	°
weight loss*biomass A-WL5 15d	0,015	0,027	0,552	0,589	-0,043	0,072	°
weight loss*biomass A-WL5 3d	-0,016	0,026	-0,620	0,545	-0,073	0,040	°
weight loss*biomass A-CTR	-0,015	0,025	-0,620	0,545	-0,068	0,038	°

*Signification codes: 0 < *** < 0.001 < ** < 0.01 < * < 0.05 < . < 0.1 < ° < 1*

(Butanoic acid):

Source	Value	Standard error	t	Pr > |t|	Lower bound (95%)	Upper bound (95%)	p-values signification codes
Intercept	-0,809	1,024	-0,790	0,443	-3,006	1,388	°
weight loss*biomass A-YP1 15d	0,004	0,005	0,789	0,443	-0,006	0,013	°
weight loss*biomass A-YP1 3d	0,004	0,004	0,789	0,443	-0,006	0,013	°
weight loss*biomass A-YP4 15d	0,004	0,004	0,789	0,443	-0,006	0,013	°
weight loss*biomass A-YP4 3d	0,003	0,004	0,789	0,443	-0,006	0,013	°
weight loss*biomass A-YP5 15d	0,003	0,004	0,789	0,443	-0,006	0,012	°
weight loss*biomass A-YP5 3d	0,003	0,004	0,789	0,443	-0,006	0,012	°

weight loss*biomass A-WL1 15d	0,022	0,004	5,149	**0,000**	0,013	0,031	***
weight loss*biomass A-WL1 3d	0,003	0,004	0,789	0,443	-0,006	0,012	°
weight loss*biomass A-WL2 15d	0,016	0,005	3,603	**0,003**	0,007	0,026	**
weight loss*biomass A-WL2 3d	0,045	0,004	10,261	**<0,0001**	0,036	0,054	***
weight loss*biomass A-WL3 15d	0,003	0,004	0,789	0,443	-0,006	0,013	°
weight loss*biomass A-WL3 3d	0,006	0,004	1,351	0,198	-0,003	0,015	°
weight loss*biomass A-WL5 15d	0,003	0,004	0,789	0,443	-0,006	0,013	°
weight loss*biomass A-WL5 3d	0,003	0,004	0,789	0,443	-0,006	0,012	°
weight loss*biomass A-CTR	0,003	0,004	0,789	0,443	-0,005	0,012	°

*Signification codes: 0 < *** < 0.001 < ** < 0.01 < * < 0.05 < . < 0.1 < ° < 1*

(2-methyl hexanoic acid):

Source	Value	Standard error	t	Pr > |t|	Lower bound (95%)	Upper bound (95%)	p-values signification codes
Intercept	11,915	13,962	0,853	0,408	-18,031	41,860	°
weight loss*biomass A-YP1 15d	-0,053	0,062	-0,853	0,408	-0,185	0,080	°
weight loss*biomass A-YP1 3d	-0,049	0,061	-0,794	0,441	-0,180	0,083	°
weight loss*biomass A-YP4 15d	0,090	0,061	1,468	0,164	-0,041	0,222	°
weight loss*biomass A-YP4 3d	-0,051	0,060	-0,853	0,408	-0,179	0,077	°
weight loss*biomass A-YP5 15d	-0,027	0,056	-0,488	0,633	-0,148	0,093	°
weight loss*biomass A-YP5 3d	-0,048	0,056	-0,853	0,408	-0,167	0,072	°
weight loss*biomass A-WL1 15d	-0,015	0,059	-0,258	0,800	-0,141	0,110	°
weight loss*biomass A-WL1 3d	-0,043	0,057	-0,755	0,463	-0,167	0,080	°
weight loss*biomass A-WL2 15d	-0,034	0,062	-0,549	0,592	-0,167	0,099	°
weight loss*biomass A-WL2 3d	-0,008	0,060	-0,132	0,897	-0,136	0,120	°
weight loss*biomass A-WL3 15d	-0,037	0,058	-0,631	0,538	-0,162	0,088	°
weight loss*biomass A-WL3 3d	-0,049	0,058	-0,853	0,408	-0,173	0,075	°
weight loss*biomass A-WL5 15d	-0,050	0,058	-0,853	0,408	-0,175	0,075	°
weight loss*biomass A-WL5 3d	-0,049	0,057	-0,853	0,408	-0,172	0,074	°
weight loss*biomass A-CTR	-0,046	0,054	-0,853	0,408	-0,162	0,070	°

*Signification codes: 0 < *** < 0.001 < ** < 0.01 < * < 0.05 < . < 0.1 < ° < 1*

(Octanoic acid):

Source	Value	Standard error	t	Pr > |t|	Lower bound (95%)	Upper bound (95%)	p-values signification codes
Intercept	0,014	0,095	0,151	0,882	-0,190	0,218	°
weight loss*biomass A-YP1 15d	0,000	0,000	-0,151	0,882	-0,001	0,001	°
weight loss*biomass A-YP1 3d	0,000	0,000	-0,151	0,882	-0,001	0,001	°
weight loss*biomass A-YP4 15d	0,000	0,000	-0,151	0,882	-0,001	0,001	°
weight loss*biomass A-YP4 3d	0,000	0,000	-0,151	0,882	-0,001	0,001	°
weight loss*biomass A-YP5 15d	0,000	0,000	-0,151	0,882	-0,001	0,001	°
weight loss*biomass A-YP5 3d	0,000	0,000	-0,151	0,882	-0,001	0,001	°
weight loss*biomass A-WL1 15d	0,000	0,000	-0,151	0,882	-0,001	0,001	°
weight loss*biomass A-WL1 3d	0,000	0,000	-0,151	0,882	-0,001	0,001	°
weight loss*biomass A-WL2 15d	0,000	0,000	-0,151	0,882	-0,001	0,001	°
weight loss*biomass A-WL2 3d	0,027	0,000	65,709	**<0,0001**	0,026	0,028	***
weight loss*biomass A-WL3 15d	0,000	0,000	-0,151	0,882	-0,001	0,001	°
weight loss*biomass A-WL3 3d	0,000	0,000	-0,151	0,882	-0,001	0,001	°
weight loss*biomass A-WL5 15d	0,000	0,000	-0,151	0,882	-0,001	0,001	°
weight loss*biomass A-WL5 3d	0,000	0,000	-0,151	0,882	-0,001	0,001	°
weight loss*biomass A-CTR	0,000	0,000	-0,151	0,882	-0,001	0,001	°

*Signification codes: 0 < *** < 0.001 < ** < 0.01 < * < 0.05 < . < 0.1 < ° < 1*

(beta myrcene):

Source	Value	Standard error	t	Pr > |t|	Lower bound (95%)	Upper bound (95%)	p-values signification codes
Intercept	1,144	1,306	0,876	0,396	-1,656	3,945	°
weight loss*biomass A-YP1 15d	0,001	0,006	0,221	0,829	-0,011	0,014	°
weight loss*biomass A-YP1 3d	0,005	0,006	0,874	0,397	-0,007	0,017	°
weight loss*biomass A-YP4 15d	-0,005	0,006	-0,876	0,396	-0,017	0,007	°

weight loss*biomass A-YP4 3d	0,030	0,006	5,331	**0,000**	0,018	0,042	***
weight loss*biomass A-YP5 15d	0,002	0,005	0,410	0,688	-0,009	0,013	°
weight loss*biomass A-YP5 3d	-0,002	0,005	-0,428	0,675	-0,013	0,009	°
weight loss*biomass A-WL1 15d	-0,005	0,005	-0,876	0,396	-0,017	0,007	°
weight loss*biomass A-WL1 3d	-0,005	0,005	-0,876	0,396	-0,016	0,007	°
weight loss*biomass A-WL2 15d	-0,005	0,006	-0,876	0,396	-0,018	0,007	°
weight loss*biomass A-WL2 3d	-0,005	0,006	-0,876	0,396	-0,017	0,007	°
weight loss*biomass A-WL3 15d	-0,005	0,005	-0,876	0,396	-0,016	0,007	°
weight loss*biomass A-WL3 3d	0,008	0,005	1,559	0,141	-0,003	0,020	°
weight loss*biomass A-WL5 15d	-0,005	0,005	-0,876	0,396	-0,016	0,007	°
weight loss*biomass A-WL5 3d	0,016	0,005	2,941	**0,011**	0,004	0,027	*
weight loss*biomass A-CTR	0,008	0,005	1,645	0,122	-0,003	0,019	°

*Signification codes: 0 < *** < 0.001 < ** < 0.01 < * < 0.05 < . < 0.1 < ° < 1*

(Limonene):

Source	Value	Standard error	t	Pr > |t|	Lower bound (95%)	Upper bound (95%)	p-values signification codes
Intercept	68,586	62,046	1,105	0,288	-64,489	201,660	°
weight loss*biomass A-YP1 15d	-0,273	0,275	-0,994	0,337	-0,862	0,316	°
weight loss*biomass A-YP1 3d	-0,263	0,272	-0,966	0,350	-0,846	0,320	°
weight loss*biomass A-YP4 15d	-0,267	0,273	-0,979	0,344	-0,851	0,318	°
weight loss*biomass A-YP4 3d	-0,193	0,266	-0,724	0,481	-0,763	0,378	°
weight loss*biomass A-YP5 15d	-0,254	0,250	-1,015	0,327	-0,791	0,283	°
weight loss*biomass A-YP5 3d	-0,253	0,248	-1,021	0,325	-0,785	0,279	°
weight loss*biomass A-WL1 15d	-0,287	0,260	-1,105	0,288	-0,845	0,270	°
weight loss*biomass A-WL1 3d	-0,282	0,255	-1,105	0,288	-0,830	0,266	°
weight loss*biomass A-WL2 15d	-0,304	0,276	-1,105	0,288	-0,896	0,287	°
weight loss*biomass A-WL2 3d	-0,141	0,265	-0,532	0,603	-0,710	0,428	°
weight loss*biomass A-WL3 15d	-0,286	0,259	-1,105	0,288	-0,842	0,270	°
weight loss*biomass A-WL3 3d	0,026	0,257	0,101	0,921	-0,525	0,577	°
weight loss*biomass A-WL5 15d	-0,286	0,259	-1,105	0,288	-0,842	0,270	°

weight loss*biomass A-WL5 3d	0,968	0,255	3,802	**0,002**	0,422	1,514	**
weight loss*biomass A-CTR	-0,212	0,240	-0,886	0,391	-0,726	0,302	°

*Signification codes: 0 < *** < 0.001 < ** < 0.01 < * < 0.05 < . < 0.1 < ° < 1*
