# Supplementary material for: Agro-Food Waste for Isolation of Non-Conventional Yeasts and Flavor Compounds Production
Source: Foods. 2026 Apr 21;15(8):1445. doi: 10.3390/foods15081445 (PMC13114978; doi:10.3390/foods15081445)
Supplement: Supplementary file 1 [file foods-15-01445-s001.zip › Table S2.pdf]

Table S2. Detailed parameters of the ANCOVA models for each volatile compound. The table reports the estimated coefficients (Value), standard errors, and p-values for the interactions weight loss and yeast strains in Biomass B, static condition.

(2-methyl-propanal):

Source	Value	Standard error	t	Pr > |t|	Lower bound (95%)	Upper bound (95%)	p-values signification codes
Intercept	0,231	3,366	0,069	0,946	-6,989	7,451	°
weight loss*yeast-CTR	0,031	0,014	2,132	0,051	0,000	0,062	.
weight loss*yeast-WL1 15d	-0,001	0,015	-0,069	0,946	-0,034	0,032	°
weight loss*yeast-WL1 3d	-0,001	0,015	-0,069	0,946	-0,033	0,031	°
weight loss*yeast-WL2 15d	-0,001	0,015	-0,069	0,946	-0,033	0,031	°
weight loss*yeast-WL2 3d	-0,001	0,014	-0,069	0,946	-0,032	0,030	°
weight loss*yeast-WL3 15d	0,095	0,014	6,683	**<0,0001**	0,064	0,125	***
weight loss*yeast-WL3 3d	-0,001	0,014	-0,069	0,946	-0,031	0,029	°
weight loss*yeast-WL5 15d	-0,001	0,014	-0,069	0,946	-0,032	0,030	°
weight loss*yeast-WL5 3d	-0,001	0,014	-0,069	0,946	-0,031	0,029	°
weight loss*yeast-YP1 15d	-0,001	0,015	-0,069	0,946	-0,034	0,031	°
weight loss*yeast-YP1 3d	-0,001	0,015	-0,069	0,946	-0,033	0,031	°
weight loss*yeast-YP4 15d	-0,001	0,015	-0,069	0,946	-0,033	0,031	°
weight loss*yeast-YP4 3d	-0,001	0,015	-0,069	0,946	-0,033	0,031	°
weight loss*yeast-YP5 15d	-0,001	0,014	-0,069	0,946	-0,031	0,029	°
weight loss*yeast-YP5 3d	-0,001	0,014	-0,069	0,946	-0,030	0,028	°

*Signification codes: 0 < *** < 0.001 < ** < 0.01 < * < 0.05 < . < 0.1 < ° < 1*

(2-methyl-butanal):

Source	Value	Standard error	t	Pr > |t|	Lower bound (95%)	Upper bound (95%)	p-values signification codes
Intercept	1,838	2,349	0,783	0,447	-3,199	6,876	°
weight loss*yeast-CTR	0,010	0,010	0,980	0,344	-0,012	0,031	°
weight loss*yeast-WL1 15d	-0,008	0,011	-0,781	0,448	-0,031	0,014	°

weight loss*yeast-WL1 3d	-0,008	0,010	-0,781	0,448	-0,030	0,014	°
weight loss*yeast-WL2 15d	-0,008	0,010	-0,781	0,448	-0,030	0,014	°
weight loss*yeast-WL2 3d	-0,008	0,010	-0,781	0,448	-0,030	0,014	°
weight loss*yeast-WL3 15d	-0,008	0,010	-0,781	0,448	-0,029	0,014	°
weight loss*yeast-WL3 3d	-0,008	0,010	-0,781	0,448	-0,029	0,013	°
weight loss*yeast-WL5 15d	-0,008	0,010	-0,781	0,448	-0,029	0,014	°
weight loss*yeast-WL5 3d	-0,008	0,010	-0,781	0,448	-0,029	0,013	°
weight loss*yeast-YP1 15d	-0,008	0,011	-0,781	0,448	-0,031	0,014	°
weight loss*yeast-YP1 3d	-0,008	0,010	-0,781	0,448	-0,030	0,014	°
weight loss*yeast-YP4 15d	-0,008	0,010	-0,781	0,448	-0,030	0,014	°
weight loss*yeast-YP4 3d	-0,005	0,010	-0,442	0,666	-0,027	0,017	°
weight loss*yeast-YP5 15d	-0,008	0,010	-0,781	0,448	-0,028	0,013	°
weight loss*yeast-YP5 3d	-0,007	0,010	-0,781	0,448	-0,028	0,013	°

*Signification codes: 0 < *** < 0.001 < ** < 0.01 < * < 0.05 < . < 0.1 < ° < 1*

(Furfural):

Source	Value	Standard error	t	Pr > |t|	Lower bound (95%)	Upper bound (95%)	p-values signification codes
Intercept	1,718	2,244	0,766	0,457	-3,095	6,532	°
weight loss*yeast-CTR	0,008	0,010	0,850	0,410	-0,012	0,029	°
weight loss*yeast-WL1 15d	-0,008	0,010	-0,764	0,458	-0,030	0,014	°
weight loss*yeast-WL1 3d	-0,008	0,010	-0,764	0,458	-0,029	0,014	°
weight loss*yeast-WL2 15d	-0,008	0,010	-0,764	0,458	-0,029	0,014	°
weight loss*yeast-WL2 3d	-0,007	0,010	-0,764	0,458	-0,028	0,013	°
weight loss*yeast-WL3 15d	-0,007	0,009	-0,764	0,458	-0,028	0,013	°
weight loss*yeast-WL3 3d	-0,007	0,009	-0,764	0,458	-0,027	0,013	°
weight loss*yeast-WL5 15d	-0,007	0,010	-0,764	0,458	-0,028	0,013	°
weight loss*yeast-WL5 3d	-0,007	0,009	-0,764	0,458	-0,027	0,013	°
weight loss*yeast-YP1 15d	-0,008	0,010	-0,764	0,458	-0,029	0,014	°
weight loss*yeast-YP1 3d	-0,008	0,010	-0,764	0,458	-0,029	0,014	°
weight loss*yeast-YP4 15d	-0,008	0,010	-0,764	0,458	-0,029	0,014	°

weight loss*yeast-YP4 3d	-0,007	0,010	-0,764	0,458	-0,028	0,014	°
weight loss*yeast-YP5 15d	-0,007	0,009	-0,764	0,458	-0,027	0,013	°
weight loss*yeast-YP5 3d	-0,007	0,009	-0,764	0,458	-0,026	0,013	°

*Signification codes: 0 < *** < 0.001 < ** < 0.01 < * < 0.05 < . < 0.1 < ° < 1*

(Acetoin):

Source	Value	Standard error	t	Pr > |t|	Lower bound (95%)	Upper bound (95%)	p-values signification codes
Intercept	-7,360	8,768	-0,839	0,415	-26,166	11,446	°
weight loss*yeast-CTR	0,073	0,037	1,938	0,073	-0,008	0,153	.
weight loss*yeast-WL1 15d	0,567	0,040	14,289	**<0,0001**	0,482	0,652	***
weight loss*yeast-WL1 3d	0,053	0,039	1,357	0,196	-0,031	0,136	°
weight loss*yeast-WL2 15d	0,039	0,039	1,019	0,325	-0,043	0,122	°
weight loss*yeast-WL2 3d	0,041	0,038	1,086	0,296	-0,040	0,122	°
weight loss*yeast-WL3 15d	0,287	0,037	7,767	**<0,0001**	0,208	0,367	***
weight loss*yeast-WL3 3d	0,054	0,036	1,497	0,157	-0,024	0,133	°
weight loss*yeast-WL5 15d	0,405	0,037	10,866	**<0,0001**	0,325	0,485	***
weight loss*yeast-WL5 3d	0,378	0,037	10,286	**<0,0001**	0,299	0,457	***
weight loss*yeast-YP1 15d	0,092	0,039	2,323	**0,036**	0,007	0,176	*
weight loss*yeast-YP1 3d	0,032	0,039	0,838	0,416	-0,051	0,115	°
weight loss*yeast-YP4 15d	0,032	0,039	0,838	0,416	-0,050	0,115	°
weight loss*yeast-YP4 3d	0,032	0,038	0,838	0,416	-0,050	0,114	°
weight loss*yeast-YP5 15d	0,030	0,036	0,838	0,416	-0,047	0,107	°
weight loss*yeast-YP5 3d	0,030	0,035	0,838	0,416	-0,046	0,106	°

*Signification codes: 0 < *** < 0.001 < ** < 0.01 < * < 0.05 < . < 0.1 < ° < 1*

(Ethyl Acetate):

Source	Value	Standard error	t	Pr > |t|	Lower bound (95%)	Upper bound (95%)	p-values signification codes

Intercept	-294,330	668,859	-0,440	0,667	- 1728,889	1140,229	°
weight loss*yeast-CTR	1,356	2,858	0,474	0,643	-4,773	7,485	°
weight loss*yeast-WL1 15d	2,339	3,026	0,773	0,453	-4,152	8,829	°
weight loss*yeast-WL1 3d	2,139	2,966	0,721	0,483	-4,221	8,500	°
weight loss*yeast-WL2 15d	1,987	2,944	0,675	0,511	-4,327	8,301	°
weight loss*yeast-WL2 3d	2,435	2,872	0,848	0,411	-3,725	8,596	°
weight loss*yeast-WL3 15d	1,638	2,823	0,580	0,571	-4,416	7,692	°
weight loss*yeast-WL3 3d	1,219	2,776	0,439	0,667	-4,734	7,172	°
weight loss*yeast-WL5 15d	2,142	2,841	0,754	0,463	-3,952	8,235	°
weight loss*yeast-WL5 3d	1,978	2,803	0,705	0,492	-4,035	7,990	°
weight loss*yeast-YP1 15d	2,735	3,008	0,909	0,379	-3,716	9,186	°
weight loss*yeast-YP1 3d	26,265	2,953	8,893	**<0,0001**	19,930	32,599	***
weight loss*yeast-YP4 15d	1,475	2,944	0,501	0,624	-4,840	7,790	°
weight loss*yeast-YP4 3d	23,566	2,920	8,072	**<0,0001**	17,305	29,828	***
weight loss*yeast-YP5 15d	16,202	2,742	5,908	**<0,0001**	10,320	22,084	***
weight loss*yeast-YP5 3d	15,140	2,707	5,593	**<0,0001**	9,334	20,946	***

*Signification codes: 0 < *** < 0.001 < ** < 0.01 < * < 0.05 < . < 0.1 < ° < 1*

(Ethyl propanoate):

Source	Value	Standard error	t	Pr > |t|	Lower bound (95%)	Upper bound (95%)	p-values signification codes
Intercept	-0,166	0,412	-0,404	0,692	-1,050	0,718	°
weight loss*yeast-CTR	0,001	0,002	0,403	0,693	-0,003	0,004	°
weight loss*yeast-WL1 15d	0,001	0,002	0,403	0,693	-0,003	0,005	°
weight loss*yeast-WL1 3d	0,001	0,002	0,403	0,693	-0,003	0,005	°
weight loss*yeast-WL2 15d	0,001	0,002	0,403	0,693	-0,003	0,005	°
weight loss*yeast-WL2 3d	0,001	0,002	0,403	0,693	-0,003	0,005	°
weight loss*yeast-WL3 15d	0,001	0,002	0,403	0,693	-0,003	0,004	°
weight loss*yeast-WL3 3d	0,001	0,002	0,403	0,693	-0,003	0,004	°
weight loss*yeast-WL5 15d	0,032	0,002	18,206	**<0,0001**	0,028	0,036	***

weight loss*yeast-WL5 3d	0,025	0,002	14,393	**<0,0001**	0,021	0,029	***
weight loss*yeast-YP1 15d	0,001	0,002	0,403	0,693	-0,003	0,005	°
weight loss*yeast-YP1 3d	0,001	0,002	0,403	0,693	-0,003	0,005	°
weight loss*yeast-YP4 15d	0,001	0,002	0,403	0,693	-0,003	0,005	°
weight loss*yeast-YP4 3d	0,001	0,002	0,403	0,693	-0,003	0,005	°
weight loss*yeast-YP5 15d	0,001	0,002	0,403	0,693	-0,003	0,004	°
weight loss*yeast-YP5 3d	0,001	0,002	0,403	0,693	-0,003	0,004	°

*Signification codes: 0 < *** < 0.001 < ** < 0.01 < * < 0.05 < . < 0.1 < ° < 1*

(n-Propyl acetate):

Source	Value	Standard error	t	Pr > |t|	Lower bound (95%)	Upper bound (95%)	p-values signification codes
Intercept	-55,623	56,637	-0,982	0,343	-177,097	65,851	°
weight loss*yeast-CTR	0,318	0,242	1,316	0,209	-0,201	0,837	°
weight loss*yeast-WL1 15d	0,251	0,256	0,980	0,344	-0,298	0,801	°
weight loss*yeast-WL1 3d	0,246	0,251	0,980	0,344	-0,293	0,785	°
weight loss*yeast-WL2 15d	0,244	0,249	0,980	0,344	-0,290	0,779	°
weight loss*yeast-WL2 3d	0,238	0,243	0,980	0,344	-0,283	0,760	°
weight loss*yeast-WL3 15d	0,234	0,239	0,980	0,344	-0,278	0,747	°
weight loss*yeast-WL3 3d	0,230	0,235	0,980	0,344	-0,274	0,734	°
weight loss*yeast-WL5 15d	0,236	0,241	0,980	0,344	-0,280	0,752	°
weight loss*yeast-WL5 3d	0,233	0,237	0,980	0,344	-0,277	0,742	°
weight loss*yeast-YP1 15d	0,250	0,255	0,980	0,344	-0,297	0,796	°
weight loss*yeast-YP1 3d	1,292	0,250	5,168	**0,000**	0,756	1,829	***
weight loss*yeast-YP4 15d	1,286	0,249	5,158	**0,000**	0,751	1,821	***
weight loss*yeast-YP4 3d	0,242	0,247	0,980	0,344	-0,288	0,773	°
weight loss*yeast-YP5 15d	0,820	0,232	3,530	**0,003**	0,322	1,318	**
weight loss*yeast-YP5 3d	0,225	0,229	0,980	0,344	-0,267	0,716	°

*Signification codes: 0 < *** < 0.001 < ** < 0.01 < * < 0.05 < . < 0.1 < ° < 1*

(Isobutyl acetate):

Source	Value	Standard error	t	Pr > |t|	Lower bound (95%)	Upper bound (95%)	p-values signification codes
Intercept	12,466	14,449	0,863	0,403	-18,523	43,456	°
weight loss*yeast-CTR	0,037	0,062	0,599	0,559	-0,095	0,169	°
weight loss*yeast-WL1 15d	-0,056	0,065	-0,861	0,404	-0,196	0,084	°
weight loss*yeast-WL1 3d	0,006	0,064	0,095	0,926	-0,131	0,143	°
weight loss*yeast-WL2 15d	-0,055	0,064	-0,861	0,404	-0,191	0,082	°
weight loss*yeast-WL2 3d	0,023	0,062	0,366	0,720	-0,110	0,156	°
weight loss*yeast-WL3 15d	-0,052	0,061	-0,861	0,404	-0,183	0,078	°
weight loss*yeast-WL3 3d	-0,052	0,060	-0,861	0,404	-0,180	0,077	°
weight loss*yeast-WL5 15d	-0,053	0,061	-0,861	0,404	-0,184	0,079	°
weight loss*yeast-WL5 3d	-0,052	0,061	-0,861	0,404	-0,182	0,078	°
weight loss*yeast-YP1 15d	-0,025	0,065	-0,381	0,709	-0,164	0,115	°
weight loss*yeast-YP1 3d	0,052	0,064	0,822	0,425	-0,084	0,189	°
weight loss*yeast-YP4 15d	0,034	0,064	0,529	0,605	-0,103	0,170	°
weight loss*yeast-YP4 3d	-0,054	0,063	-0,861	0,404	-0,190	0,081	°
weight loss*yeast-YP5 15d	0,369	0,059	6,237	**<0,0001**	0,242	0,497	***
weight loss*yeast-YP5 3d	0,227	0,058	3,884	**0,002**	0,102	0,353	**

*Signification codes: 0 < *** < 0.001 < ** < 0.01 < * < 0.05 < . < 0.1 < ° < 1*

(ethyl butanoate):

Source	Value	Standard error	t	Pr > |t|	Lower bound (95%)	Upper bound (95%)	p-values signification codes
Intercept	-0,107	0,117	-0,920	0,373	-0,357	0,143	°
weight loss*yeast-CTR	0,000	0,000	0,918	0,374	-0,001	0,002	°
weight loss*yeast-WL1 15d	0,007	0,001	13,584	**<0,0001**	0,006	0,008	***
weight loss*yeast-WL1 3d	0,004	0,001	8,411	**<0,0001**	0,003	0,005	***
weight loss*yeast-WL2 15d	0,003	0,001	6,750	**<0,0001**	0,002	0,005	***
weight loss*yeast-WL2 3d	0,006	0,001	11,771	**<0,0001**	0,005	0,007	***

weight loss*yeast-WL3 15d	0,004	0,000	8,016	**<0,0001**	0,003	0,005	***
weight loss*yeast-WL3 3d	0,000	0,000	0,918	0,374	-0,001	0,001	°
weight loss*yeast-WL5 15d	0,000	0,000	0,918	0,374	-0,001	0,002	°
weight loss*yeast-WL5 3d	0,000	0,000	0,918	0,374	-0,001	0,001	°
weight loss*yeast-YP1 15d	0,000	0,001	0,918	0,374	-0,001	0,002	°
weight loss*yeast-YP1 3d	0,000	0,001	0,918	0,374	-0,001	0,002	°
weight loss*yeast-YP4 15d	0,000	0,001	0,918	0,374	-0,001	0,002	°
weight loss*yeast-YP4 3d	0,000	0,001	0,918	0,374	-0,001	0,002	°
weight loss*yeast-YP5 15d	0,000	0,000	0,918	0,374	-0,001	0,001	°
weight loss*yeast-YP5 3d	0,000	0,000	0,918	0,374	-0,001	0,001	°

*Signification codes: 0 < *** < 0.001 < ** < 0.01 < * < 0.05 < . < 0.1 < ° < 1*

(Isoamyl acetate):

Source	Value	Standard error	t	Pr > |t|	Lower bound (95%)	Upper bound (95%)	p-values signification codes
Intercept	636,662	868,222	0,733	0,475	-1225,488	2498,813	°
weight loss*yeast-CTR	-2,485	3,709	-0,670	0,514	-10,441	5,471	°
weight loss*yeast-WL1 15d	-2,800	3,928	-0,713	0,488	-11,225	5,626	°
weight loss*yeast-WL1 3d	-1,461	3,850	-0,379	0,710	-9,717	6,796	°
weight loss*yeast-WL2 15d	-2,681	3,821	-0,702	0,494	-10,877	5,515	°
weight loss*yeast-WL2 3d	-1,023	3,728	-0,274	0,788	-9,020	6,973	°
weight loss*yeast-WL3 15d	-2,529	3,664	-0,690	0,501	-10,387	5,330	°
weight loss*yeast-WL3 3d	-1,964	3,603	-0,545	0,594	-9,692	5,763	°
weight loss*yeast-WL5 15d	-2,655	3,688	-0,720	0,483	-10,565	5,255	°
weight loss*yeast-WL5 3d	-2,663	3,639	-0,732	0,476	-10,467	5,142	°
weight loss*yeast-YP1 15d	-2,703	3,904	-0,692	0,500	-11,077	5,671	°
weight loss*yeast-YP1 3d	-0,808	3,834	-0,211	0,836	-9,030	7,415	°
weight loss*yeast-YP4 15d	-2,793	3,822	-0,731	0,477	-10,990	5,404	°
weight loss*yeast-YP4 3d	-0,632	3,790	-0,167	0,870	-8,761	7,496	°
weight loss*yeast-YP5 15d	11,871	3,560	3,335	**0,005**	4,236	19,506	**

weight loss*yeast-YP5 3d	12,934	3,514	3,681	**0,002**	5,397	20,470	**

*Signification codes: 0 < *** < 0.001 < ** < 0.01 < * < 0.05 < . < 0.1 < ° < 1*

(Isoamyl propanoate):

Source	Value	Standard error	t	Pr > |t|	Lower bound (95%)	Upper bound (95%)	p-values signification codes
Intercept	1,700	1,040	1,635	0,124	-0,531	3,931	°
weight loss*yeast-CTR	-0,007	0,004	-1,631	0,125	-0,017	0,002	°
weight loss*yeast-WL1 15d	-0,008	0,005	-1,631	0,125	-0,018	0,002	°
weight loss*yeast-WL1 3d	-0,008	0,005	-1,631	0,125	-0,017	0,002	°
weight loss*yeast-WL2 15d	-0,007	0,005	-1,631	0,125	-0,017	0,002	°
weight loss*yeast-WL2 3d	-0,007	0,004	-1,631	0,125	-0,017	0,002	°
weight loss*yeast-WL3 15d	-0,007	0,004	-1,631	0,125	-0,017	0,002	°
weight loss*yeast-WL3 3d	-0,007	0,004	-1,631	0,125	-0,016	0,002	°
weight loss*yeast-WL5 15d	-0,007	0,004	-1,631	0,125	-0,017	0,002	°
weight loss*yeast-WL5 3d	-0,007	0,004	-1,631	0,125	-0,016	0,002	°
weight loss*yeast-YP1 15d	-0,008	0,005	-1,631	0,125	-0,018	0,002	°
weight loss*yeast-YP1 3d	-0,007	0,005	-1,631	0,125	-0,017	0,002	°
weight loss*yeast-YP4 15d	0,028	0,005	6,145	**<0,0001**	0,018	0,038	***
weight loss*yeast-YP4 3d	-0,007	0,005	-1,631	0,125	-0,017	0,002	°
weight loss*yeast-YP5 15d	0,025	0,004	5,950	**<0,0001**	0,016	0,035	***
weight loss*yeast-YP5 3d	0,028	0,004	6,606	**<0,0001**	0,019	0,037	***

*Signification codes: 0 < *** < 0.001 < ** < 0.01 < * < 0.05 < . < 0.1 < ° < 1*

(Ethyl octanoate):

Source	Value	Standard error	t	Pr > |t|	Lower bound (95%)	Upper bound (95%)	p-values signification codes
Intercept	-1,220	1,320	-0,925	0,371	-4,051	1,610	°
weight loss*yeast-CTR	0,005	0,006	0,923	0,372	-0,007	0,017	°

weight loss*yeast-WL1 15d	0,035	0,006	5,789	**<0,0001**	0,022	0,047	***
weight loss*yeast-WL1 3d	0,039	0,006	6,732	**<0,0001**	0,027	0,052	***
weight loss*yeast-WL2 15d	0,005	0,006	0,923	0,372	-0,007	0,018	°
weight loss*yeast-WL2 3d	0,005	0,006	0,923	0,372	-0,007	0,017	°
weight loss*yeast-WL3 15d	0,005	0,006	0,923	0,372	-0,007	0,017	°
weight loss*yeast-WL3 3d	0,072	0,005	13,124	**<0,0001**	0,060	0,084	***
weight loss*yeast-WL5 15d	0,005	0,006	0,923	0,372	-0,007	0,017	°
weight loss*yeast-WL5 3d	0,033	0,006	5,939	**<0,0001**	0,021	0,045	***
weight loss*yeast-YP1 15d	0,005	0,006	0,923	0,372	-0,007	0,018	°
weight loss*yeast-YP1 3d	0,015	0,006	2,512	**0,025**	0,002	0,027	*
weight loss*yeast-YP4 15d	0,005	0,006	0,923	0,372	-0,007	0,018	°
weight loss*yeast-YP4 3d	0,005	0,006	0,923	0,372	-0,007	0,018	°
weight loss*yeast-YP5 15d	0,005	0,005	0,923	0,372	-0,007	0,017	°
weight loss*yeast-YP5 3d	0,005	0,005	0,923	0,372	-0,007	0,016	°

*Signification codes: 0 < *** < 0.001 < ** < 0.01 < * < 0.05 < . < 0.1 < ° < 1*

(Ethyl decanoate):

Source	Value	Standard error	t	Pr > |t|	Lower bound (95%)	Upper bound (95%)	p-values signification codes
Intercept	-0,785	0,745	-1,053	0,310	-2,383	0,813	°
weight loss*yeast-CTR	0,003	0,003	1,051	0,311	-0,003	0,010	°
weight loss*yeast-WL1 15d	0,004	0,003	1,051	0,311	-0,004	0,011	°
weight loss*yeast-WL1 3d	0,003	0,003	1,051	0,311	-0,004	0,011	°
weight loss*yeast-WL2 15d	0,003	0,003	1,051	0,311	-0,004	0,010	°
weight loss*yeast-WL2 3d	0,037	0,003	11,617	**<0,0001**	0,030	0,044	***
weight loss*yeast-WL3 15d	0,003	0,003	1,051	0,311	-0,003	0,010	°
weight loss*yeast-WL3 3d	0,028	0,003	9,210	**<0,0001**	0,022	0,035	***
weight loss*yeast-WL5 15d	0,003	0,003	1,051	0,311	-0,003	0,010	°
weight loss*yeast-WL5 3d	0,003	0,003	1,051	0,311	-0,003	0,010	°
weight loss*yeast-YP1 15d	0,004	0,003	1,051	0,311	-0,004	0,011	°
weight loss*yeast-YP1 3d	0,003	0,003	1,051	0,311	-0,004	0,011	°

weight loss*yeast-YP4 15d	0,003	0,003	1,051	0,311	-0,004	0,010	°
weight loss*yeast-YP4 3d	0,003	0,003	1,051	0,311	-0,004	0,010	°
weight loss*yeast-YP5 15d	0,003	0,003	1,051	0,311	-0,003	0,010	°
weight loss*yeast-YP5 3d	0,003	0,003	1,051	0,311	-0,003	0,010	°

*Signification codes: 0 < *** < 0.001 < ** < 0.01 < * < 0.05 < . < 0.1 < ° < 1*

(2-phenylethylacetate):

Source	Value	Standard error	t	Pr > |t|	Lower bound (95%)	Upper bound (95%)	p-values signification codes
Intercept	31,295	51,116	0,612	0,550	-78,338	140,927	°
weight loss*yeast-CTR	-0,003	0,218	-0,012	0,990	-0,471	0,466	°
weight loss*yeast-WL1 15d	-0,141	0,231	-0,611	0,551	-0,637	0,355	°
weight loss*yeast-WL1 3d	-0,103	0,227	-0,454	0,656	-0,589	0,383	°
weight loss*yeast-WL2 15d	-0,137	0,225	-0,611	0,551	-0,620	0,345	°
weight loss*yeast-WL2 3d	-0,114	0,220	-0,518	0,612	-0,585	0,357	°
weight loss*yeast-WL3 15d	-0,132	0,216	-0,611	0,551	-0,594	0,331	°
weight loss*yeast-WL3 3d	-0,113	0,212	-0,531	0,604	-0,568	0,342	°
weight loss*yeast-WL5 15d	-0,133	0,217	-0,611	0,551	-0,598	0,333	°
weight loss*yeast-WL5 3d	-0,131	0,214	-0,611	0,551	-0,590	0,329	°
weight loss*yeast-YP1 15d	-0,060	0,230	-0,261	0,798	-0,553	0,433	°
weight loss*yeast-YP1 3d	-0,097	0,226	-0,431	0,673	-0,581	0,387	°
weight loss*yeast-YP4 15d	-0,035	0,225	-0,154	0,880	-0,517	0,448	°
weight loss*yeast-YP4 3d	0,030	0,223	0,135	0,894	-0,448	0,509	°
weight loss*yeast-YP5 15d	1,494	0,210	7,130	**<0,0001**	1,045	1,944	***
weight loss*yeast-YP5 3d	0,560	0,207	2,709	**0,017**	0,117	1,004	*

*Signification codes: 0 < *** < 0.001 < ** < 0.01 < * < 0.05 < . < 0.1 < ° < 1*

(Ethanol):

Source	Value	Standard error	t	Pr > |t|	Lower bound (95%)	Upper bound (95%)	p-values signification codes
Intercept	-1174,841	1350,754	-0,870	0,399	-4071,919	1722,238	°
weight loss*yeast-CTR	6,091	5,771	1,055	0,309	-6,286	18,469	°
weight loss*yeast-WL1 15d	15,681	6,111	2,566	**0,022**	2,573	28,789	*
weight loss*yeast-WL1 3d	7,453	5,989	1,244	0,234	-5,393	20,298	°
weight loss*yeast-WL2 15d	13,956	5,945	2,347	**0,034**	1,205	26,707	*
weight loss*yeast-WL2 3d	7,226	5,801	1,246	0,233	-5,215	19,667	°
weight loss*yeast-WL3 15d	11,019	5,700	1,933	0,074	-1,208	23,245	.
weight loss*yeast-WL3 3d	8,367	5,605	1,493	0,158	-3,655	20,389	°
weight loss*yeast-WL5 15d	17,622	5,738	3,071	**0,008**	5,316	29,927	**
weight loss*yeast-WL5 3d	12,067	5,661	2,132	0,051	-0,075	24,210	.
weight loss*yeast-YP1 15d	5,272	6,074	0,868	0,400	-7,756	18,299	°
weight loss*yeast-YP1 3d	15,622	5,964	2,619	**0,020**	2,829	28,414	*
weight loss*yeast-YP4 15d	6,524	5,946	1,097	0,291	-6,229	19,277	°
weight loss*yeast-YP4 3d	14,827	5,896	2,515	**0,025**	2,181	27,472	*
weight loss*yeast-YP5 15d	11,423	5,538	2,063	0,058	-0,456	23,301	.
weight loss*yeast-YP5 3d	11,231	5,467	2,055	0,059	-0,494	22,956	.

*Signification codes: 0 < *** < 0.001 < ** < 0.01 < * < 0.05 < . < 0.1 < ° < 1*

(1-Propanol):

Source	Value	Standard error	t	Pr > |t|	Lower bound (95%)	Upper bound (95%)	p-values signification codes
Intercept	-2,848	3,586	-0,794	0,440	-10,539	4,844	°
weight loss*yeast-CTR	0,012	0,015	0,792	0,441	-0,021	0,045	°
weight loss*yeast-WL1 15d	0,027	0,016	1,682	0,115	-0,008	0,062	°
weight loss*yeast-WL1 3d	0,069	0,016	4,367	**0,001**	0,035	0,104	***
weight loss*yeast-WL2 15d	0,037	0,016	2,335	**0,035**	0,003	0,071	*
weight loss*yeast-WL2 3d	0,094	0,015	6,117	**<0,0001**	0,061	0,127	***

weight loss*yeast-WL3 15d	0,031	0,015	2,039	0,061	-0,002	0,063	.
weight loss*yeast-WL3 3d	0,070	0,015	4,674	**0,000**	0,038	0,101	***
weight loss*yeast-WL5 15d	0,012	0,015	0,792	0,441	-0,021	0,045	°
weight loss*yeast-WL5 3d	0,012	0,015	0,792	0,441	-0,020	0,044	°
weight loss*yeast-YP1 15d	0,013	0,016	0,792	0,441	-0,022	0,047	°
weight loss*yeast-YP1 3d	0,013	0,016	0,792	0,441	-0,021	0,047	°
weight loss*yeast-YP4 15d	0,013	0,016	0,792	0,441	-0,021	0,046	°
weight loss*yeast-YP4 3d	0,012	0,016	0,792	0,441	-0,021	0,046	°
weight loss*yeast-YP5 15d	0,012	0,015	0,792	0,441	-0,020	0,043	°
weight loss*yeast-YP5 3d	0,012	0,015	0,792	0,441	-0,020	0,043	°

*Signification codes: 0 < *** < 0.001 < ** < 0.01 < * < 0.05 < . < 0.1 < ° < 1*

(isobutanol):

Source	Value	Standard error	t	Pr > |t|	Lower bound (95%)	Upper bound (95%)	p-values signification codes
Intercept	32,362	20,541	1,575	0,137	-11,695	76,418	°
weight loss*yeast-CTR	-0,111	0,088	-1,269	0,225	-0,300	0,077	°
weight loss*yeast-WL1 15d	0,169	0,093	1,818	0,090	-0,030	0,368	.
weight loss*yeast-WL1 3d	0,223	0,091	2,443	**0,028**	0,027	0,418	*
weight loss*yeast-WL2 15d	0,087	0,090	0,965	0,351	-0,107	0,281	°
weight loss*yeast-WL2 3d	0,095	0,088	1,082	0,298	-0,094	0,285	°
weight loss*yeast-WL3 15d	0,430	0,087	4,961	**0,000**	0,244	0,616	***
weight loss*yeast-WL3 3d	0,233	0,085	2,729	**0,016**	0,050	0,415	*
weight loss*yeast-WL5 15d	0,095	0,087	1,084	0,297	-0,093	0,282	°
weight loss*yeast-WL5 3d	0,074	0,086	0,859	0,405	-0,111	0,259	°
weight loss*yeast-YP1 15d	0,067	0,092	0,728	0,478	-0,131	0,265	°
weight loss*yeast-YP1 3d	-0,117	0,091	-1,295	0,216	-0,312	0,077	°
weight loss*yeast-YP4 15d	-0,126	0,090	-1,388	0,187	-0,319	0,068	°
weight loss*yeast-YP4 3d	-0,045	0,090	-0,503	0,623	-0,237	0,147	°
weight loss*yeast-YP5 15d	0,048	0,084	0,568	0,579	-0,133	0,228	°
weight loss*yeast-YP5 3d	0,083	0,083	0,993	0,338	-0,096	0,261	°

*Signification codes: 0 < *** < 0.001 < ** < 0.01 < * < 0.05 < . < 0.1 < ° < 1*

(isoamyl alcohol):

Source	Value	Standard error	t	Pr > |t|	Lower bound (95%)	Upper bound (95%)	p-values signification codes
Intercept	-2549,899	814,202	-3,132	**0,007**	- 4296,189	-803,609	**
weight loss*yeast-CTR	11,512	3,479	3,310	**0,005**	4,052	18,973	**
weight loss*yeast-WL1 15d	20,781	3,684	5,641	**<0,0001**	12,880	28,682	***
weight loss*yeast-WL1 3d	19,901	3,610	5,512	**<0,0001**	12,158	27,644	***
weight loss*yeast-WL2 15d	21,783	3,584	6,078	**<0,0001**	14,097	29,469	***
weight loss*yeast-WL2 3d	17,903	3,496	5,120	**0,000**	10,404	25,402	***
weight loss*yeast-WL3 15d	23,162	3,436	6,741	**<0,0001**	15,793	30,532	***
weight loss*yeast-WL3 3d	18,421	3,379	5,452	**<0,0001**	11,174	25,668	***
weight loss*yeast-WL5 15d	20,565	3,458	5,946	**<0,0001**	13,148	27,983	***
weight loss*yeast-WL5 3d	18,868	3,413	5,529	**<0,0001**	11,549	26,187	***
weight loss*yeast-YP1 15d	11,846	3,661	3,236	**0,006**	3,993	19,699	**
weight loss*yeast-YP1 3d	12,603	3,595	3,505	**0,003**	4,892	20,314	**
weight loss*yeast-YP4 15d	11,758	3,584	3,281	**0,005**	4,071	19,445	**
weight loss*yeast-YP4 3d	12,890	3,554	3,627	**0,003**	5,267	20,512	**
weight loss*yeast-YP5 15d	10,846	3,338	3,249	**0,006**	3,686	18,006	**
weight loss*yeast-YP5 3d	10,876	3,295	3,301	**0,005**	3,809	17,944	**

*Signification codes: 0 < *** < 0.001 < ** < 0.01 < * < 0.05 < . < 0.1 < ° < 1*

(1-Pentanol):

Source	Value	Standard error	t	Pr > |t|	Lower bound (95%)	Upper bound (95%)	p-values signification codes
Intercept	2,574	1,784	1,443	0,171	-1,252	6,401	°

weight loss*yeast-CTR	-0,011	0,008	-1,440	0,172	-0,027	0,005	°
weight loss*yeast-WL1 15d	-0,012	0,008	-1,440	0,172	-0,029	0,006	°
weight loss*yeast-WL1 3d	-0,011	0,008	-1,440	0,172	-0,028	0,006	°
weight loss*yeast-WL2 15d	-0,011	0,008	-1,440	0,172	-0,028	0,006	°
weight loss*yeast-WL2 3d	-0,011	0,008	-1,440	0,172	-0,027	0,005	°
weight loss*yeast-WL3 15d	0,052	0,008	6,933	**<0,0001**	0,036	0,068	***
weight loss*yeast-WL3 3d	0,004	0,007	0,487	0,634	-0,012	0,019	°
weight loss*yeast-WL5 15d	-0,011	0,008	-1,440	0,172	-0,027	0,005	°
weight loss*yeast-WL5 3d	-0,011	0,007	-1,440	0,172	-0,027	0,005	°
weight loss*yeast-YP1 15d	0,027	0,008	3,388	**0,004**	0,010	0,044	**
weight loss*yeast-YP1 3d	-0,011	0,008	-1,440	0,172	-0,028	0,006	°
weight loss*yeast-YP4 15d	-0,011	0,008	-1,440	0,172	-0,028	0,006	°
weight loss*yeast-YP4 3d	0,023	0,008	2,959	**0,010**	0,006	0,040	*
weight loss*yeast-YP5 15d	0,018	0,007	2,502	**0,025**	0,003	0,034	*
weight loss*yeast-YP5 3d	-0,010	0,007	-1,440	0,172	-0,026	0,005	°

Signification codes: 0 < *** < 0.001 < ** < 0.01 < * < 0.05 < . < 0.1 < ° < 1

(Phenylethyl Alcohol):

Source	Value	Standard error	t	Pr > |t|	Lower bound (95%)	Upper bound (95%)	p-values signification codes
Intercept	-3,912	16,286	-0,240	0,814	-38,841	31,018	°
weight loss*yeast-CTR	0,027	0,070	0,384	0,707	-0,123	0,176	°
weight loss*yeast-WL1 15d	0,303	0,074	4,107	**0,001**	0,145	0,461	**
weight loss*yeast-WL1 3d	0,110	0,072	1,517	0,151	-0,045	0,264	°
weight loss*yeast-WL2 15d	0,247	0,072	3,445	**0,004**	0,093	0,401	**
weight loss*yeast-WL2 3d	0,090	0,070	1,291	0,218	-0,060	0,240	°
weight loss*yeast-WL3 15d	0,396	0,069	5,769	**<0,0001**	0,249	0,544	***
weight loss*yeast-WL3 3d	0,136	0,068	2,007	0,064	-0,009	0,281	.
weight loss*yeast-WL5 15d	0,280	0,069	4,050	**0,001**	0,132	0,429	**
weight loss*yeast-WL5 3d	0,120	0,068	1,762	0,100	-0,026	0,267	.
weight loss*yeast-YP1 15d	0,162	0,073	2,215	**0,044**	0,005	0,319	*

weight loss*yeast-YP1 3d	0,052	0,072	0,728	0,479	-0,102	0,207	°
weight loss*yeast-YP4 15d	0,047	0,072	0,658	0,521	-0,107	0,201	°
weight loss*yeast-YP4 3d	0,154	0,071	2,165	**0,048**	0,001	0,306	*
weight loss*yeast-YP5 15d	0,121	0,067	1,813	0,091	-0,022	0,264	.
weight loss*yeast-YP5 3d	0,039	0,066	0,592	0,563	-0,102	0,180	°

*Signification codes: 0 < *** < 0.001 < ** < 0.01 < * < 0.05 < . < 0.1 < ° < 1*

(Acetic acid):

Source	Value	Standard error	t	Pr > |t|	Lower bound (95%)	Upper bound (95%)	p-values signification codes
Intercept	-13,334	71,437	-0,187	0,855	-166,551	139,883	°
weight loss*yeast-CTR	0,184	0,305	0,602	0,557	-0,471	0,838	°
weight loss*yeast-WL1 15d	0,243	0,323	0,752	0,464	-0,450	0,936	°
weight loss*yeast-WL1 3d	0,287	0,317	0,907	0,380	-0,392	0,967	°
weight loss*yeast-WL2 15d	0,437	0,314	1,391	0,186	-0,237	1,112	°
weight loss*yeast-WL2 3d	0,797	0,307	2,597	**0,021**	0,139	1,455	*
weight loss*yeast-WL3 15d	0,628	0,301	2,082	0,056	-0,019	1,274	.
weight loss*yeast-WL3 3d	0,429	0,296	1,448	0,170	-0,207	1,065	°
weight loss*yeast-WL5 15d	0,211	0,303	0,694	0,499	-0,440	0,861	°
weight loss*yeast-WL5 3d	0,056	0,299	0,186	0,855	-0,586	0,698	°
weight loss*yeast-YP1 15d	0,213	0,321	0,662	0,519	-0,476	0,902	°
weight loss*yeast-YP1 3d	1,439	0,315	4,562	**0,000**	0,762	2,115	***
weight loss*yeast-YP4 15d	0,178	0,314	0,567	0,580	-0,496	0,853	°
weight loss*yeast-YP4 3d	1,146	0,312	3,675	**0,002**	0,477	1,815	**
weight loss*yeast-YP5 15d	0,149	0,293	0,507	0,620	-0,480	0,777	°
weight loss*yeast-YP5 3d	0,381	0,289	1,318	0,209	-0,239	1,001	°

*Signification codes: 0 < *** < 0.001 < ** < 0.01 < * < 0.05 < . < 0.1 < ° < 1*

(2-methylpropanoic acid):

Source	Value	Standard error	t	Pr > |t|	Lower bound (95%)	Upper bound (95%)	p-values signification codes
Intercept	-11,120	10,145	-1,096	0,292	-32,878	10,638	°
weight loss*yeast-CTR	0,065	0,043	1,500	0,156	-0,028	0,158	°
weight loss*yeast-WL1 15d	0,225	0,046	4,905	**0,000**	0,127	0,324	***
weight loss*yeast-WL1 3d	0,065	0,045	1,451	0,169	-0,031	0,162	°
weight loss*yeast-WL2 15d	0,343	0,045	7,693	**<0,0001**	0,248	0,439	***
weight loss*yeast-WL2 3d	0,067	0,044	1,535	0,147	-0,027	0,160	°
weight loss*yeast-WL3 15d	0,341	0,043	7,955	**<0,0001**	0,249	0,432	***
weight loss*yeast-WL3 3d	0,070	0,042	1,666	0,118	-0,020	0,160	°
weight loss*yeast-WL5 15d	0,112	0,043	2,604	**0,021**	0,020	0,205	*
weight loss*yeast-WL5 3d	0,107	0,043	2,507	**0,025**	0,015	0,198	*
weight loss*yeast-YP1 15d	0,116	0,046	2,552	**0,023**	0,019	0,214	*
weight loss*yeast-YP1 3d	0,060	0,045	1,339	0,202	-0,036	0,156	°
weight loss*yeast-YP4 15d	0,066	0,045	1,481	0,161	-0,030	0,162	°
weight loss*yeast-YP4 3d	0,072	0,044	1,617	0,128	-0,023	0,167	°
weight loss*yeast-YP5 15d	0,138	0,042	3,313	**0,005**	0,049	0,227	**
weight loss*yeast-YP5 3d	0,129	0,041	3,136	**0,007**	0,041	0,217	**

*Signification codes: 0 < *** < 0.001 < ** < 0.01 < * < 0.05 < . < 0.1 < ° < 1*

(Butanoic acid):

Source	Value	Standard error	t	Pr > |t|	Lower bound (95%)	Upper bound (95%)	p-values signification codes
Intercept	0,820	1,438	0,570	0,578	-2,265	3,905	°
weight loss*yeast-CTR	-0,003	0,006	-0,569	0,578	-0,017	0,010	°
weight loss*yeast-WL1 15d	0,016	0,007	2,400	**0,031**	0,002	0,030	*
weight loss*yeast-WL1 3d	-0,004	0,006	-0,569	0,578	-0,017	0,010	°
weight loss*yeast-WL2 15d	0,042	0,006	6,660	**<0,0001**	0,029	0,056	***
weight loss*yeast-WL2 3d	-0,004	0,006	-0,569	0,578	-0,017	0,010	°

weight loss*yeast-WL3 15d	0,065	0,006	10,640	**<0,0001**	0,052	0,078	***
weight loss*yeast-WL3 3d	-0,003	0,006	-0,569	0,578	-0,016	0,009	°
weight loss*yeast-WL5 15d	0,011	0,006	1,751	0,102	-0,002	0,024	°
weight loss*yeast-WL5 3d	0,007	0,006	1,172	0,261	-0,006	0,020	°
weight loss*yeast-YP1 15d	-0,004	0,006	-0,569	0,578	-0,018	0,010	°
weight loss*yeast-YP1 3d	-0,004	0,006	-0,569	0,578	-0,017	0,010	°
weight loss*yeast-YP4 15d	-0,004	0,006	-0,569	0,578	-0,017	0,010	°
weight loss*yeast-YP4 3d	-0,004	0,006	-0,569	0,578	-0,017	0,010	°
weight loss*yeast-YP5 15d	-0,003	0,006	-0,569	0,578	-0,016	0,009	°
weight loss*yeast-YP5 3d	-0,003	0,006	-0,569	0,578	-0,016	0,009	°

*Signification codes: 0 < *** < 0.001 < ** < 0.01 < * < 0.05 < . < 0.1 < ° < 1*

(beta-Pinene):

Source	Value	Standard error	t	Pr > |t|	Lower bound (95%)	Upper bound (95%)	p-values signification codes
Intercept	1,420	1,885	0,753	0,464	-2,623	5,463	°
weight loss*yeast-CTR	0,022	0,008	2,754	**0,016**	0,005	0,039	*
weight loss*yeast-WL1 15d	-0,006	0,009	-0,752	0,465	-0,025	0,012	°
weight loss*yeast-WL1 3d	-0,004	0,008	-0,484	0,636	-0,022	0,014	°
weight loss*yeast-WL2 15d	-0,006	0,008	-0,752	0,465	-0,024	0,012	°
weight loss*yeast-WL2 3d	-0,006	0,008	-0,752	0,465	-0,023	0,011	°
weight loss*yeast-WL3 15d	-0,006	0,008	-0,752	0,465	-0,023	0,011	°
weight loss*yeast-WL3 3d	-0,001	0,008	-0,186	0,855	-0,018	0,015	°
weight loss*yeast-WL5 15d	-0,006	0,008	-0,752	0,465	-0,023	0,011	°
weight loss*yeast-WL5 3d	-0,006	0,008	-0,752	0,465	-0,023	0,011	°
weight loss*yeast-YP1 15d	-0,006	0,008	-0,752	0,465	-0,025	0,012	°
weight loss*yeast-YP1 3d	-0,006	0,008	-0,752	0,465	-0,024	0,012	°
weight loss*yeast-YP4 15d	-0,006	0,008	-0,752	0,465	-0,024	0,012	°
weight loss*yeast-YP4 3d	-0,006	0,008	-0,752	0,465	-0,024	0,011	°
weight loss*yeast-YP5 15d	-0,006	0,008	-0,752	0,465	-0,022	0,011	°
weight loss*yeast-YP5 3d	-0,006	0,008	-0,752	0,465	-0,022	0,011	°

*Signification codes: 0 < *** < 0.001 < ** < 0.01 < * < 0.05 < . < 0.1 < ° < 1*

(Limonene):

Source	Value	Standard error	t	Pr > |t|	Lower bound (95%)	Upper bound (95%)	p-values signification codes
Intercept	0,043	0,933	0,046	0,964	-1,959	2,044	°
weight loss*yeast-CTR	0,014	0,004	3,598	**0,003**	0,006	0,023	**
weight loss*yeast-WL1 15d	0,000	0,004	-0,046	0,964	-0,009	0,009	°
weight loss*yeast-WL1 3d	0,007	0,004	1,595	0,133	-0,002	0,015	°
weight loss*yeast-WL2 15d	0,000	0,004	-0,046	0,964	-0,009	0,009	°
weight loss*yeast-WL2 3d	0,057	0,004	14,323	**<0,0001**	0,049	0,066	***
weight loss*yeast-WL3 15d	0,000	0,004	-0,046	0,964	-0,009	0,008	°
weight loss*yeast-WL3 3d	0,000	0,004	-0,046	0,964	-0,008	0,008	°
weight loss*yeast-WL5 15d	0,000	0,004	-0,046	0,964	-0,009	0,008	°
weight loss*yeast-WL5 3d	0,000	0,004	-0,046	0,964	-0,009	0,008	°
weight loss*yeast-YP1 15d	0,000	0,004	-0,046	0,964	-0,009	0,009	°
weight loss*yeast-YP1 3d	0,000	0,004	-0,046	0,964	-0,009	0,009	°
weight loss*yeast-YP4 15d	0,000	0,004	-0,046	0,964	-0,009	0,009	°
weight loss*yeast-YP4 3d	0,000	0,004	-0,046	0,964	-0,009	0,009	°
weight loss*yeast-YP5 15d	0,000	0,004	-0,046	0,964	-0,008	0,008	°
weight loss*yeast-YP5 3d	0,000	0,004	-0,046	0,964	-0,008	0,008	°

*Signification codes: 0 < *** < 0.001 < ** < 0.01 < * < 0.05 < . < 0.1 < ° < 1*
