# Supplementary material for: Agro-Food Waste for Isolation of Non-Conventional Yeasts and Flavor Compounds Production
Source: Foods. 2026 Apr 21;15(8):1445. doi: 10.3390/foods15081445 (PMC13114978; doi:10.3390/foods15081445)
Supplement: Supplementary file 1 [file foods-15-01445-s001.zip › Table S3.pdf]

Table S3. Two-way ANOVA results for selected volatile compounds during fermentation.

The table reports the significance levels (p-values) for the effects of Yeast strain (Y), Substrate (S), and their interaction (Y × S). This analysis was specifically performed on the volatile metabolites previously identified as significantly correlated with fermentation kinetics through ANCOVA ($p<0.05$ and $b>4.4$ in at least one condition). Values represent the mean± standard deviation of two biological replicates. Different lowercase letters within the same row indicate significant differences between experimental conditions according to Tukey's HSD test ($p<0.05$)

volatile compounds	yeasts	biomass A	Biomass B	yeasts (Y)	biomass (S)	interactions (YxS)
ethyl acetate	YP1 3d	2674,9±337,1 [a]	5665,2±134,5 [b]	***	***	***
	YP4 3d	2851,6±191,2 [a]	5117,1±78,7 [b]	***	***	**
	YP5 3d	865,8±14,1 [a]	3455,0±29,0 [b]	***	***	***
	YP5 15d	1465,5±23,0 [a]	3663,2±130,4 [b]	*	***	**
	WL1 3d	90,7±0,9 [a]	188,9±18,5 [b]	***	***	***
	WL2 3d	165,9±0,4 [a]	273,7±23,3 [b]	***	***	***
	WL1 15d	760,0±12,2 [a]	223,6±12,3 [b]	***	***	***
	WL2 15d	730,1±13,5 [a]	157,9±5,4 [b]	***	***	***
	WL3 15d	500,4±14,1 [a]	94,6±2,9 [b]	***	***	***
	WL5 15d	1439,0±16,4 [a]	210,6±14,3 [b]	***	***	***
isoamyl acetate	YP1 3d	227,5±4,4 [a]	453,3±8,1 [b]	***	***	***
	YP4 3d	328,9±2,1 [a]	491,7±20,5 [b]	***	***	***
	YP5 3d	1014,1±19,3 [a]	3840,8±176,4 [b]	***	***	***
	YP5 15d	417,5±15,8 [a]	3537,4±16,1 [b]	***	***	**
	WL1 3d	90,7±0,5 [a]	306,3±16,0 [b]	***	***	***
	WL2 3d	149,1±1,7 [a]	397,6±23,1 [b]	***	***	***
	WL1 15d	267,5±15,4 [a]	16,6±1,2 [b]	***	***	***
	WL2 15d	7,4±0,2 [a]	26,1±0,9 [b]	***	***	***
	WL3 15d	26,2±1,2 [a]	36,1±0,8 [b]	***	***	***
	WL5 15d	7,0±0,1 [a]	10,3±0,05 [b]	***	***	***
ethanol	YP1 3d	975,2±1,5 [a]	2368,3±198,6 [b]	***	***	**
	YP4 3d	935,0±2,2 [a]	2230,0±84,6 [b]	***	***	**
	YP5 3d	912,1±16,1 [a]	1606,3±2,6 [b]	***	***	**
	YP5 15d	798,2±27,8 [a]	1617,0±6,2 [b]	***	***	**
	WL1 3d	3842,4±59,8 [a]	509,2±36,5 [b]	***	***	***
	WL2 3d	3064,7±4,7 [a]	511,4±9,7 [b]	***	***	***
	WL1 15d	4279,3±28,0 [a]	2296,9±235,4 [b]	***	***	**
	WL2 15d	2017,0±17,3 [a]	1999,0±196,4 [b]	**	***	***
	WL3 15d	2620,0±42,1 [a]	1440,6±36,9 [b]	°	***	***
	WL5 15d	3627,0±110,8 [a]	2981,0±72,5 [b]	***	***	***
isoamyl alcohol	YP1 3d	29,4±0,8 [a]	310,2±20,1 [b]	***	°	°
	YP4 3d	25,4±1,6 [a]	409,7±24,9 [b]	***	°	°
	YP5 3d	0 [a]	143,4±2,7 [b]	***	°	°
	YP5 15d	13,7±1,0 [a]	101,2±0,4 [b]	***	°	°
	WL1 3d	1139,3±11,2 [a]	1947,3±61,7 [b]	***	°	**
	WL2 3d	908,8±19,1 [a]	1627,4±54,5 [b]	***	°	**
	WL1 15d	1572,2±1,4 [a]	2052,6±86,5 [b]	***	°	°
	WL2 15d	1194,0±22,1 [a]	2408,9±52,8 [b]	***	°	***
	WL3 15d	1202,5±21,6 [a]	2942,2±267,6 [b]	***	°	***
	WL5 15d	1231,9±34,1 [a]	2299,0±131,2 [b]	***	°	***

*Signification codes: 0 < *** < 0.001 < ** < 0.01 < * < 0.05 < . < 0.1 < ° < 1*
